# Cross-Sectional Age Differences in Canine Personality Traits; Influence of Breed, Sex, Previous Trauma, and Dog Obedience Tasks

**DOI:** 10.3389/fvets.2019.00493

**Published:** 2020-01-14

**Authors:** Lisa J. Wallis, Dóra Szabó, Enikő Kubinyi

**Affiliations:** Department of Ethology, Eötvös Loránd University, Budapest, Hungary

**Keywords:** dog personality, development, aging, cross-sectional, demographics, dog-human bond, trauma

## Abstract

The dog has been suggested as a possible model for personality development over the lifespan, however, we know little about how aging may shape their personality or the magnitude of age-related changes. Previously we established that aging influences multiple dog demographics, which could also affect how personality traits change across different age periods. A demographic questionnaire and the Dog Personality Questionnaire were completed for a cross-sectional sample of 1,207 adult dogs living in Hungary (M_age_ = 7.71, SD = 4.12), split into six different age groups. Results revealed three of the five factors showed significant age effects. Activity/Excitability decreased with age, and whilst Responsiveness to training also decreased, only dogs older than 12 years differed significantly from the other groups. Aggressiveness toward animals showed a quadratic trajectory peaking in dogs aged 6–10 years. The greatest magnitude of age-related change was detected between late senior and geriatric ages, likely caused by compensatory behavioral changes to biological aging and owner attitudes to aging. When the models were re-run including the other explanatory variables, age group was no longer significant for the Responsiveness to training trait. The amount of time spent interacting/playing with the owner partially mediated the relationship between age and this trait, implying that interventions to increase play and training motivation may alleviate the negative effects of aging on dogs' trainability. Fifteen out of 28 explanatory variables were significantly associated with at least one of the five factors [weight, breed (pure/mixed breed), sex, off-leash activity, diet, previous trauma, age of dog when arrived in the household, play, dog training activities, number of known commands and dog obedience tasks]. Similarly to humans, dogs that had previously experienced trauma scored higher in fearfulness and aggression. A higher level of basic obedience was linked to some desirable dog personality traits (lower Fearfulness and Aggression, and higher Activity/Excitability and Responsiveness to training). Regardless of the direction of this relationship, obedience is an important aspect contributing to dog personality questionnaires and the dog-owner relationship. This study is unique in that it considered a wide variety of demographic variables which are influenced by aging.

## Introduction

Although personality is defined as “behavioral differences that are stable across time and situations,” there is substantial cross-sectional evidence for mean personality trait change across the lifespan in humans (1). People tend to show increased self-confidence, warmth, self-control, and emotional stability with age, with changes occurring during young adulthood, middle age, and old age. Previous studies have also indicated substantial individual differences in changes; individuals display unique patterns of development at all life stages, which appear to be the result of specific life experiences (2). Work, marital, family, and educational experiences can all lead to changes in personality traits (3–6).

Cross-species comparison have been used to examine the origins and adaptive significance of specific personality traits. For example, Gosling and John (7) used the human Five factor model (FFM: openness to experience, conscientiousness, extraversion, agreeableness, and neuroticism) to compare the personality factors of dogs and 11 other non-human species. They found four of the five factors, and the canine analogs were labeled: Energy (analogous to human Extraversion), Affection (human Agreeableness), Emotional Reactivity (human Neuroticism), and Intelligence (human Openness/Intellect). The dog has been suggested as a possible model for human personality development, and the influence of personality on health (8–10). Dogs are observed by their owners on a daily basis, and biological, psychological, social, and health related events are often recorded. Their lifespan is much shorter than ours is, which means developmental studies can be performed in a shorter timeframe. Dogs share an evolutionary and developmental history with humans due to domestication. They are present in many households and are subject to the same environmental conditions. Thus, they can be tested using the same observations and experimental protocols (11). The high genetic variability and differing environmental experiences found in pet dogs makes them a good candidate to study individual differences and personality (12).

However, we know little about how aging and experience may shape personality in pet dogs (12). Over the last 20 years, much research has focused on studying personality in dogs, as they are common household pets around the world, and play important roles in human society, such as guide dogs, assistance dogs, therapy dogs, military and police dogs, and search and rescue dogs. The number of publications on personality in dogs has increased from roughly one per year in the late nineties to a current average of eight publications per year (Google scholar title word search). By far the most common method to assess personality in dogs is through owners' or care-takers individual ratings of individuals' personality traits on a Likert scale [with 1 being the least likely to exhibit the trait, and 5, or 7 as being the most likely (reviewed in Gartner (13)]. Evidence suggests that data collected through questionnaires can be accurate and consistent (14, 15) with demonstrated reliability and validity (13, 16–18). Owners can draw on their experience from a wide range of contexts and situations when they answer questions regarding their dogs' personality, while test batteries are strongly affected by the context in which they are performed, and do not necessarily reflect the dogs' behavior on a day-to-day basis. The most commonly used questionnaires include the Canine Behavioral Assessment and Research Questionnaire (C-BARQ) (19), the Monash Canine Personality Questionnaire (MCPQ/MCPQ-R) (20, 21), and the Dog Personality Questionnaire (DPQ) (22).

Most studies have concentrated on the early development up to 2 years, the predictability of certain early behavioral characteristics on adult behavior, or on senior and geriatric populations (23–26). Early experience has been found to have a long-term effect on the personality of dogs (27–29). Additionally, several studies have established differences in personality between individuals belonging to dog breeds or breed groups (30–32), as well as between the typical personality of pure breed and mixed breed dogs (33).

Besides the effects of early experience and breed, the most commonly reported factors that have been found to influence personality in dogs are age, sex, and reproductive status (34). Regarding age effects, younger dogs show higher boldness (35), sociability (36), companionability, energy, excitability, playfulness, active engagement (14), extraversion (21), and attentiveness (37, 38). The literature is contradictory about anxiety; while older dogs show higher calmness (36) and lower anxious/destructive behavior than younger dogs (39), neuroticism (a general measurement of fearfulness) was found to correlate positively with age (40). Touch sensitivity, fear of handling, fear of noises (14, 41), human and object fear (34), aggression toward dogs, and owner directed aggression (14, 42) also increase with age.

Inconsistencies may be due to the fact that different methods were used to obtain the trait scores, including one-word adjectives, complete sentence descriptions (with examples to set the trait in context), and/or different age-based groupings and age ranges of the samples. In addition, nearly all studies reported only linear age relationships, and many had only small effect sizes. The studies listed above did not look for quadratic relationships with age, and in most cases, only a few age groups were compared. Therefore, more detailed questionnaire studies regarding the influence of aging on mean level personality traits are necessary, particularly as the majority of past studies typically examined only a few personality traits, used dogs in working contexts, or only specific breeds, and only a handful of studies investigated dogs of all life stages (particularly those over 4 years of age). One recent study by Chopik and Weaver (43), is the first to use the validated Dog Personality Questionnaire (DPQ) (22) to examine the degree to which dog personality differs by age (including testing for quadratic relationships) whilst controlling for age differences in sex, breed (pure breed or mixed breed), reproductive status (intact/neutered), whether the dog has attended obedience training, and whether the owner trains their dog themselves or not. Although the sample was heavily biased toward undergraduate students (70% of the sample) and neutered dogs (87%), nevertheless a significant linear age effect was found for the factor Activity/Excitability, and quadratic effects were found for Responsiveness to training and Aggression toward animals. Older dogs were less active/excitable compared to younger dogs, and responsiveness to training and aggression toward other animals was highest among 6–8-year-old dogs.

Sex effects have been reported in 38% of studies [reviewed in Gartner (13)], however, reports are often conflicting. In general, results show that males have higher aggression (31, 34, 43, 44) and boldness (35, 36) and lower sociability (36) than females. Conversely, female dogs have higher fearfulness (40, 45), and lower dominance over dogs than males (14). Neuter status often complicates sex effects, due to the absence or presence of hormones. Intact dogs were found to be bolder (35) than neutered dogs. In addition, neutered dogs were found to be less calm (36), more aggressive, excitable and anxious (46) than entire male and female dogs. In one study, entire male Labrador retrievers showed higher owner aggression, and entire females higher trainability (34). The results of sex effects on personality are inconsistent, so further investigations are necessary with larger sample sizes, to clarify the patterns found, and determine the importance of sex effects in relation to other biological and environmental influences.

So far, personality differences have also been described with regards to coat color (34), body size [dog height is negatively associated with neuroticism, and positively with amicability (shorter dogs are considered more fearful and less sociable (21, 47))], training history [the most calm, trainable and sociable dogs were found to be those that have participated in three or more types of professional training (36)], and owner experience [experienced owners tend to have calmer and more trainable dogs (39)]. Several studies have even found correlations between the owner's and their dog's questionnaire-assessed personality traits (43, 48, 49).

Studies examining how behavior changes with age and/or breed, rarely take into account lifestyle demographic factors, which have the potential to influence both test battery and questionnaire results (50, 51). For example, environmental factors (such as housing condition; living in a flat, house, and/or garden) can mask, or even enhance genetically potentiated breed differences in personality (50). Physiological changes with age in the dog may also have an effect on the dogs' perceived personality. Starling et al. (35) suggested that a sharp decrease in the personality trait boldness, in dogs aged over 13 years might be explained by age-related degenerative conditions, such as arthritis. Older dogs may suffer from physical pain and discomfort, which may cause them to take fewer risks and to become less inclined to interact with other dogs or people. Therefore, when examining personality in dogs over all life stages, it is important to include a wide range of dog demographic, health, and environmental factors.

The aim of this study was to investigate the effects of age on personality in a cross-sectional Hungarian sample. Additionally, we explored which other factors are associated with dog personality. We measured personality using the Dog Personality Questionnaire (DPQ) (22), as it has been shown to demonstrate reliability and validity, and has been used in numerous studies to measure personality in dogs via owner report (25, 52–54). Additionally, it has been found to be the more reliable and trustworthy questionnaire in comparison to C-BARQ and MCPQ-R (14), and it achieved a slightly higher average mean consensus estimate of inter-rater reliability than the MCPQ-R (0.54 vs. 0.45) (15). From previous studies, we predicted a strong influence of dog age on dog personality. Since few studies report the magnitude of age-related change, we analyzed how much the personality traits change across different age periods (mean-level changes) and explore at what age changes in personality traits most prominently occur. Additionally, Purebred dogs were predicted to be rated as less fearful and aggressive than mixed breeds, male dogs less fearful and more aggressive than females, reproductively intact dogs less fearful than neutered, and finally, shared activities and training was predicted to increase responsiveness to training and decrease fearfulness and aggression.

## Methods

### Ethical Statement

Data were collected from Hungarian dog owners via an online questionnaire. Owners gave their informed consent for the data to be used for scientific purposes in an introductory letter, before filling out the questionnaire voluntarily and anonymously.

### Subjects

One thousand three hundred and sixty five Hungarian dog owners filled out an online questionnaire, which was advertised on the Eötvös Loránd University Department of Ethology's homepage (http://kutyaetologia.elte.hu), on the Facebook page “Családi Kutya Program,” and on the group “Kutyaetológia.” The questionnaire was available from the middle of May to the beginning of July 2016. Dogs aged under 1 year were excluded from the full sample of 1365, as previous research has suggested that their behavior does not remain stable over time (55). Duplicate entries and entries with missing information were deleted, which resulted in data from a total of 1207 individual dogs. The final sample consisted of 66% pure breeds, 54% females, of which 17% were intact, and 37% were neutered (26% intact males and 20% neutered males). The descriptive statistics of the sample are presented in Table 1.

**Table 1:** Descriptive statistics of the subjects, including sex, age, breed group, weight, and height information.

**Breed**	**Total count (%)**	**Sex** ***N*** **(%)**		**Age in months (Mean ± SD)**	**Weight in kg (Mean ± SD)**	**Height in cm (Mean ± SD)**
		**Male**	**Female**			
Mixed breeds	417 (34.5)	192 (15.9)	225 (18.6)	97.50 ± 51.05	20.10 ± 11.02	43.41 ± 13.15
Pure breeds	790 (65.5)	365 (30.2)	425 (35.2)	89.80 ± 48.36	21.13 ± 13.88	43.56 ± 15.33
Grand total	1207	557 (46.1)	650 (53.9)	92.46 ± 49.42	20.77 ± 12.97	43.51 ± 14.61

### Procedure

The on-line questionnaire contained three sections—the demographic data of the dogs and their owners, questions relating to the dogs' personality, and questions concerning possible age-related changes in cognition, impulsivity and interspecific communication (results from this final questionnaire are presented in a forthcoming publication). The “Demographic Questionnaire” collected basic information regarding the demographic attributes of the dog and the owner and social attributes of their interactions. Details from the demographic questionnaire were previously reported in Wallis et al. (56), where we examined the descriptive statistics of the variables, and whether the proportion of the dogs allocated to each category of the demographic variables varied among the dog age groups. Three continuous variables were collected from the owners: the dog's current weight (in kg), height at the shoulder (in cm), and age (in months) (Table 1). The rest of the variables were categorical, and the main descriptive statistics of the subset of 1207 dogs and their owners are presented in Table S1. In addition to reporting the age in months of the dogs, we also allocated the dogs to six age groups, which would allow us to examine non-linear relationships with age. For the age classifications we used: early adulthood (>1–3 years) *N* = 185, middle age (>3–6 years) *N* = 251, late adulthood (>6–8 years) *N* = 191, senior (>8–10 years) *N* = 202, late senior (>10–12 years) *N* = 170, and geriatric (>12 years) *N* = 208. These age groups were similar to those used in Wallis et al. (38), reflecting the developmental periods in the Border collie.

To measure dog personality traits, we used the “Dog Personality Questionnaire” (DPQ) as it has been shown to demonstrate reliability and validity, and has been used in numerous studies to measure personality in dogs via owner report (22). For details of the items used, please refer to Table S2.

### Statistical Analysis

#### Generation of Factor Scores and Assessment of Reliability

We used the short form of the DPQ, which consisted of 45 items that made up a five-factor solution. We translated the questionnaire into Hungarian, and then back translated into English, to ensure that each items content was preserved. Instead of using a Likert scale with 7 options [as was used in Jones (22)], we simplified the available responses, by reducing the scale to 5 possibilities, in harmony with the other scales of the questionnaires utilized. Owners scored the amount they agreed with each statement from 1—I do not agree at all with the statement, to 5—I fully agree. To calculate the facet and factor scores we used the Scoring Key for the DPQ Short Form provided by the author. The scores for each relevant raw item were averaged to create the facet scores. The factor scores were produced by averaging the scores of the facets that made up each specific factor. If one item score was missing, then no facet or factor score was calculated for that individual. The five factors were labeled by Jones as “Fearfulness, Aggression toward People, Aggression toward Animals, Activity/Excitability, and Responsiveness to Training.” Cronbach's alpha was calculated to assess the internal reliability of the extracted factors (57). The five factors were divided into facets: “Fearfulness” was composed of “Fear of people,” “Non-social fear,” “Fear of dogs” and “Fear of handling.” “Aggression toward people” was divided into “General aggression” and “Situational aggression.” “Activity/Excitability” was divided into “Excitability,” “Playfulness,” “Active engagement” and “Companionability.” “Responsiveness to training” was composed of “Trainability” and “Controllability.” The last factor, “Aggression toward animals,” contained “Aggression toward Dogs,” “Prey Drive” and “Dominance over Other Dogs” (22). Please see Supplementary Materials for a copy of the questionnaire and scoring key (Tables S2, S3), and for results of the age analyses of the facets.

Correlations between the factor scores were calculated using Spearman's rho as the data were not normally distributed. Results are displayed in Table S4.

#### Statistical Models to Determine the Effects of the Demographic Variables

Statistical analyses were run on the reduced dataset of 1,207 individuals and performed in R 3.3.2 (58). The five factors from the DPQ were transformed using the boxcox power transformation [Package “MASS,” (59)] to fulfill the assumptions of normality and homogeneity of variance. Separate linear models were first calculated with age as a categorical variable to look for specific differences between age groups on the five factors of the DPQ. *Post hoc* Tukey tests were run when significant age group differences were found (*p* values were adjusted for multiple comparisons using the single-step method. “Single-step” implements adjusted *p* values based on the joint normal or t distribution of the linear function). Mean level changes across the different age periods are reported, including a brief discussion of the age periods when changes in personality traits predominately occur. Then additional models were run with weight and height included as covariates, and all of the rest of the variables as fixed factors (age group, breed, sex, neuter status, sensory problems, off-leash activity, body condition score, food, vitamins, trauma, health problems, medication, owner age, owner experience, how many other dogs in household, how many people in household, child, dog age when arrived, get dog, where dog is kept, dog obedience tasks, play, commands, dog training activities, time spent alone, and dog behavior changed (for descriptions of categories see Table S1). The aim of these linear model analyses was to investigate (1) associations between personality traits and the investigated variables (e.g., demographics of both dog and owner), and (2) to examine whether the behavioral differences between the dog age groups remained significant after controlling for the differences in the other explanatory variables. Due to the large number of predictors used in the models (28 explanatory variables in total), only main effects were analyzed, and we did not examine interactions.

Normality and homoscedasticity were assessed via residuals' distribution charts and plots of residuals against fitted values. Due to the large number of variables retained in the models, the Benjamini–Hochberg procedure was utilized to control for the false discovery rate [FDR, (60)]. Most of the categorical variables used were ordinal, which allowed group comparisons to the smallest or lowest category. However, *post hoc* Tukey tests were run on the nominal variables where significant group differences were found (again *p* values were adjusted for multiple comparisons using the single-step method). Results are detailed in Table S4. To analyse the effect of outliers, any outliers of *z* scores of > ± 3 were removed from the analysis, and the models re-run.

A mediation model was proposed in order to better explain the mechanism or process that underlies the relationship between personality and dog age, if a previously significant age effect was no longer detectable in the second model including the other explanatory variables. Please note that mediation analysis does not imply a causal relationship. In the case where multiple significant explanatory variables were present in the model, we chose the variable that had the greatest variance explained by dog age [this was determined previously in Wallis et al. (56)]. By implementing the Mediation package in R (61) we estimated the average causal mediation effect (ACME) and the average direct effect (ADE). First, we fitted the mediator model, where the measure of the relevant explanatory variable is modeled as a function of dog age group and confounding variables [weight, height, breed, sex, neuter status, etc. (see Table 4 for full list of final model variables)]. Next, we modeled the outcome variable, including the mediator, age group, and the same set of confounding variables as those used in the mediator model. We then used the mediate function to estimate the ACME and ADE. The default simulation type [the quasi-Bayesian Monte Carlo method based on normal approximation (62)] was used, with White's heteroskedasticity-consistent estimator for the covariance matrix from the sandwich package [vcovHC; (63)] by setting the robustSE argument to TRUE.

## Results

### Generation of Factor Scores and Assessment of Reliability

The internal consistency (Cronbach's alpha) of the five questionnaire factors in the current sample ranged from 0.71 to 0.78 (Fearfulness 0.770, Aggression toward people 0.774, Activity/Excitability 0.758, Responsiveness to training 0.714, and Aggression toward animals 0.729). This confirmed that the translation of the questionnaire from English to Hungarian, and the modification of the rating scale (from a 7 point to a 5-point Likert scale) did not cause marked changes in the factors' structure. The Cronbach's alpha values from the original study ranged from 0.73 to 0.84 (Fearfulness 0.838, Aggression toward People 0.742, Activity/Excitability 0.728, Responsiveness to Training 0.771, and Aggression toward Animals 0.748).

### Descriptive Information of the Canine Personality Factors

The means, standard deviations, ranges, minimum scores, maximum scores and percentiles for each of the personality factors are shown in Table 2. The Fearfulness and the Aggression toward people factors were positively skewed, with half of the dogs scoring between 1.83 and 2.75 for Fearfulness, and between 1.17 and 2.17 for Aggressiveness toward people. Activity/Excitability and Responsiveness to Training were the most negatively skewed of the factors, with half the dogs scoring between 3.08 and 4.00 for Activity/Excitability, and 3.00 and 4.17 for Responsiveness to Training. At least one dog obtained the maximum score possible on each of the five factors, apart from for Fearfulness. The largest range of scores was obtained for the Responsiveness to training and the Aggression toward people factors while the Fearfulness factor had the smallest range.

**Table 2:** Number of subjects, mean, standard deviation, minimum, maximum, range, and quartiles of the Dog Personality Questionnaire factor scores.

	**DPQ Factors**				
	**Fearfulness**	**Aggression toward People**	**Activity/Excitability**	**Responsiveness to Training**	**Aggression toward Animals**
*N*	1,172	1,184	1,158	1,185	1,173
Mean (%)	2.32	1.80	3.51	3.61	2.69
Std. deviation	0.68	0.78	0.63	0.82	0.77
Minimum (%)	1.00	1.00	1.08	1.00	1.00
Maximum (%)	4.33	5.00	5.00	5.00	5.00
Range	3.33	4.00	3.92	4.00	4.00
25% Percentile	1.83	1.17	3.08	3.00	2.11
50% Percentile	2.25	1.50	3.58	3.67	2.67
75% Percentile	2.75	2.17	4.00	4.17	3.22

### Linear Models: Main Effect of Age

**Figure 1:**
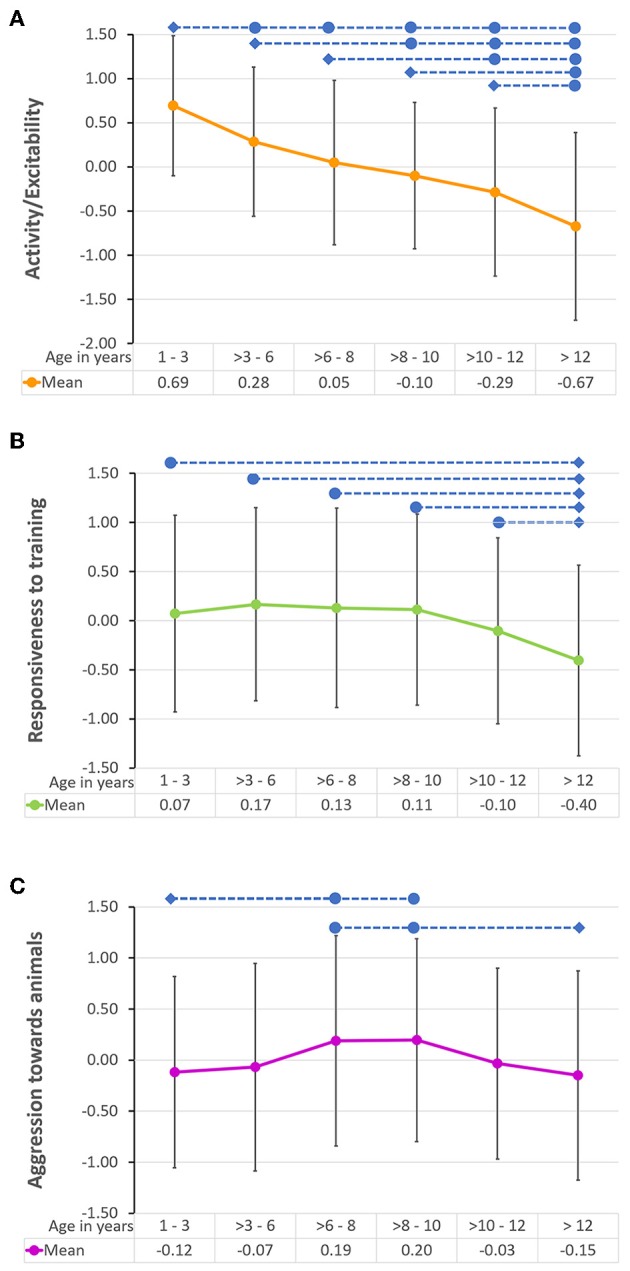
Mean *Z* score (and standard deviation) of the Dog Personality Questionnaire (22) factors **(A)** Activity/Excitability, **(B)** Responsiveness to training, and **(C)** Aggression toward animals, in the six different dog age groups. The blue diamonds indicate the reference age group, and the blue circles and dotted lines represent significant differences between age groups *p* < 0.05 (after correction for multiple comparisons) in the pairwise *post-hoc* analysis. The mean *Z* score for each age group is presented under each graph.

Linear models were run to examine the effect of dog age group on the five DPQ factors. Results revealed a significant effect of age group on Activity/Excitability, which explained 18% of the variance, Responsiveness to training, with 4% variance explained, and Aggressiveness to animals, at only 2% variance explained. Fearfulness and Aggressiveness toward humans had no relationship with dog age (*F* = 1.35, *P* = 0.443; *F* = 0.88, *P* = 0.493, respectively). Activity/Excitability showed a strong negative linear relationship with age, all dog age groups differed significantly from age group 1 (1–3 year olds). Responsiveness to training was highest in 3–6 year olds, and there was a tendency for Responsiveness to training to decrease from age 10, however, only dogs aged above 12 years (age group 6) had significantly lower scores than dogs aged 1–3 years. Aggressiveness toward animals showed a quadratic distribution with age. Dogs aged between 6 and 10 years (age groups 3 and 4) had significantly higher scores than dogs aged 1–3 years (Table S5 and Figures 1A–C below).

Regarding the magnitude of age-related change in personality, Table 3 reports how much each of the three personality traits changed across the different age periods (mean-level changes). *Z* scores are presented for ease of interpretation. The greatest mean-level change (decrease) in Activity/excitability was found in middle age in comparison to early adulthood, followed by the change between late senior and geriatric. Responsiveness to training showed the greatest mean level change between late senior and geriatric, with a significant drop in score. Finally, although there were no significant differences between the sequential age groups in Aggression toward animals, the greatest mean level change (increase) occurred between middle age and late adulthood.

**Table 3:** Results of the linear models of the three PCA factors of the DPQ where a significant relationship with age group was found.

**DPQ Factor**		**Early adult (>1–3 years)**	**Middle age (>3–6 years)**	**Late adult (>6–8 years)**	**Senior (>8–10 years)**	**Late senior (>10–12 years)**	**Geriatric (>12 years)**	**Life-long change**
Activity	*N*	178	241	182	192	162	201	
	Mean	0.692	0.285	0.048	−0.101	−0.286	−0.672	–
	SD	0.794	0.845	0.933	0.829	0.953	1.063	–
	M2–M1		– 0.407	−0.237	−0.149	−0.185	−0.386	−1.364
	T		– 4.826	−2.659	−1.725	−1.776	−3.763	–
	P		**<0.001**	0.084	0.515	0.481	**0.003**	–
Responsiveness to training	*N*	180	241	182	192	162	201	–
	Mean	0.072	0.166	0.129	0.113	−0.104	−0.405	–
	SD	0.999	0.983	1.015	0.972	0.945	0.971	–
	M2–M1		0.094	−0.036	−0.017	−0.217	−0.300	−0.477
	T		0.983	−0.368	−0.205	−2.150	−2.884	–
	P		0.923	0.999	1.000	0.262	**0.046**	–
Aggression toward animals	*N*	177	244	184	198	166	202	–
	Mean	-0.117	-0.069	0.189	0.195	−0.034	−0.150	–
	SD	0.936	1.016	1.030	0.992	0.934	1.024	–
	M2–M1		0.049	0.258	0.006	−0.229	−0.116	−0.032
	T		0.376	2.728	0.060	−2.093	−1.284	–
	P		0.999	0.070	1.000	0.291	0.793	–

*Here only the sequential relationships are presented, i.e., middle age in comparison to early adult, late adult in comparison to middle age etc. N, Number of subjects; Mean, Mean z score; SD, Standard deviation; M2–M1, Difference between the preceding and current age group z score means; T, T value; and P, Adjusted p values (Turkey contrasts for multiple comparisons of means). Bold type indicates p < 0.05*.

Finally, we also examined how age influenced each individual Facet of the DPQ, and since none were normally distributed, and transformations did not result in normalized residuals, we performed non-parametric Kruskal Wallis Tests to look for differences between age groups. Significant age effects were found in the following 11 facets (out of 15): Fear of people, Non-social fear, Excitability, Playfulness, Active engagement, Companionability, Trainability, Controllability, Aggression toward animals, Prey drive and Dominance over dogs (all *p* < 0.003; Figures S1A–K). Fear of people peaked in dogs aged three to 6 years and was lowest in dogs aged over 10 years. Non-social fear increased with age, with dogs aged over 12 years showing the highest levels, and dogs aged under 3 years the lowest levels. Excitability, Playfulness, and Active engagement all showed a significant linear decrease with age (highest scores in dogs aged one to three, and the lowest in dogs aged over 12 years). Trainability scores remained high until declining from 10 years onwards. Controllability showed a quadratic distribution, peaking in three to 6 year olds, and was lowest in dogs aged over 12 years. Aggression toward dogs was lowest in the youngest age group, and highest in the oldest. Conversely, prey drive was highest in the youngest age group and lowest in the oldest. Finally, Dominance over dogs showed a quadratic distribution and peaked in dogs aged 8 to 10. Please refer to the Supplementary Materials for pairwise comparison of each age group, along with test statistic, standard error, significance level, and adjusted significance level for multiple comparisons.

### Linear Models: Main Effects of All Explanatory Variables

Linear models were run to examine the effects of the explanatory variables and age group on the five DPQ factors. Since most of the demographic and explanatory variables were previously shown to differ according to the age group of the dog (56), in order to control for these age differences, all of the variables were left in the models (i.e., the models were not reduced), except five variables which were not significant in the models and did not show age differences (specifically, owner experience, other dogs in the household, people in household, child, and time spent alone). We found that dog age group was still significantly associated with the Activity/Excitability trait, and also for the Aggressiveness toward animals. However, the significant main effect in the linear model of Responsiveness to training disappeared after FDR correction (*p* = 0.475, Table 4).

**Table 4:** Results of the linear models on the five PCA factors of the DPQ.

**Source**	**df**	**Fearfulness (*****N*** **=** **1,170)**				**Aggression toward people (*****N*** **=** **1,182)**				**Activity excitability (*****N*** **=** **1,156)**				**Responsiveness to training (*****N*** **=** **1,183)**				**Aggression toward animals (*****N*** **=** **1,171)**			
		***F***	***P***	**FDR**	**Partial eta2**	***F***	***P***	**FDR**	**Partial eta2**	***F***	***P***	**FDR**	**Partial eta2**	***F***	***P***	**FDR**	**Partial eta2**	***F***	***P***	**FDR**	**Partial eta2**
**Corrected Model**		**6.542**	**<0.001**		**0.230**	**3.327**	**<0.001**		**0.131**	**10.800**	**<0.001**		**0.333**	**18.830**	**<0.001**		**0.459**	**2.884**	**<0.001**		**0.116**
**Age group**	5	2.690	0.020	0.066	0.012	0.897	0.482	0.605	0.004	**18.153**	**0.000**	**0.000**	**0.076**	1.077	0.372	0.475	0.005	**4.113**	**0.001**	**0.012**	**0.018**
Height (in cm)	1	0.194	0.659	0.288	0.000	0.578	0.447	0.605	0.001	0.137	0.711	0.779	0.000	2.094	0.148	0.340	0.002	1.266	0.261	0.375	0.001
**Weight (in kg)**	1	**29.058**	**0.000**	**0.000**	**0.025**	2.569	0.109	0.358	0.002	0.938	0.333	0.418	0.001	**7.039**	**0.008**	**0.031**	**0.006**	1.512	0.219	0.336	0.001
**Breed**	1	**16.562**	**0.000**	**0.001**	**0.015**	**10.112**	**0.002**	**0.012**	**0.009**	**3.387**	**0.066**	**0.169**	**0.003**	0.238	0.626	0.719	0.000	5.841	0.016	0.061	0.005
**Sex**	1	**9.013**	**0.003**	**0.010**	**0.008**	**14.386**	**0.000**	**0.000**	**0.013**	3.806	0.051	0.147	0.003	**10.334**	**0.001**	**0.008**	**0.009**	2.739	0.098	0.188	0.002
Neuter status	1	3.173	0.075	0.173	0.003	1.097	0.295	0.590	0.001	0.000	0.985	0.985	0.000	0.993	0.319	0.459	0.001	3.065	0.080	0.178	0.003
**Sensory problems**	1	2.205	0.138	0.693	0.002	1.729	0.189	0.543	0.002	**6.526**	**0.011**	**0.042**	**0.006**	1.516	0.218	0.380	0.001	5.967	0.015	0.061	0.005
**Off-leash activity**	4	1.510	0.197	0.377	0.005	0.718	0.580	0.642	0.003	2.039	0.087	0.200	0.007	**3.872**	**0.004**	**0.023**	**0.014**	1.866	0.114	0.202	0.007
**Body Condition Score**	2	0.411	0.663	0.693	0.001	0.693	0.500	0.605	0.001	**5.427**	**0.005**	**0.029**	**0.010**	0.385	0.681	0.719	0.001	1.851	0.158	0.260	0.003
**Food**	4	0.930	0.446	0.603	0.003	1.374	0.241	0.564	0.005	1.356	0.247	0.379	0.005	2.959	0.019	0.055	0.010	**3.443**	**0.008**	**0.046**	**0.012**
Vitamins	3	0.769	0.512	0.654	0.002	0.190	0.903	0.903	0.001	1.938	0.122	0.236	0.005	1.428	0.233	0.380	0.004	0.739	0.529	0.676	0.002
**Trauma**	1	**40.734**	**0.000**	**0.000**	**0.035**	**22.142**	**0.000**	**0.000**	**0.019**	1.619	0.204	0.335	0.001	0.805	0.370	0.475	0.001	**8.502**	**0.004**	**0.031**	**0.008**
Health problems	4	2.719	0.029	0.082	0.010	0.356	0.840	0.878	0.001	1.225	0.298	0.403	0.004	1.635	0.163	0.341	0.006	2.051	0.085	0.178	0.007
Medication	1	0.299	0.585	0.684	0.000	0.297	0.586	0.642	0.000	1.724	0.189	0.334	0.002	0.161	0.688	0.719	0.000	0.063	0.802	0.922	0.000
Owner age	3	1.007	0.389	0.559	0.003	1.030	0.379	0.604	0.003	0.939	0.421	0.484	0.003	1.500	0.213	0.380	0.004	0.106	0.957	0.957	0.000
**Age of dog when arrived**	3	1.163	0.323	0.495	0.003	3.104	0.026	0.120	0.008	**14.223**	**0.000**	**0.000**	**0.037**	0.719	0.541	0.655	0.002	3.224	0.022	0.072	0.009
**Get dog**	2	1.163	0.313	0.495	0.002	1.180	0.308	0.590	0.002	**4.945**	**0.007**	**0.032**	**0.009**	0.029	0.972	0.972	0.000	0.069	0.933	0.957	0.000
Where dog is kept	2	1.448	0.236	0.417	0.003	2.503	0.082	0.314	0.004	1.065	0.345	0.418	0.002	1.394	0.248	0.380	0.002	2.548	0.079	0.178	0.005
**Dog obedience tasks**	3	**5.895**	**0.001**	**0.003**	**0.016**	**12.157**	**0.000**	**0.000**	**0.031**	**12.738**	**0.000**	**0.000**	**0.033**	**83.808**	**0.000**	**0.000**	**0.182**	**7.275**	**0.000**	**0.000**	**0.019**
**Play**	3	0.091	0.965	0.965	0.000	0.940	0.420	0.604	0.002	1.931	0.123	0.236	0.005	**4.207**	**0.006**	**0.028**	**0.011**	0.248	0.863	0.945	0.001
**Commands**	2	0.520	0.595	0.684	0.001	0.869	0.420	0.604	0.002	3.388	0.034	0.112	0.006	**19.961**	**0.000**	**0.000**	**0.034**	0.801	0.449	0.607	0.001
**Dog training activities**	2	**7.052**	**0.001**	**0.004**	**0.012**	0.925	0.397	0.604	0.002	1.228	0.293	0.403	0.002	4.064	0.017	0.055	0.007	0.408	0.665	0.805	0.001
Dog behavior changed	1	4.191	0.041	0.105	0.004	1.350	0.245	0.564	0.001	0.068	0.794	0.830	0.000	5.025	0.025	0.064	0.004	2.970	0.085	0.178	0.003

*Owner experience, Other dogs in the household, People in household, Child, and Time spent alone were removed from the model, as all p values were non-significant, and none of these variables differed among the age groups. All other non-significant effects remained in the model in order to control for age effects on the explanatory variables. P values were corrected for multiple comparisons using the false discovery rate procedure (FDR). Significant predictors are highlighted in bold and colored (p ≤ 0.05)*.

In addition to dog age group, we found numerous associations between the dog and owner demographics and other explanatory variables and the personality traits (Table 4). Results for the Fearfulness factor revealed significant effects of previous trauma (3.5% variance explained), weight in kg (2.5%), breed (1.5%), number of dog obedience tasks known (1.6%), number of dog training activities currently participating in (1.2%), and sex (0.8%), which including the remaining 17 variables explained a total of 23.0% of the variance. Results from all the models can be found in Table S5) but are briefly summarized here. Previously we established that dogs that have experienced one or more traumatic events (such as spent time at a shelter, changed owner, suffered traumatic injury/prolonged disease/surgery, were lost for a time, or who experienced a change in family structure), were more likely to be currently suffering from health and/or sensory problems (56). In the current study, dogs that had previously experienced a traumatic event were scored higher in Fearfulness than dogs that had not, and dogs with a higher weight in kg were scored lower in Fearfulness than lighter dogs. Males and pure breeds scored lower in Fearfulness than females and mixed breeds. Finally, dogs that could perform three or more types of obedience tasks, and/or participated in four or more dog training activities had lower Fearfulness scores than dogs that could perform maximum one task, or one dog training activity.

The 23 explanatory variables together accounted for 13.1% of the total variance of the Aggression toward people score. Four variables had significant associations after correction for FDR: Purebreds were rated to be less aggressive than mixed breeds, and males had higher Aggression toward people than females. Dogs that had experienced trauma had higher Aggression toward people, and dogs that knew three or more dog obedience tasks had lower aggression than dogs that knew maximum one task. From the four variables, three had a higher than 1% effect size: number of dog obedience tasks known (3.1%), previous trauma (1.9%), and sex (1.3%). After excluding 16 outliers and rerunning the model, all results that were significant according to FDR Benjamini-Hochberg method remained significant.

Results for the Activity/Excitability factor revealed significant effects of age group (7.6% variance explained), age of dog when arrived (3.7%), dog obedience tasks (3.3%), body condition score (1%), where the dog was obtained from (get dog) (0.9%), and sensory problems (0.6%), which including the remaining 22 variables explained a total of 33.3% of the variance. As dogs age increased Activity/Excitability decreased, and dogs that arrived in the household aged 7 weeks or older received lower Activity/Excitability scores than dogs that were obtained from under 7 weeks. Dogs that knew two or more types of dog obedience tasks were rated as higher in Activity/Excitability than dogs that knew maximum one task. Overweight dogs and dogs with sensory problems were scored lower on Activity/Excitability levels than dogs in a normal weight range, with no sensory problems. Finally, dogs that were born in the household or bought by the owner from a breeder, had lower Activity/Excitability scores than dogs that were found as a stray, or obtained from the shelter. After excluding three outliers and rerunning the model, all results that were significant according to FDR Benjamini-Hochberg method remained significant.

The 23 explanatory variables together accounted for 45.9% of the total variance of the Responsiveness to training score. Results revealed that as a consequence of successfully uncovering one or more mediator variables, we could no longer detect age group differences in this personality trait. Six explanatory variables had significant associations after correction for FDR: Dogs that knew two or more dog obedience tasks had higher Responsiveness to training than dogs that knew maximum one task, and dogs that knew 11 or more commands were rated higher in Responsiveness to training than dogs that knew 10 or fewer. Dogs that engaged in more than 1 h of off leash activity had greater Responsiveness to training scores, than dogs that received <30 min. Owners who engaged in play or other activities with their dog for more than 1 h per day gave their dog higher scores in Responsiveness to training than owner who spent <30 min. Male dogs were rated to be less Responsive to training than females, and heavier dogs had higher Responsiveness to training scores than lighter dogs. From the six variables, four had a higher than 1% effect size: dog obedience tasks (18.2%), number of commands known (3.4%), off leash activity (1.4%), and time spent in play (1.1%). After excluding one outlier and rerunning the model, all results that were significant according to FDR Benjamini-Hochberg method, remained significant.

Results for the Aggressiveness toward animals factor revealed significant effects of dog obedience tasks (1.9% variance explained), age group (1.8%), diet (food) (1.2%), and previous trauma (0.8%), which including the remaining 19 variables explained a total of 11.6% of the variance. As described previously, Aggressiveness toward animals showed a quadratic distribution with age; dogs aged between 6 and 10 years had significantly higher scores than dogs aged 1–3 years. Dogs that could carry out four or more dog obedience tasks had lower Aggressiveness toward animals than dogs that could perform maximum one task. Dogs fed cooked food and/or raw meat, as well as dogs fed a mixture of foods had higher owner reported Aggressiveness toward animals than dogs fed a diet of tinned food, or tinned and dry food mixed. Finally, dogs that had previously experienced trauma scored higher in Aggressiveness toward animals.

### Mediation Analysis: Responsiveness to Training

**Figure 2:**
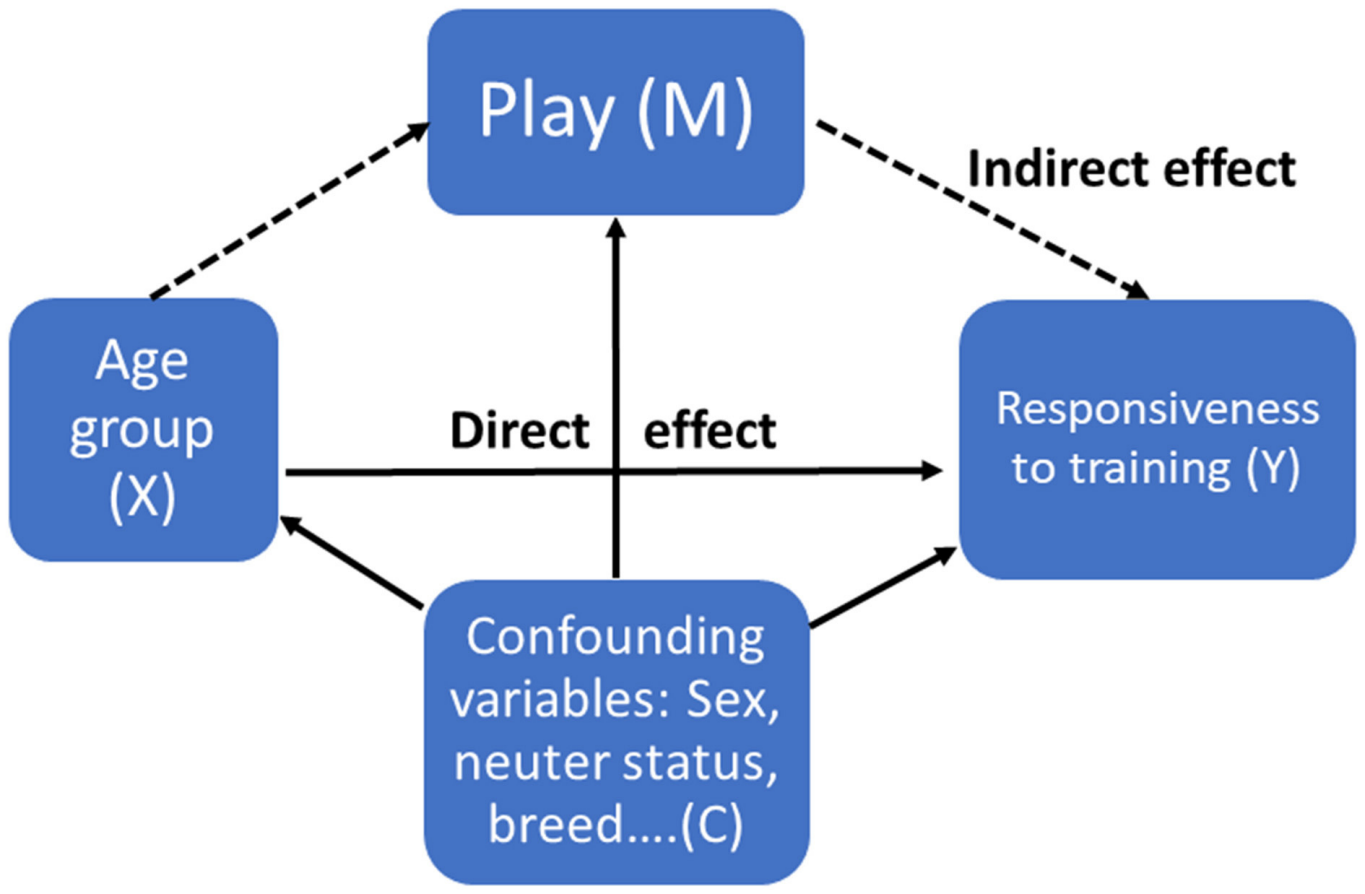
Proposed relationship between dog age group (X), the explanatory variable play [mediator (M): “on an average day, how much time do you or other people spend together with your dog in different activities?” (Playing, walking, training)], and the Dog Personality Questionnaire factor Responsiveness to training (Y). The dotted line represents indirect effects and the solid line direct effects. Confounding variables includes all demographic and other explanatory variables retained in the final model.

When modeled separately, some explanatory variables and Responsiveness to training both showed an effect of age group, therefore it is possible that a mediation may take place between this factor and one or more of the variables. To follow up on this possibility we looked for potential mediator variables by examining which of the significant explanatory variables in the model had the strongest relationship with dog age (including off-leash activity, dog obedience tasks, play, and commands). Results revealed that the explanatory variable play had the greatest differences between the age groups [χ^(15)^ = 61.282, *p* < 0.001]. Please refer to Wallis et al. (56) for results of the age analysis. The oldest age group had the lowest amount of time playing/interacting with the owner in comparison to younger age groups. Therefore, we were interested in finding out whether some owners of older dogs maintain or even increase the amount of time they spend playing/interacting with their dog in comparison to other owners, who decrease the amount of time. A high level of interaction with the owner could indirectly result in maintaining the dogs' levels of Responsiveness to training in old age, thereby mediating the effect of age. Therefore, dog age may not be the real reason that Responsiveness to training decreases in the oldest age groups (in the age only model). We hypothesized that as dog age increases, time in play with the owner decreases in some dogs and then low play levels decreases Responsiveness to training: Age group (X) → Play [mediator (M)] → Responsiveness to training [response variable (Y)] (Figure 2). The mediation analysis effectively tests (1) the influence of different amounts of time in play with the owner in dogs of a similar age (indirect effect), and (2) the influence of age group in dogs with similar amounts of time in play with the owner (direct effect), on the response variable, Responsiveness to training.

We estimated the average causal mediation effect of play, by first fitting the mediator model, where the categorical variable play [mediator (M)], is modeled as a function of dog age group (X) and confounding variables [(C) weight, height, breed, sex, neuter status, etc. (see Table 3 for full list of final model variables)]. Next, we modeled the outcome variable Responsiveness to training, including the mediator (play), age group, and the same set of confounding variables as those used in the mediator model. We used proportional odds logistic regression for the mediator, and linear regression for the outcome model. When comparing the age group with the highest Responsiveness to training, with that of the lowest [age group two and six (adult vs. old dogs)], results from the mediation analysis indicated that there was a significant average causal mediation effect (ACME or indirect effect), but the average direct effect and the total effect were not significant. Results from the mediation analysis posits a partial mediation and the average proportion mediated was 18%. The results suggest that the mediating variable play accounts for a significant part (but not all) of the relationship between dog age and Responsiveness to training. Therefore, the difference in the play variable (mediator) in older dogs in part is responsible for the lower Responsiveness to training personality trait score. The absence of a significant total effect can be explained by the presence of several mediating paths that may cancel each other out. Please refer to the Supplementary Materials for results of the mediation analysis.

## Discussion

Previously we established that aging influences multiple dog and owner demographics (56), therefore the next step was to examine whether the personality of the dog as measured by the Dog Personality Questionnaire (22), also fluctuates with age. In this study, we demonstrated that according to their age group, dogs do indeed differ in their mean personality trait levels. Younger dogs had higher Activity/Excitability levels than older dogs, while older dogs had lower Responsiveness to training. Aggressiveness toward animals showed a quadratic trajectory with age and peaked between 6–10 years. The greatest magnitude of age-related change in personality occurred between the age groups late senior and geriatric, in the Activity/excitability and Responsiveness to training traits. This finding is likely to be caused by compensatory behavioral changes to biological aging (64), and also could be influenced by the owner's attitude to their aging dog [geriatric dogs received less activity/interaction/training with the owner than other age groups (56)]. Previous studies have shown that in adult dogs, cognitive changes generally occur after middle age in parallel with a decline in sensory and motor systems (38, 65). In the current study, 65% of geriatric dogs had sensory issues, in comparison to 23% of late seniors; which may help explain why we observed the greatest magnitude of age-related change in personality in the geriatric age group. There was also evidence for change between early adulthood, middle age and late adulthood. In humans, the greatest mean level changes in personality occur during early adulthood (1) and then the rate of change slows down. Therefore, it seems likely that the greatest period of personality development in dogs would occur from puppyhood to early adulthood. Unfortunately, dogs aged under 1 year were excluded from the sample, so we were not able to examine the magnitude of age-related change in personality for this period.

Previous studies have observed that multiple environmental factors can mask or even enhance differences in biological factors such as age, sex, neuter status, and breed (33, 36). Therefore, in a second set of models we additionally controlled for demographic and other explanatory variables. Results revealed that as a consequence of successfully uncovering one or more mediator variables, we could no longer detect age group differences in the personality trait Responsiveness to training. The most important factors that influenced personality traits (that had a higher than 1% variance explained) were age, weight, breed (pure breed or mixed breed), sex, off-leash activity, diet, previous trauma, age of the dog when it first arrived in the household, number of dog obedience tasks the dog could perform, time spent in play/interacting with owner, number of known commands, and current dog training activities.

Interestingly, although we did not find an effect of age in the main Fearfulness factor of the DPQ, we did find age differences in the facets Fear of people and Non-social fear (see Supplementary Materials). Fear of people peaked in dogs aged 3–6 years and was lowest in dogs aged over 10 years. Non-social fear increased with age, with dogs aged over 12 years showing the highest levels, perhaps due to a decline in environmental stimulation opportunities and to sensory dysfunction. Previously we detected a trend for the oldest age group to be shorter in height than the other age groups [41 cm in comparison to 43–45 cm; Kruskal Wallis test = 11.37, *p* = 0.055 (56)]. Which could indicate that the higher non-social fear score in this group was caused by generally higher fearfulness in smaller dogs. However, there were no differences between the groups in weight in kg (56). Given the small differences between the age groups it seems unlikely that the higher non-social fearfulness in the oldest age group was due to differences in height. Since 65% of geriatric dogs had sensory issues, the higher non-social fear is much more likely to be due to sensory dysfunction in this group. Non-social fear was characterized by higher anxiety, diffidence, and difficulties to adapt to new situations and environments. Previous studies have reported increased anxiety in aged dogs, including increased neuroticism (40), fear of handling, fear of noises (14, 41, 66) and human and object fear (34). Since older dogs are more likely to suffer from painful conditions (e.g., osteoarthritis) (67), when not medicated, pain can cause changes in behavior such as increased anxiety and noise sensitivity (68). Unfortunately, the DPQ does not include questions about noise fear, and so we were not able to examine its relationship with age. None the less, increased fear responses in older dogs is particularly relevant for the senior dog-owner bond, as fearfulness and fear-related behavior problems result in an increase in the perceived cost of the dog owner relationship (69), which can ultimately lead to the relinquishment of the dog (70).

Studies have also shown increased anxiety-like behavior in aged mice and rats, and in humans (71–74). Increases in anxiety is one of the symptoms of Alzheimer's disease (75) and also Canine Cognitive Dysfunction (CCD) (76–78). Aged dogs are more likely to suffer from CCD and signs of increased anxiety include the development of phobias, separation anxiety, night waking and vocalizations, as well as disorientation and changes in social interactions, such as altered relationships with family members, family pets and unfamiliar pets (77). Increased anxiety can also be caused by medical conditions such as sensory dysfunction, metabolic disorders, and pain (67). In older humans, anxiety is often generalized, but in rats and dogs, individual increases in social and non-social anxiety can be expressed separately (78, 79). In the current study, only non-social anxiety increased with age, whilst social anxiety showed a different trajectory. This finding could be explained by the fact that researchers have suggested that the same genomic region affected by structural variants in human Williams-Beuren syndrome (WBS) is associated with hyper-sociability found in most domestic dogs (80). Note that sociability toward unfamiliar people varies a great deal, in some breeds hyper-sociability is favored, and in others it is not a desirable trait. WBS is a multisystem congenital disorder characterized by hypersocial behavior and often heightened non-social anxiety (81). Since many dogs are hyper-social (in comparison to their closest living relative the wolf), a small increase in non-social anxiety may be a normal consequence of aging in dogs, however, large changes in non-social and social anxiety may be an indication of pathological aging, once other related medical conditions have been ruled out. Another explanation for the differing cross-sectional trajectories of social and non-social fear may be due to the fact that these processes involve differing neurological regions and neurotransmitters, based on evidence from rat and human studies (82, 83).

Unsurprisingly, the factor Activity/Excitability, which contains the facets Excitability, Playfulness, Active Engagement and Companionability, showed a strong decline with age in the current cross-sectional study. Several studies have reported decreases in activity levels with age in dogs within the home environment (84, 85). This factor also includes questions regarding sociability (companionability, or time spent interacting with humans), and playfulness, which are also reported to decline with age in dogs (39, 84, 86). Using the same questionnaire Chopik and Weaver (43) found a similar decline of the factor Activity/Excitability with age. Utilizing a different personality questionnaire based on a Human Personality Inventory, Kubinyi et al. (36) found that older dogs were calmer, less social and less bold than younger dogs [see also Starling et al. (35) for decrease in boldness with age] which also points to a reduction in activity/excitability and sociability with age.

In the current study, Responsiveness to training also declined with age, after peaking in the 3–6-year-old dogs. Chopik and Weaver (43) reported a peak in Responsiveness to training in dogs aged 7 years, and no decline with age. This can be explained by the fact that their sample was skewed toward younger dogs and contained fewer senior and geriatric dogs in comparison to the current study. By measuring selective attention, sensorimotor control and trainability using a clicker training for eye contact test in a large sample of pet Border collies aged from 6 months to 14 years, Wallis et al. (38) similarly to our results, found that the dogs' performance peaked in the 3–6 year olds. Kubinyi et al. (36) and Turcsán et al. (33) also found a reduction in Trainability in older dogs, especially those that did not take part in any training activities and whose owners spent < 1 h active with them daily.

Finally, Aggressiveness toward animals increased with age up to 10 years, but then declined. Several studies have reported increases in intraspecific aggression in dogs with age (25, 39, 87). Similarly to the current study, Chopik and Weaver (43) documented a peak in the factor Aggressiveness toward animals at 7–8 years old, and a decline thereafter. However, this factor also contains the facets Prey drive and Dominance over dogs. In the current study, results indicated that the oldest age group had the lowest scores in both facets. Which explains why older dogs overall had lower Aggressiveness toward animals, despite the fact that the facet Aggressiveness toward dogs was highest in the oldest age group. This information is particularly relevant for owners of aged dogs living in multi-dog households as their management and housing could be affected. If increased aggressiveness toward dogs within the same household is observed, pain issues should be ruled out first, and preventative measures be implemented such as providing separate sleeping areas and feeding locations, in order to minimize conflicts. Prey drive and Dominance over dogs may be reduced in the oldest age group due to falling activity levels or age-related frailty and/or increased pain levels, and a corresponding decline in walks and opportunities to meet unfamiliar conspecifics and/or other animals.

Our next aim was to investigate if the behavior differences between the dog age groups remained significant after controlling for any differences in the demographic and dog keeping factors, as well as to examine how the demographic and explanatory variables are associated with the behavioral traits. Results revealed that due to the fact that we successfully uncovered one or more mediator variables we could no longer detect age group differences in the personality trait Responsiveness to training. For the Activity/Excitability and Aggression toward animals behavior traits, dog age group remained a significant predictor in the models even after controlling for the measured explanatory variables. This suggests that these age-related behavioral differences (i.e., lower Activity/Excitability in older dogs, and higher Aggression toward animals in dogs aged six to ten), remained even after taking into account other demographic variables known to change with age [investigated in Wallis et al. (56)].

When we explored the relationship between the demographic and other explanatory variables and the behavior traits, we found that 15 out of the 28 variables were significantly associated with at least one behavior trait, after correction for multiple comparisons. Here we will discuss only those variables that accounted for <1% variance explained in the models (including weight, breed, sex, off-leash activity, food (diet), previous trauma, age of dog when arrived, dog obedience tasks, play, commands, and dog training activities).

The explanatory variables weight and breed (mixed or pure bred) were significant in the DPQ factor Fearfulness. Larger, heavier dogs scored lower in Fearfulness than smaller lighter dogs, and mixed breeds were higher in Fearfulness than pure breeds. Our results are supported by previous studies that found that smaller dogs were seen as more anxious, neurotic and fearful in comparison to larger dogs (21, 47, 88), which might help explain why in the current study smaller dogs had lower scores in Responsiveness to training than larger dogs. Mixed breeds were also found to be more fearful than pure breeds (39, 40, 89), which may heighten their tendency to show aggression toward people. Mixed breed dogs and small dogs in comparison to pedigree and large dogs may be subject to different early life experiences, as well as different perceptions from their owners, which could also explain the observed differences found in the current study. The factors Aggression toward animals and Aggression toward people had a low percentage of variance explained in the models and were less influenced by environmental factors; therefore, additional variables not measured in the current study likely contribute to these personality traits. Previous studies suggest that dogs are likely to learn to show aggression only in particular contexts. Experiences that are specific to the individual and the type of training method used by the owner may also influence aggression in dogs (87, 90, 91). Genetic factors that contribute to the DPQ factors Fearfulness, Aggression toward People and Activity/Excitability have been identified (92–98). Our results reflect the importance of genetic factors, as breed status was associated with these factors, albeit only one of them with a higher than one percent variance explained.

Similarly, although we found significant sex effects for three of the five personality traits (and a tendency in Activity/Excitability and Aggression toward animals), only one had a higher than one percent variance explained, Aggression toward people. Regardless of breed, owners rated male dogs as higher in aggression than females. Previous studies have found that male dogs score higher on owner directed aggression (42), biting, growling and possessive behavior (99), and over half the dogs reported to display aggressive behavior toward humans are reproductively intact males (100–102). In the current study, female dogs were rated higher in Fearfulness and Responsiveness to training similarly to previous studies (31, 40, 103–105). However, another study reported no relationship between Responsiveness to training and sex, and an interaction between sex and breed was also reported (44, 106). The current study adds to previous studies findings that there are breed and sex specific differences in behavior, behavioral development and heritability of traits in dogs (25, 26, 29, 44). The lack of personality differences found in dogs of different reproductive status in the current study could be because previous studies have found differential effects of neutering on the behavior of males and females [e.g., (90, 107)]. Therefore, future studies should specifically examine sex—neuter status interactions.

The environmental explanatory variable, off-leash activity showed only one association with the personality traits measured. Dogs that engaged in more than 1 h of off-leash activity had greater Responsiveness to training scores, than dogs that received <30 min. This finding is easily explained by the fact that dogs that have received more training and have a better recall, are likely to be allowed more off-leash time than untrained dogs. Some breeds are more commonly allowed off the lead in public by owners, which indicates that there are breed differences in off-leash activity. Additionally, dogs that are more fearful and show aggression to strangers or other dogs (and therefore might not return to the owner when called), are less likely to be allowed off-leash. A recent study found that such dogs were also more likely to be overweight, perhaps because their owners do not allow their dogs to exercise outside the house and garden, or restrict their freedom if they do (108).

The dogs' main diet had a significant association with one behavioral trait. Interestingly, dogs fed cooked food and/or raw meat, as well as dogs fed a mixture of foods (including, cooked food, raw, as well as dry and/or canned food), had higher owner reported Aggressiveness toward animals than dogs fed a diet of only dry food, or tinned food, or tinned and dry food mixed. One explanation for this finding could be due to the fact that dogs which are prone to intraspecific aggression that are fed a higher protein diet, show heightened dominance aggression compared to when they are fed a low protein diet, or a diet supplemented with tryptophan (109). Additionally, raw/cooked food could be considered a more valuable resource than canned/kibble and therefore more likely to trigger resource guarding aggression. Alternatively, owners with strong opinions about their dog's diet may also be biased in their perception of their dog's behavior.

Earlier we established that dogs that have previously experienced one or more traumatic events (such as spent time at a shelter, changed owner, suffered traumatic injury/prolonged disease/surgery, were lost for a time, or who experienced a change in family structure), were more likely to be currently suffering from health and/or sensory problems (56). We speculated that exposure to traumatic experiences causes behavioral changes in dogs such as increased fearfulness and aggression to certain stimuli. In the current study, results showed that dogs that were exposed to previous trauma showed higher fearfulness and aggression toward people and animals, than dogs with no such negative experience. The owners of forty two percent of the dogs in our sample indicated that their dogs had experienced trauma, which seems particularly high, however, 28% of the dogs were from a rescue background and 18% were obtained when they were older than 1 year, which could help explain this high percentage. Future studies should examine whether this holds true for other dog populations. One could speculate that mixed breed dogs were more likely to experience trauma, due to the fact that many of them are obtained from shelters. However, since there were also significant breed effects in these personality traits (only a trend in Aggression toward animals), the fact that trauma was still significant, indicates that this effect was present regardless of whether the animal was a purebred or a mixed breed dog.

To date, few studies have examined the effect of previous trauma on behavioral traits in dogs (28). Serpell and Duffy (110) found that particularly frightening or traumatic events that occurred during puppyhood/adolescence were associated with differences in C-BARQ scores for some behaviors displayed at 12 months. Puppies that had been attacked by an unfamiliar dog displayed higher dog-directed fear, and stranger directed aggression. In addition, puppies that had been frightened by a person showed higher levels of stranger directed fear. However, the authors note that it was not possible to determine whether the dogs became more fearful and/or aggressive as a direct result of their experience or if they had a pre-existing disposition toward fearfulness, which resulted in a higher likelihood to become traumatized by aversive encounters. The same argument can be made of our own results. However, in the current study dogs could have suffered the trauma at any point in their life, and therefore, we can speculate that the effects of previous trauma are likely to persist much longer than the 12-month period in Serpell and Duffy's study (110).

Studies in humans have also indicated that extremely adverse life experiences can have a profound effect on personality. Participants who reported an extremely horrifying or frightening event up to 2 years previously, showed increases in neuroticism, decreases in the compliance facet of agreeableness, and decreases in openness to values (111). These changes correspond to increases in fear, anger and frustration (aggression), and decreases in cooperation in interpersonal relationships. These striking similarities in dog and human neurobiological alterations in behavioral disorders further support the claim that the dog represents an interesting natural animal model for human neuropsychiatric diseases (112, 113).

Dogs that arrived in the household aged older than 7 weeks or that were bred by the owner received lower Activity/Excitability scores than dogs that were obtained from under 7 weeks, or that were found, or rescued from a shelter. Our results are in part agreement to those from Kubinyi et al. (36); dogs bred by the owner were described as being calmer, and bolder than dogs acquired later, especially those acquired as adults (note that a sex difference in boldness was found). In general, puppies that are removed earlier from the dam are more likely to exhibit potentially problematic behaviors (114). Increased activity/excitability could also be a product of early separation from the mother. However, we did not observe any other effects of the age of dog when it first arrived in the household, apart from a tendency of dogs that arrived over 1 year of age to have lower aggression toward people and animals, than dogs that arrived at a younger age.

The final four explanatory variables that influenced the dog behavioral traits were dog obedience tasks, amount of time in play or other activities, number of known commands, and dog training activities. Dog obedience tasks was the only demographic variable that had a higher than 1% variance explained in all five of the models. A greater number of dog obedience tasks known by the dog corresponded to lower Aggressiveness toward animals, Fearfulness and Aggression toward people, and higher Activity/Excitability and Responsiveness to training, than dogs that could perform maximum one task. These results suggest that there is a link between the number of obedience tasks known and personality as assessed by the owner in pet dogs. However, this is a correlation and although changes in the number of obedience tasks known may lead to changes in personality, it is also possible that the dogs already had the type of personality that would be amenable to training (lower aggression and fearfulness, and higher Activity/Excitability), which resulted in greater levels of obedience. Regardless of which is cause and which is effect, it is clear that obedience is an important aspect contributing to owner answered dog personality questionnaires, and the dog-owner relationship.

Formal and informal obedience training has been found to reduce aggression, and owners of obedience-trained animals reported fewer behavioral problems (115, 116). However, we should note that owners of fearful or aggressive dogs may start formal training activities, but often do not continue due to high stress levels of the dog and the owner. In these cases, one to one training sessions with qualified trainers are more likely to produce improvements. For example, Casey et al. (87) found that dogs that attended obedience classes had a 1.8 times increased risk of aggression to unfamiliar dogs, perhaps because the owners were seeking assistance with their aggressive dog. A questionnaire study by Bennett and Rohlf (39) found that more obedient dogs (dogs that come when called, and sit and stay on command) were reported to be more friendly, and less aggressive, nervous, and anxious/destructive by their owners. Owners of obedient dogs had greater training engagement and participated in more shared activities with their dog, which could result in a stronger dog-owner bond.

In the current study, dog obedience tasks, time spent in play or other activities and number of commands known explained 22% of the Responsiveness to training factor, providing construct validity for this trait. Additionally, we could show that one explanatory variable, play, partially mediated the relationship between age group and Responsiveness to training. This result is particularly important, as it implies that in older dogs, interventions to increase play and training motivation may alleviate the negative effects of aging on dogs' trainability. Finally, dogs that participated in a higher number of dog training activities had lower Fearfulness scores. Previous studies have also found a link between training and fearfulness. Owners of nervous dogs had lower training engagement (39), and dogs that participated in more training course were rated higher in calmness (36).

The fact that the dogs' level of training (or education) was found to have a stronger influence on owner perceived dog personality than breed, sex or reproductive status provides evidence that educational experiences have the power to shape dogs' personality development. It is generally accepted that children and puppies' personality is dynamic and dependent on the interaction of genetics, biology, and environmental influences. Such phenotypic plasticity allows individuals to adjust to environmental variation, and helps to explain the high heritability of personality in early childhood (117). However, the idea that a dog's or indeed a person's personality is fixed at adulthood and cannot be changed has been pervasive in society in general. Whilst it is true that as individuals enter adulthood the longitudinal stability of personality increases substantially (117) in humans, personality traits continue to change in response to key life stages and events (2). A recent study has proven that interventions can change self-reported personality traits through volitional means (118). Evidence is emerging of the potential of education interventions in children and adolescents to alter personality traits in order to improve resilience (119). Here we define resilience as “any behavioral or emotional response to a cognitive or social challenge that is positive and beneficial for development” (120). Dog obedience training throughout the lifespan may help to increase resilience in dogs, and thus increase their ability to cope with potentially stressful situations, reducing fear and aggressiveness, and increasing responsiveness to training and sociability. Indeed, the success of behavior modification by owners supervised by qualified dog trainers that use positive reinforcement as a tool to “correct” behavioral problems in dogs is a testament to the power of education in improving motivation and resilience in dogs (121–123).

Our study is among the first that aimed to report mean-level differences in personality traits across the lifespan of pet dogs, and to describe the demographic variables that may contribute to them. However, it is important to note that a major limitation of this study is that it is based on owner reports, which are subjective, and in most cases where associations were found, it was not possible to determine the direction of the cause—effect relationships, or indeed whether a real causal relationship does in fact exist. Additionally, as the owners were contacted through social media the sample may have been subject to selection bias, and as such it is not possible to determine whether the results are generalisable to the population. However, the age related results are similar to Chopik and Weaver's (43) study that used the same questionnaire in populations in the US, the questionnaire showed good reliability, and the questionnaire method has previously been proven to be reliable and valid (22) and (17). Many of the associations found can be used to generate new hypotheses and tests that will help to validate the results. A low amount of variance explained in some of the models was likely due to the fact that we were not able to identify and measure all aspects that can influence dog personality. Other factors such as trait heritability and developmental effects like early socialization, rearing environment and early life experience as well as differing perceptions of owners of different breeds and sizes of dogs could also provide explanations for the observed behavioral differences.

## Conclusion

Some of the predicted relationships between demographic variables and dog personality were found (such as age, breed, and sex effects), however, most were small effects, therefore their biological relevance is questionable. Instead, the amount of shared activities, specifically the number of dog obedience tasks known, and the occurrence of previous trauma proved to be more predictive of how owners viewed their dogs' personality.

## Data Availability Statement

All datasets generated for this study are included in the article/Supplementary Material.

## Ethics Statement

Ethical review and approval was not required for the animal study because we collected the data using an online questionnaire designed to assess the dogs and the owners demographic data and dog personality via owner report. According to the currently operating Hungarian law (1998. Evi XXVIII. Torvenyand dog personality —the Animal Protection Act, 3rd paragraph, 9th point), non-invasive observational data collection on dog demographics and behavior are not considered as animal experiments, and are therefore allowed to be conducted without any special permission from the University Institutional Animal Care and Use Committee (UIACUC). The filling out of the questionnaires was voluntary and anonymous so the study did not violate respondents' privacy. Informed consent was included in the introductory letter of the questionnaires. Written informed consent was obtained from the owners for the participation of their animals in this study.

## Author Contributions

EK and DS: conceived and designed the demographic questionnaire. LW, EK, and DS: analyzed the data, interpreted the results, and revised the paper. LW: wrote the first draft of the paper. EK: provided the funding.

### Conflict of Interest

The authors declare that the research was conducted in the absence of any commercial or financial relationships that could be construed as a potential conflict of interest.
