# Supplementary material for: Cross-Sectional Age Differences in Canine Personality Traits; Influence of Breed, Sex, Previous Trauma, and Dog Obedience Tasks
Source: Front Vet Sci. 2020 Jan 14;6:493. doi: 10.3389/fvets.2019.00493 (PMC6971055; doi:10.3389/fvets.2019.00493)
Supplement: Supplementary file 1 [file Data_Sheet_1.docx]

# *Supplementary Material*

## Demographic variables of dog and owner

### **Table 1**: Description of categorical questions concerning the dogs and their owners (N=1207), and percentage breakdown of the groups. (BCS: Body condition score)

| **Title and description** | **Categorical Variable labels:** | **Count** | **%** |
| --- | --- | --- | --- |
| **Age group** | Group One: 1 - 3 years | 185 | 15.3 |
|  | Group Two: >3 - 6 years | 251 | 20.8 |
|  | Group Three: >6 - 8 years | 191 | 15.8 |
|  | Group Four: >8 - 10 years | 202 | 16.7 |
|  | Group Five: >10 - 12 years | 170 | 14.1 |
|  | Group Six: >12 years | 208 | 17.2 |
| **Neuter status** | Intact | 529 | 43.8 |
|  | Neutered | 678 | 56.2 |
| **Sensory problems** | None | 980 | 81.2 |
|  | Vision and/or hearing | 227 | 18.8 |
| **Off-leash activity:** How long does your dog walk/run around outdoors without a leash on a typical day? | Less than 30 minutes | 164 | 13.6 |
|  | 30 minutes -1 hour | 269 | 22.3 |
|  | >1-3 hours | 367 | 30.4 |
|  | >3-7 hours | 165 | 13.7 |
|  | More than 7 hours | 242 | 20.0 |
| **Body condition score:** What body shape does your dog have? | Thin (BCS 1-2) | 203 | 16.8 |
|  | Normal (BCS 3) | 784 | 65.0 |
|  | Over-weight (BCS 4-5) | 220 | 18.2 |
| **Food:** What food are you currently feeding your dog for its main meal? | Dry food only | 267 | 22.1 |
|  | Tinned &/or dry food | 147 | 12.2 |
|  | Cooked food | 306 | 25.4 |
|  | Mixed | 294 | 24.4 |
|  | Raw meat | 193 | 16.0 |
| **Vitamins**: Do you give your dog vitamins or supplements? | Almost never | 328 | 27.2 |
|  | Rarely | 391 | 32.4 |
|  | Often | 244 | 20.2 |
|  | Regular (daily) | 244 | 20.2 |
| **Trauma:** Has the dog experienced a traumatic event, which could still have an effect on it? | No | 694 | 57.5 |
|  | Yes | 513 | 42.5 |
| **Health Problems:** What kind of health problems does your dog have? | None | 479 | 39.7 |
|  | Tooth problems only | 182 | 15.1 |
|  | Joint problems + tooth problems | 126 | 10.4 |
|  | Joint problems only | 246 | 20.4 |
|  | Other disorders | 174 | 14.4 |
| **Medication**: Is your dog currently taking any medication? | No | 1021 | 84.6 |
|  | Yes | 186 | 15.4 |
| **Owner age** | <29 years | 385 | 31.9 |
|  | 30-39 years | 343 | 28.4 |
|  | 40-49 years | 253 | 21.0 |
|  | >50 years | 226 | 18.7 |
| **Owner experience:** How would you evaluate your experience with dogs? | Dogs are my hobby/profession and/or I am a dog trainer/breeder | 307 | 25.4 |
|  | I have had a dog before | 639 | 52.9 |
|  | I had never had a dog before | 261 | 21.6 |
| **Other dogs in household:** How many other dogs do you have living in your household? (Not including this one). | None | 433 | 35.9 |
|  | One | 474 | 39.3 |
|  | Two or more | 300 | 24.9 |
| **People in household:** How many people are living in the household? | One person (myself) | 141 | 11.7 |
|  | Two people | 503 | 41.7 |
|  | Three people | 271 | 22.4 |
|  | Four or more people | 292 | 24.2 |
| **Child:** Do you have a child/children living in your household? | No | 919 | 76.1 |
|  | Yes | 288 | 23.9 |
| **Age of the dog when arrived:** The age of the dog when it arrived in the Owner’s household | less than 7 weeks | 265 | 22.0 |
|  | 7-12 weeks | 530 | 43.9 |
|  | 3-12 month | 198 | 16.4 |
|  | more than 1 year | 214 | 17.7 |
| **Get dog:** How did you get your dog? | I found it/got it from a shelter | 340 | 28.2 |
|  | It was born at my place/ bought it | 544 | 45.1 |
|  | I got it as a present | 323 | 26.8 |
| **Where dog is kept:** Where do you keep your dog? | House with a garden and/or in a non-fenced area | 149 | 12.3 |
|  | In a fenced garden | 384 | 31.8 |
|  | Urban/Suburban apartment | 674 | 55.8 |
| **Dog obedience tasks:** Which tasks can your dog reliably perform? (E.g., sit, lie down, come, fetch, stay, walk at heel, leave/drop it, watch me etc.). Open question. | maximum a task | 151 | 12.5 |
|  | 2 kinds of tasks | 169 | 14.0 |
|  | 3 kinds of tasks | 228 | 18.9 |
|  | more than 3 kind of tasks | 659 | 54.6 |
| **Play:** On an average day, how much time do you or other people spend together with your dog in different activities? (Play, walking, training) | less than 30 minutes | 122 | 10.1 |
|  | 30 minutes -1 hours | 378 | 31.3 |
|  | >1 -3 hours | 551 | 45.7 |
|  | more than 3 hours | 156 | 12.9 |
| **Commands:** How many commands can your dog execute reliably? | < 10 commands | 540 | 44.7 |
|  | 11 – 30 Commands | 535 | 44.3 |
|  | > 30 Commands | 132 | 10.9 |
| **Dog training activities:** How many activities are you currently doing with your dog? | One activity | 385 | 31.9 |
|  | 2 - 3 activities | 527 | 43.7 |
|  | 4 or more activities | 295 | 24.4 |
| **Time spent alone**: How much time does your dog spend alone on an average working day? | None | 169 | 14.0 |
|  | 1- 2 hours | 276 | 22.9 |
|  | 3- 8 hours | 594 | 49.2 |
|  | More than 8 hours | 168 | 13.9 |
| **Dog behavior changed:** Has your dog’s behavior changed over the last 3 months? | No | 910 | 75.4 |
|  | Yes | 297 | 24.6 |

### **Table 2**: Dog Personality Questionnaire (DPQ)

Here are a number of personality traits and behavioural descriptions that may or may not apply to your dog. Please write a number next to each statement to indicate the extent to which you agree or disagree with that statement. You should rate your dog based on his or her general, overall behaviour: 1 Disagree strongly, 2 Disagree, 3 Neither agree nor disagree, 4 Agree, 5 Agree strongly.

| Item No. | Description |
| --- | --- |
| 1 | Dog is relaxed when greeting people |
| 2 | Dog behaves aggressively toward dogs |
| 3 | Dog is anxious |
| 4 | Dog is lethargic |
| 5 | When off leash, dog comes immediately when called |
| 6 | Dog is shy |
| 7 | Dog behaves aggressively towards unfamiliar people |
| 8 | Dog likes to chase squirrels, birds, or other small animals |
| 9 | Dog gets bored in play quickly |
| 10 | Dog is quick to sneak out through open doors, gates |
| 11 | Dog is confident |
| 12 | Dog is dominant over other dogs |
| 13 | Dog avoids other dogs |
| 14 | Dog works at tasks (eg, getting treats out of a Kong, shredding toys) until entirely finished |
| 15 | Dog is boisterous |
| 16 | Dog behaves fearfully during visits to the veterinarian |
| 17 | Dog enjoys playing with toys |
| 18 | Dog is friendly towards unfamiliar people |
| 19 | Dog is playful with other dogs |
| 20 | Dog seeks companionship from people |
| 21 | Dog behaves submissively (e, rolls over, avoids eye contact, licks lips) when greeting other dogs |
| 22 | Dog adapts easily to new situations and environments |
| 23 | Dog likes to chase bicycles, joggers, and skateboarders |
| 24 | Dog is curious |
| 25 | Dog behaves aggressively in response to perceived threats from people (eg, being cornered, having collar reached for) |
| 26 | Dog is aloof |
| 27 | Dog behaves fearfully towards unfamiliar people |
| 28 | Dog willingly shares toys with other dogs |
| 29 | Dog is slow to respond to corrections |
| 30 | Dog behaves aggressively during visits to the veterinarian |
| 31 | Dog seeks constant activity |
| 32 | Dog leaves food or objects alone when told to do so |
| 33 | Dog retrieves objects (eg, balls, toys, sticks) |
| 34 | Dog is friendly towards other dogs |
| 35 | Dog exhibits fearful behaviors when restrained |
| 36 | Dog aggressively guards coveted items (eg, stolen item, treats, food bowl) |
| 37 | Dog is affectionate |
| 38 | Dog ignores commands |
| 39 | Dog behaves aggressively towards cats |
| 40 | Dog shows aggression when nervous or fearful |
| 41 | Dog tends to be calm |
| 42 | Dog behaves fearfully towards other dogs |
| 43 | Dog is able to focus on a task in a distracting situation (eg, loud or busy places, around other dogs) |
| 44 | Dog behaves fearfully when groomed (eg, nails trimmed, brushed, bathed, ears cleaned) |
| 45 | Dog is assertive or pushy with other dogs (eg, if in a home with other dogs, when greeting) |

### **Table 3**: Dog Personality Questionnaire (DPQ) Factors, Facets, item numbers and reliability (Cronbach’s alpha of the five personality Factors) in the current study.

**
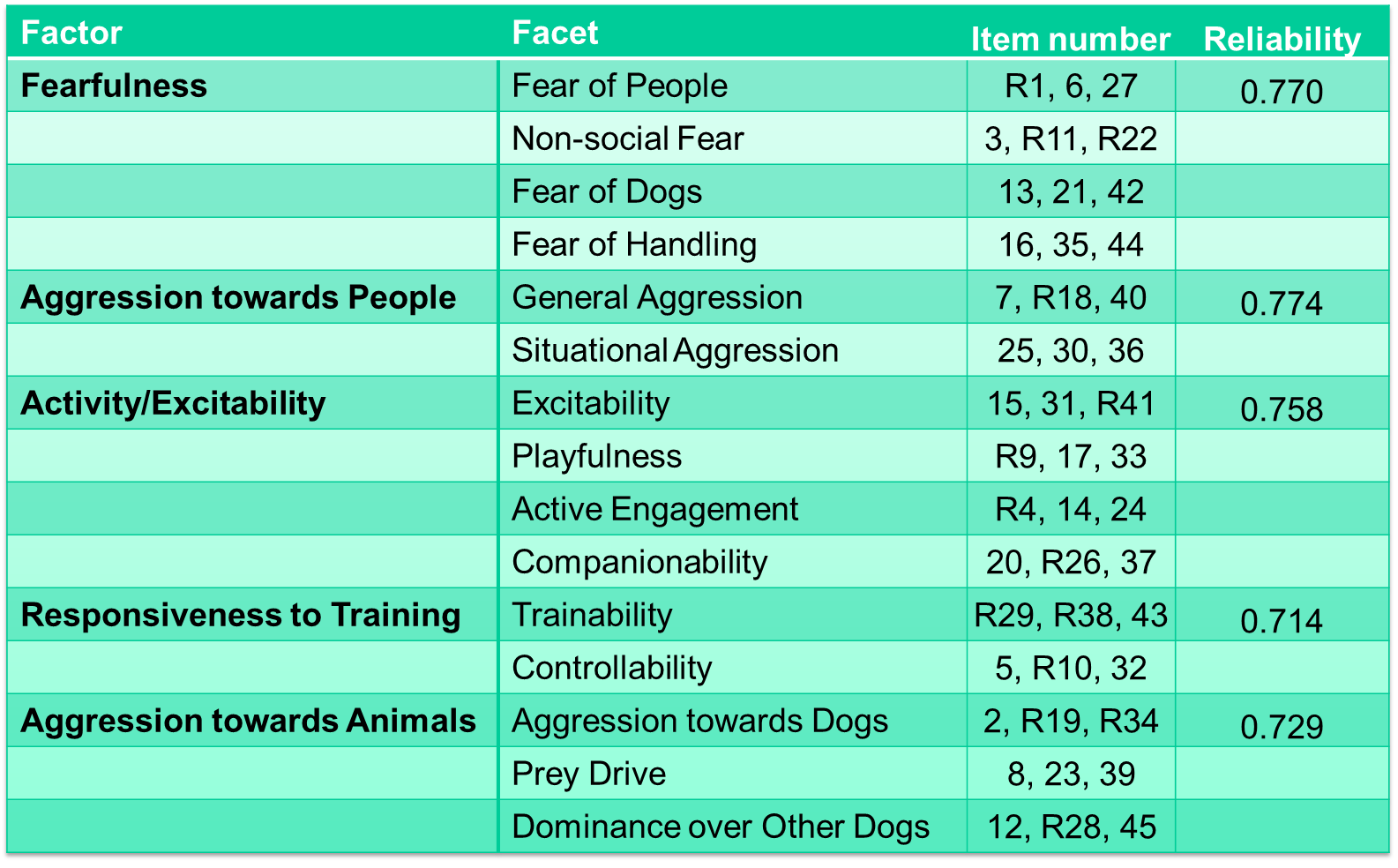
**

## Correlations between the personality subscales

Correlations between the personality factor scores revealed two moderately significant relationships, shown in Table 3 (all sample sizes were 1078). The Aggression towards People factor correlated positively with the Fearfulness factor (r = 0.375, P < 0.001) and Aggression towards Animals (r = 0.488, P < 0.001). There were some other weak correlations, please see Table 4 for details.

### **Table 4: Correlations between the five factors of the DPQ.**

Moderate correlations are highlighted in orange and weak correlations in yellow. *Correlation is significant at the 0.01 level (2-tailed). * Correlation is significant at the 0.05 level (2-tailed).

|  |  | Aggression towards People | Activity/  Excitability | Responsiveness to Training | Aggression towards Animals |
| --- | --- | --- | --- | --- | --- |
| Fearfulness | r | .375^**^ | -.153^**^ | -.223^**^ | .106^*^ |
|  | Sig. (2-tailed) | .000 | .000 | .000 | .000 |
| Aggression towards People | r |  | -.082^*^ | -.203^**^ | .488^**^ |
|  | Sig. (2-tailed) |  | .007 | .000 | .000 |
| Activity/  Excitability | r |  |  | .217^**^ | .078^**^ |
|  | Sig. (2-tailed) |  |  | .000 | .011 |
| Responsiveness to Training | r |  |  |  | -.174^**^ |
|  | Sig. (2-tailed) |  |  |  | .000 |

## Main effect of age on the DPQ factors

### **Table 5: Results of the age group linear models for the three factors of the Dog Personality Questionnaire where a significant effect of age group was found.**

Significant predictors are highlighted in bold (p≤0.05). Adjusted p values are presented and Turkey contrasts for multiple comparisons of means was utilised to examine differences between the age groups.

|  | **Estimate** | **Std. Error** | **T** | | **F value** | | **P** | | **Multiple R**^2^ | **Adjusted R**^2^ |
| --- | --- | --- | --- | --- | --- | --- | --- | --- | --- | --- |
| **Activity Excitability (lambda = 1.59)** | | |  | |  | |  | |  |  |
| Age group |  |  |  | | 51.340 | | **<0.001** | | 0.183 | 0.179 |
| Age group 2: Age group 1 | -0.556 | 0.115 | -4.826 | |  | | **<0.001** | |  |  |
| Age group 3: Age group 1 | -0.860 | 0.123 | -7.002 | |  | | **< 0.001** | |  |  |
| Age group 4: Age group 1 | -1.068 | 0.121 | -8.809 | |  | | **< 0.001** | |  |  |
| Age group 5: Age group 1 | -1.289 | 0.127 | -10.185 | |  | | **< 0.001** | |  |  |
| Age group 6: Age group 1 | -1.752 | 0.120 | -14.606 | |  | | **< 0.001** | |  |  |
| Age group 3: Age group 2 | -0.304 | 0.114 | -2.659 | |  | | 0.084 | |  |  |
| Age group 4: Age group 2 | -0.512 | 0.113 | -4.544 | |  | | **< 0.001** | |  |  |
| Age group 5: Age group 2 | -0.733 | 0.118 | -6.191 | |  | | **< 0.001** | |  |  |
| Age group 6: Age group 2 | -1.196 | 0.111 | -10.745 | |  | | **< 0.001** | |  |  |
| Age group 4: Age group 3 | -0.208 | 0.121 | -1.725 | |  | | 0.515 | |  |  |
| Age group 5: Age group 3 | -0.429 | 0.126 | -3.406 | |  | | **0.009** | |  |  |
| Age group 6: Age group 3 | -0.892 | 0.119 | -7.479 | |  | | **< 0.001** | |  |  |
| Age group 5: Age group 4 | -0.221 | 0.124 | -1.776 | |  | | 0.481 | |  |  |
| Age group 6: Age group 4 | -0.684 | 0.118 | -5.815 | |  | | **< 0.001** | |  |  |
| Age group 6: Age group 5 | -0.463 | 0.123 | -3.763 | |  | | **0.003** | |  |  |
| **Responsiveness to training (lambda = 1.31)** | | |  |  | |  | |  | |  |
| Age group |  |  |  | | 10.220 | | **<0.001** | | 0.042 | 0.038 |
| Age group 2: Age group 1 | 0.114 | 0.116 | 0.983 | |  | | 0.923 | |  |  |
| Age group 3: Age group 1 | 0.072 | 0.123 | 0.584 | |  | | 0.992 | |  |  |
| Age group 4: Age group 1 | 0.047 | 0.121 | 0.390 | |  | | 0.999 | |  |  |
| Age group 5: Age group 1 | -0.219 | 0.127 | -1.726 | |  | | 0.514 | |  |  |
| Age group 6: Age group 1 | -0.573 | 0.120 | -4.766 | |  | | **<0.001** | |  |  |
| Age group 3: Age group 2 | -0.042 | 0.114 | -0.368 | |  | | 0.999 | |  |  |
| Age group 4: Age group 2 | -0.067 | 0.112 | -0.593 | |  | | 0.992 | |  |  |
| Age group 5: Age group 2 | -0.332 | 0.118 | -2.806 | |  | | 0.057 | |  |  |
| Age group 6: Age group 2 | -0.687 | 0.112 | -6.158 | |  | | **<0.001** | |  |  |
| Age group 4: Age group 3 | -0.024 | 0.120 | -0.205 | |  | | 1 | |  |  |
| Age group 5: Age group 3 | -0.290 | 0.125 | -2.315 | |  | | 0.188 | |  |  |
| Age group 6: Age group 3 | -0.645 | 0.119 | -5.424 | |  | | **<0.001** | |  |  |
| Age group 5: Age group 4 | -0.266 | 0.124 | -2.150 | |  | | 0.262 | |  |  |
| Age group 6: Age group 4 | -0.621 | 0.117 | -5.302 | |  | | **<0.001** | |  |  |
| Age group 6: Age group 5 | -0.355 | 0.123 | -2.884 | |  | | **0.046** | |  |  |
| **Aggressiveness to animals (lambda = 0.46)** | | |  | |  | |  | |  |  |
| Age group |  |  |  | | 4.628 | | **<0.001** | | 0.019 | 0.015 |
| Age group 2: Age group 1 | 0.017 | 0.044 | 0.376 | |  | | 0.999 | |  |  |
| Age group 3: Age group 1 | 0.136 | 0.047 | 2.883 | |  | | **0.046** | |  |  |
| Age group 4: Age group 1 | 0.139 | 0.046 | 2.993 | |  | | **0.033** | |  |  |
| Age group 5: Age group 1 | 0.040 | 0.049 | 0.827 | |  | | 0.962 | |  |  |
| Age group 6: Age group 1 | -0.020 | 0.046 | -0.439 | |  | | 0.998 | |  |  |
| Age group 3: Age group 2 | 0.120 | 0.044 | 2.728 | |  | | 0.070 | |  |  |
| Age group 4: Age group 2 | 0.122 | 0.043 | 2.849 | |  | | 0.051 | |  |  |
| Age group 5: Age group 2 | 0.023 | 0.045 | 0.519 | |  | | 0.995 | |  |  |
| Age group 6: Age group 2 | -0.037 | 0.043 | -0.865 | |  | | 0.955 | |  |  |
| Age group 4: Age group 3 | 0.003 | 0.046 | 0.060 | |  | | 1.000 | |  |  |
| Age group 5: Age group 3 | -0.096 | 0.048 | -2.000 | |  | | 0.342 | |  |  |
| Age group 6: Age group 3 | -0.157 | 0.046 | -3.421 | |  | | **0.008** | |  |  |
| Age group 5: Age group 4 | -0.099 | 0.047 | -2.093 | |  | | 0.291 | |  |  |
| Age group 6: Age group 4 | -0.159 | 0.045 | -3.548 | |  | | **0.005** | |  |  |
| Age group 6: Age group 5 | -0.060 | 0.047 | -1.284 | |  | | 0.793 | |  |  |

## Main effect of age on the DPQ facets

Results of the Kruskal Wallis Tests showed significant age effects in the following 11 facets (out of 15): Fear of people, Non-social fear, Excitability, Playfulness, Active engagement, Companionability, Trainability, Controllability, Aggression towards animals, Prey drive and Dominance over dogs (all p values < 0.003; Figure 1). Fear of people peaked in dogs aged three to six years and was lowest in dogs aged over ten years. Non-social fear increased with age, with dogs aged over 12 years showing the highest levels, and dogs aged under three years the lowest levels. Excitability, Playfulness, and Active engagement all showed a significant linear decrease with age (highest scores in dogs aged one to three, and the lowest in dogs aged over 12 years). Trainability scores remained high until declining from ten years onwards. Controllability showed a quadratic distribution, peaking in three to six year olds, and was lowest in dogs aged over 12 years. Aggression towards dogs was lowest in the youngest age group, and highest in the oldest. Conversely, prey drive was highest in the youngest age group and lowest in the oldest. Finally, Dominance over dogs showed a quadratic distribution and peaked in dogs aged 8 to 10.

### *Figure 1 a-k:* Median Z score and interquartile range of the Dog Personality Questionnaire facets

c)

a)

a) Fear of people, b) Non-social fear, c) Excitability, d) Playfulness, e) Active engagement, f) Companionability, g) Trainability, h) Companionability, i) Aggression towards animals, j) Prey drive, and k) Dominance over dogs, in the six different dog age groups. For statistical differences, see text. Underneath each graph a table with a pairwise comparison of each age group is presented, along with test statistic, standard error, significance level, and adjusted significance level for multiple comparisons.


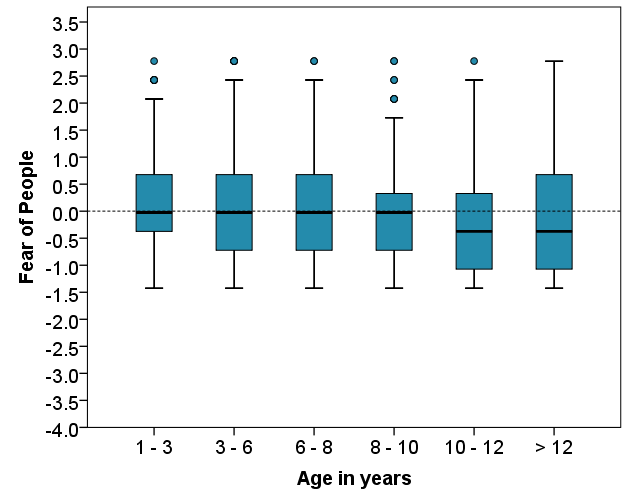


#### a)


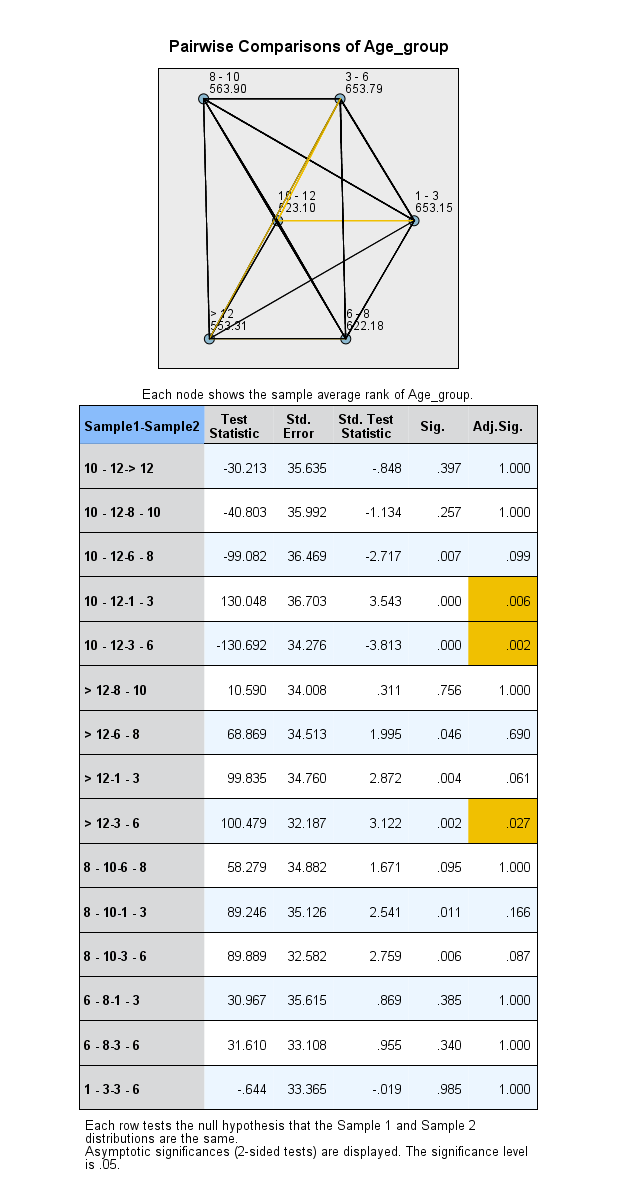


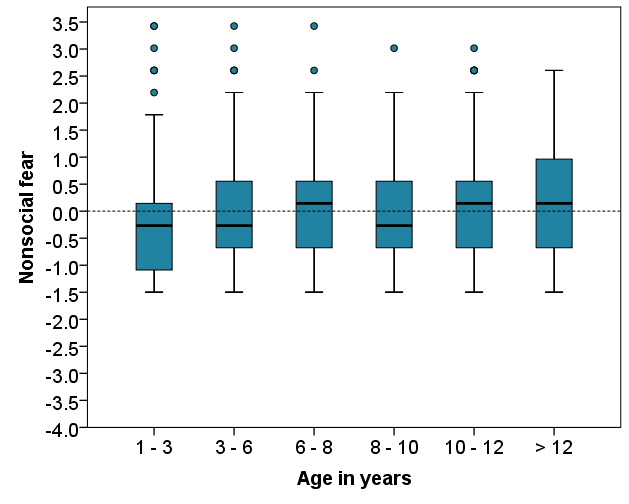


#### b)


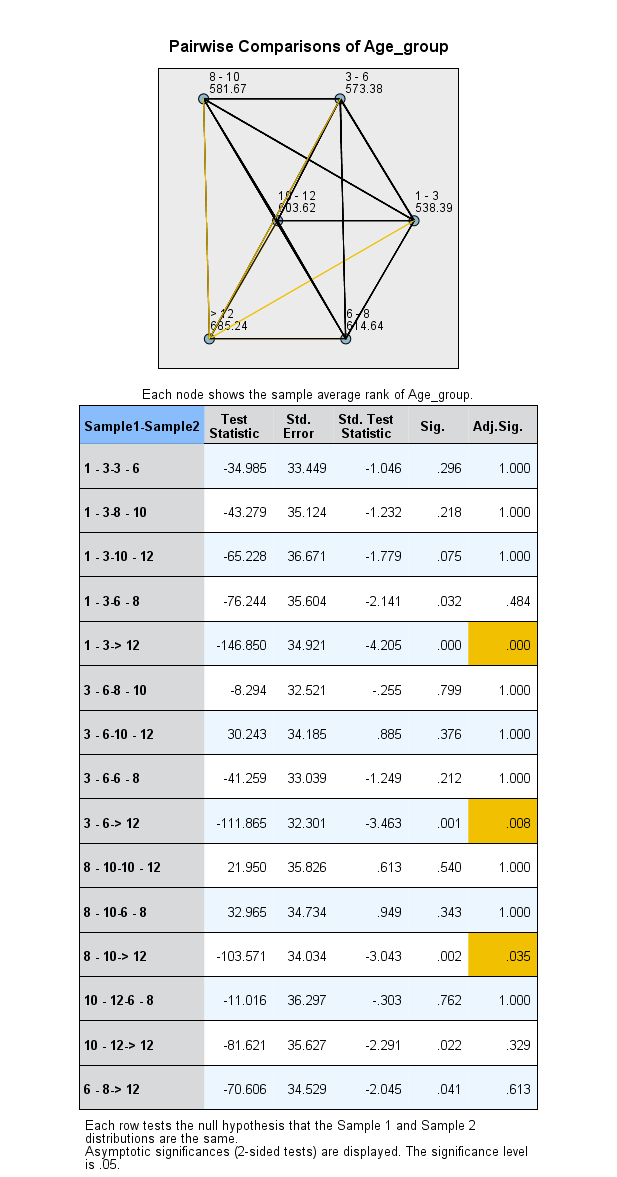


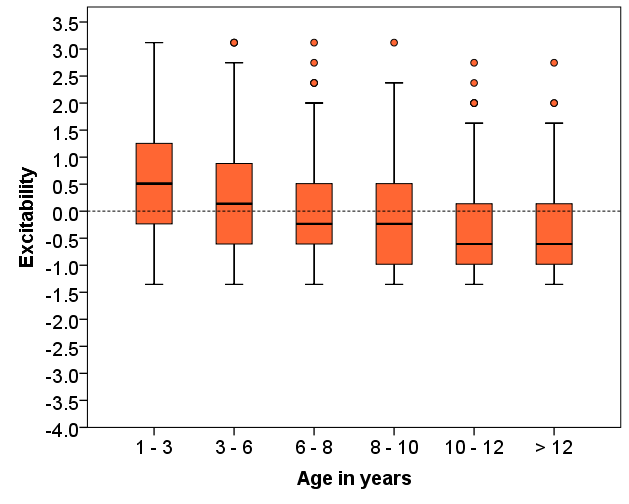

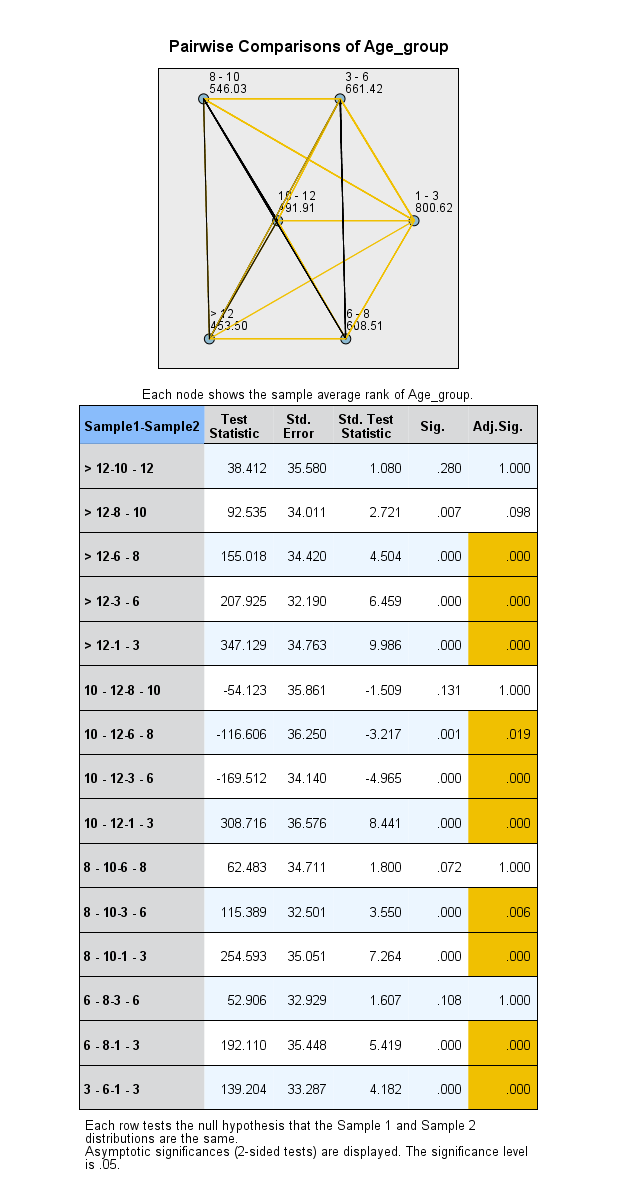


#### c)


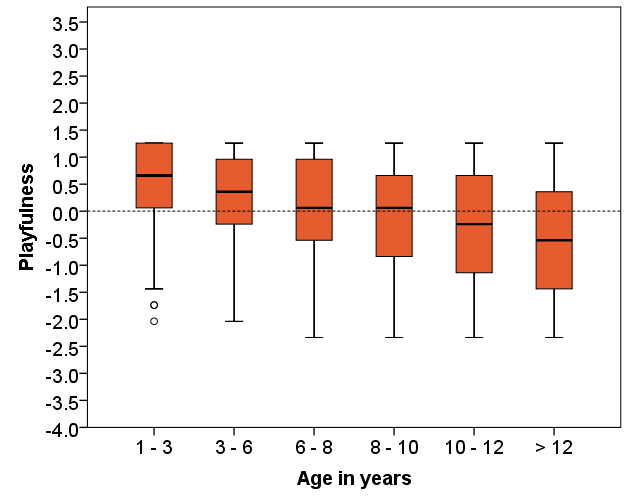


#### d)


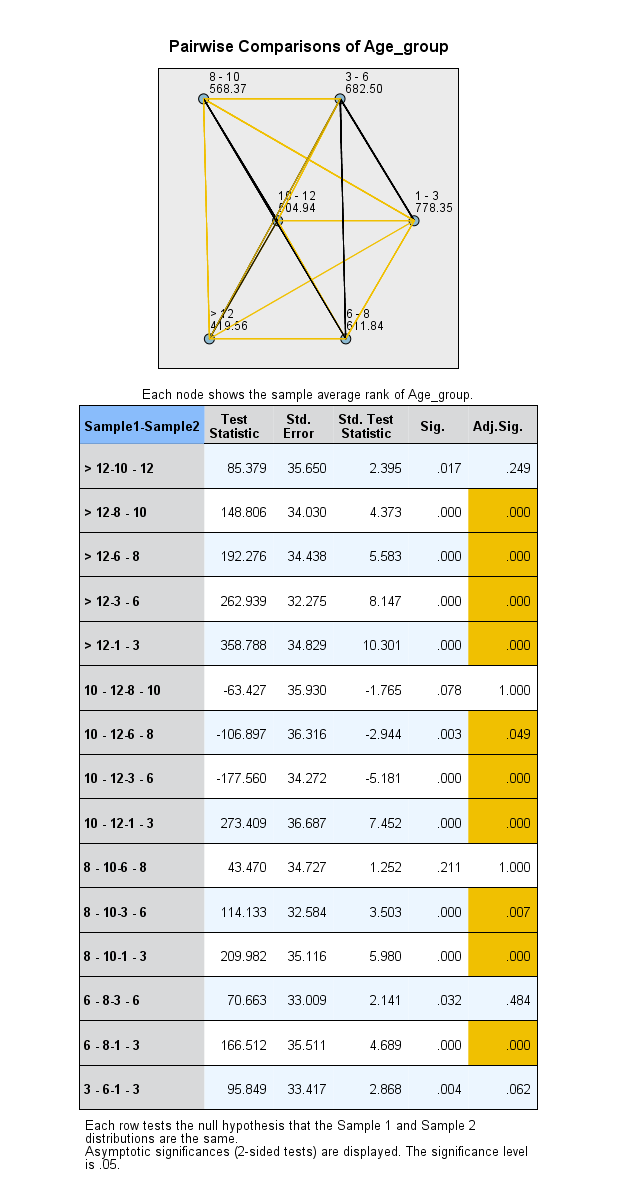


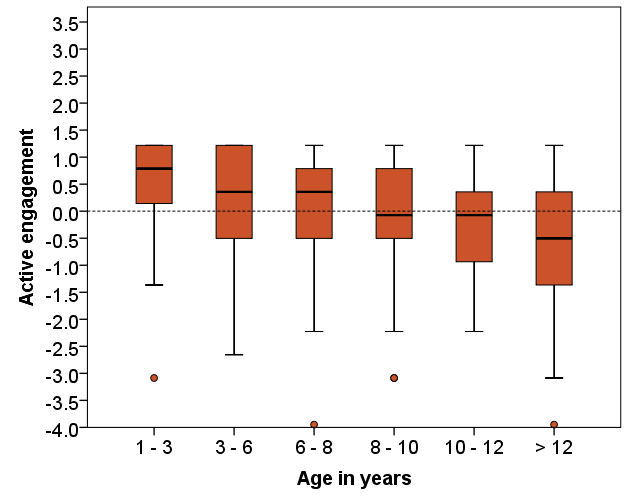


#### e)


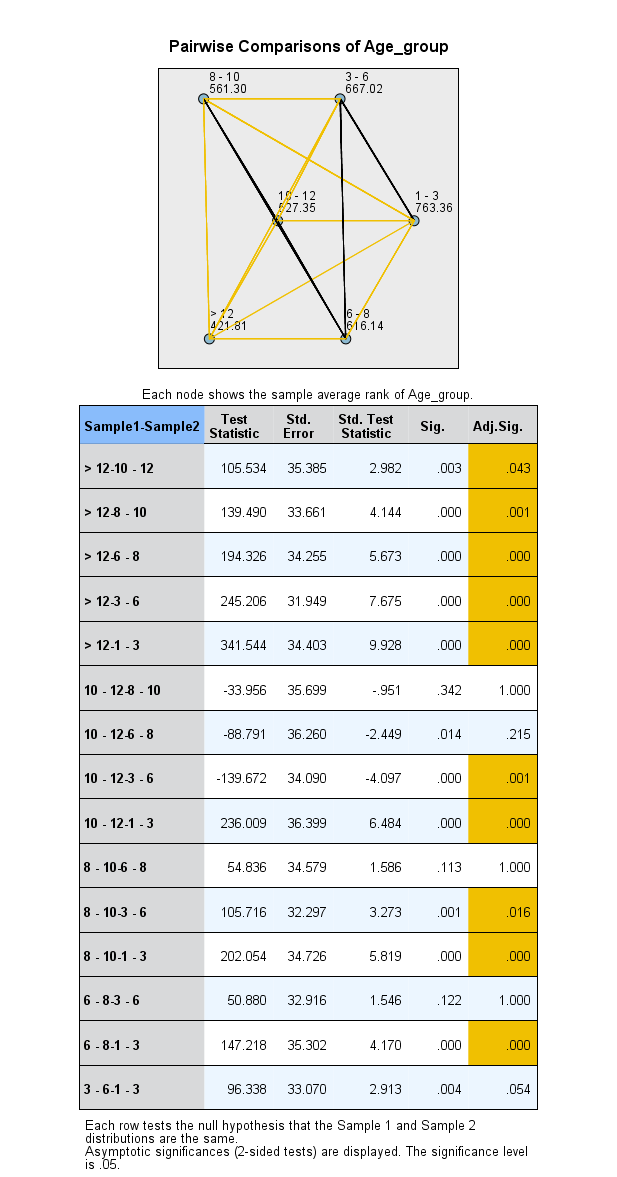


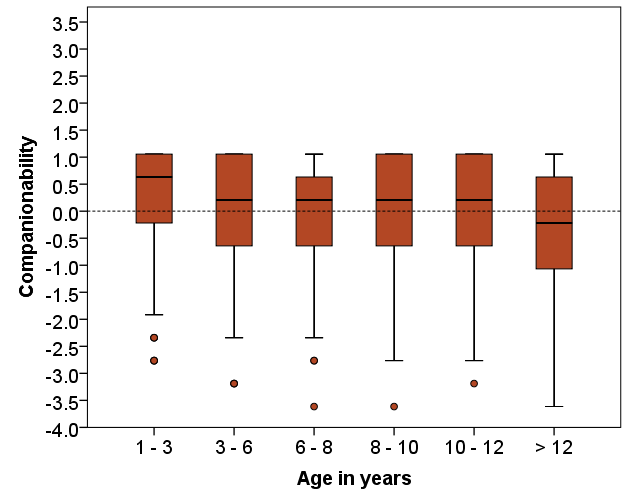


#### f)


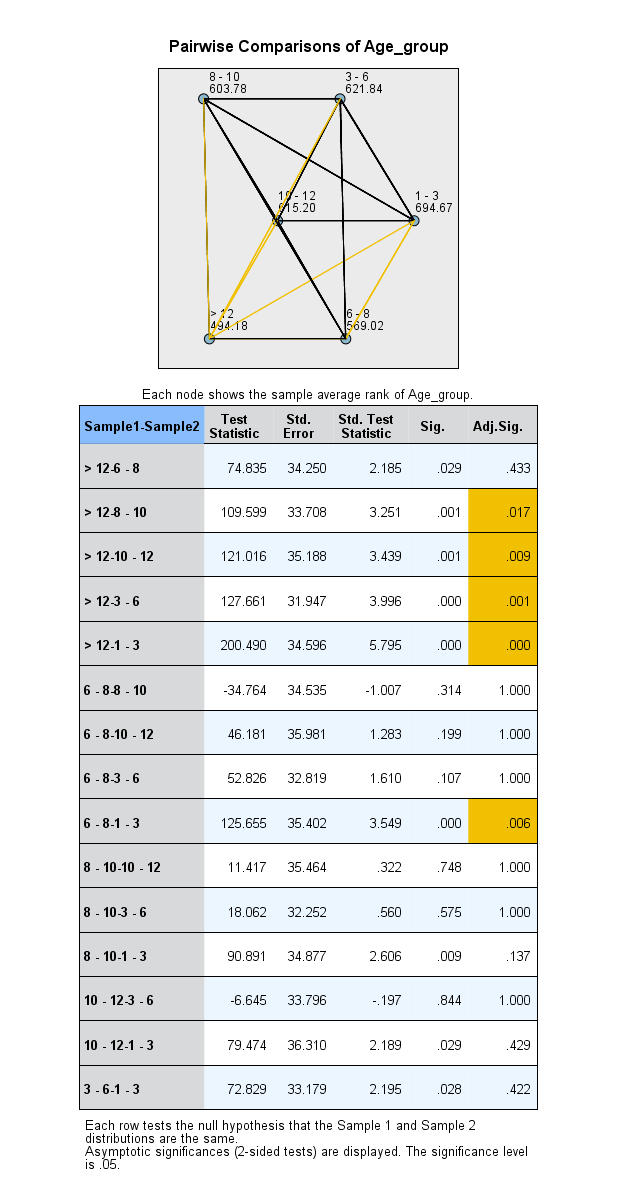


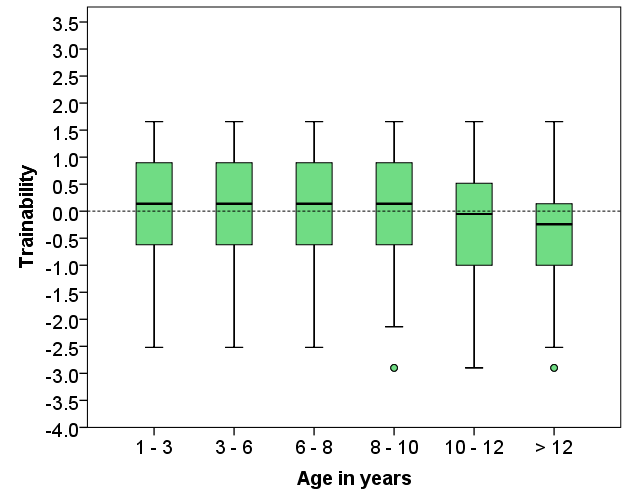


#### g)


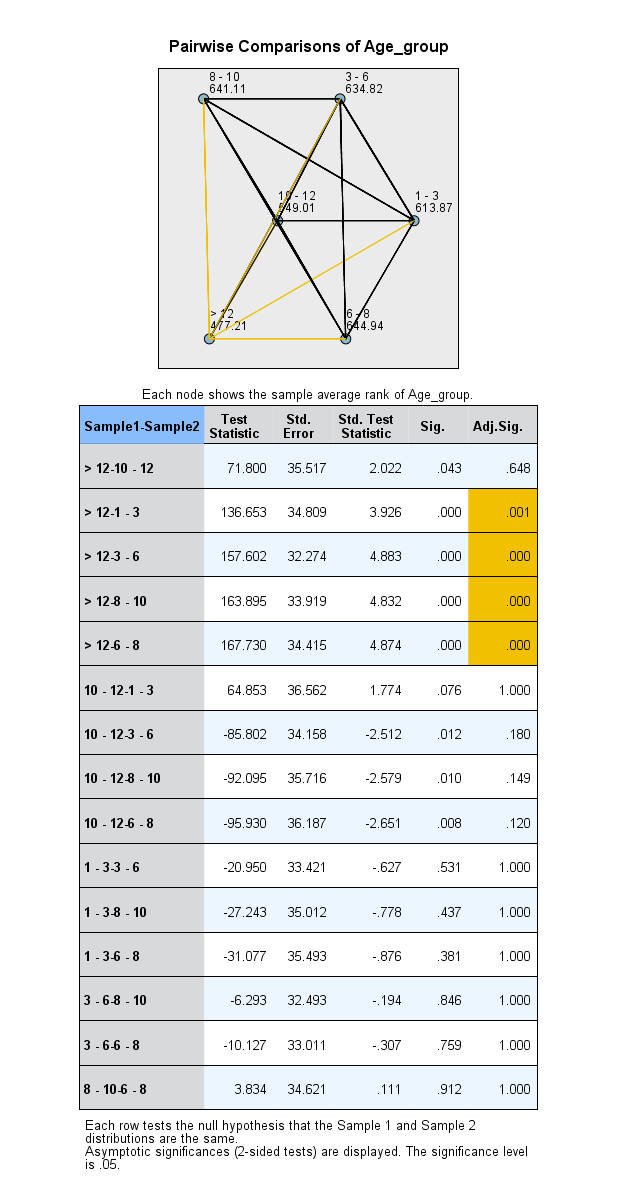


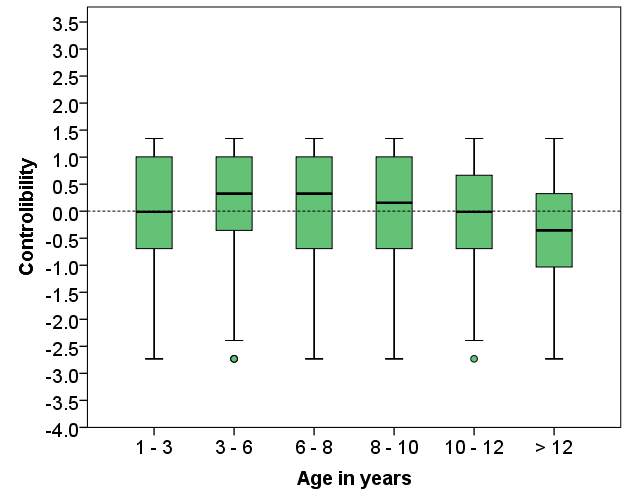


#### h)


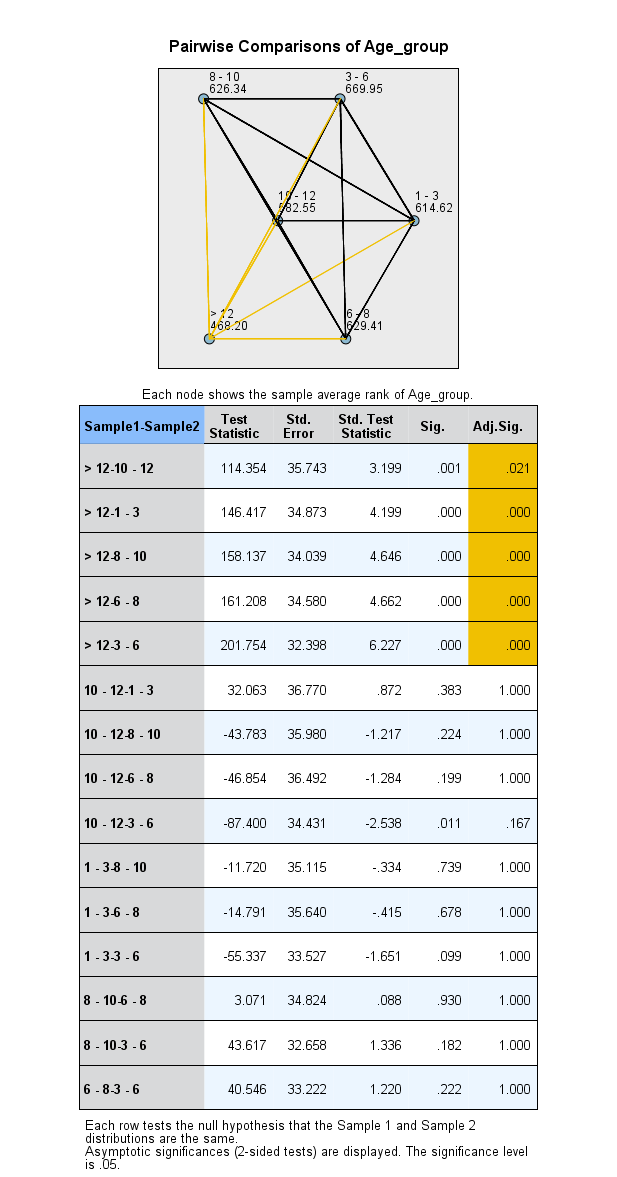


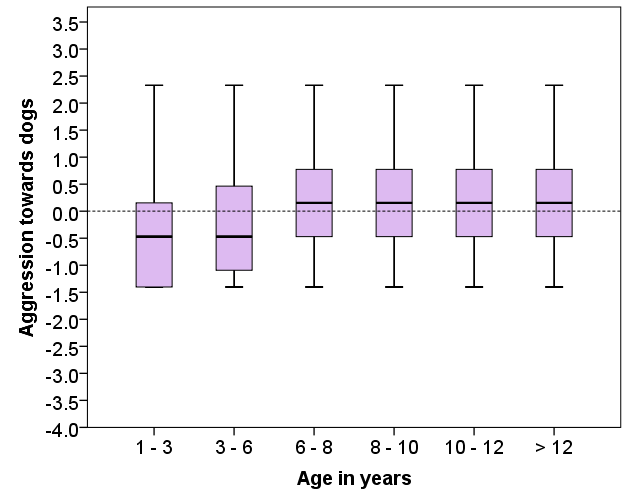


#### i)


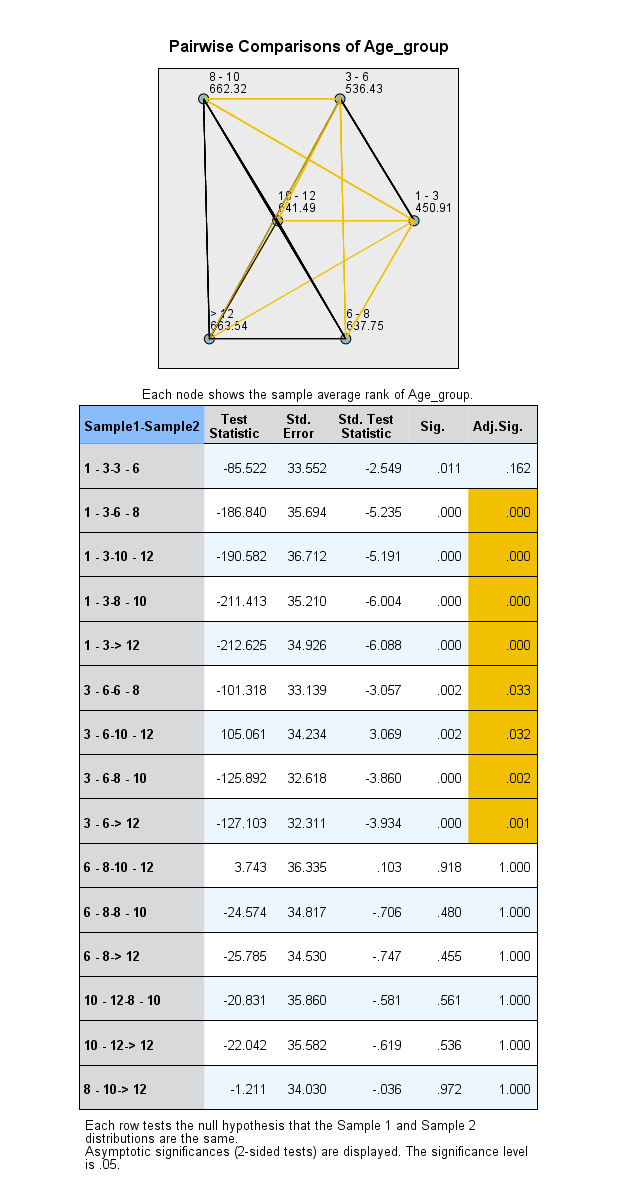


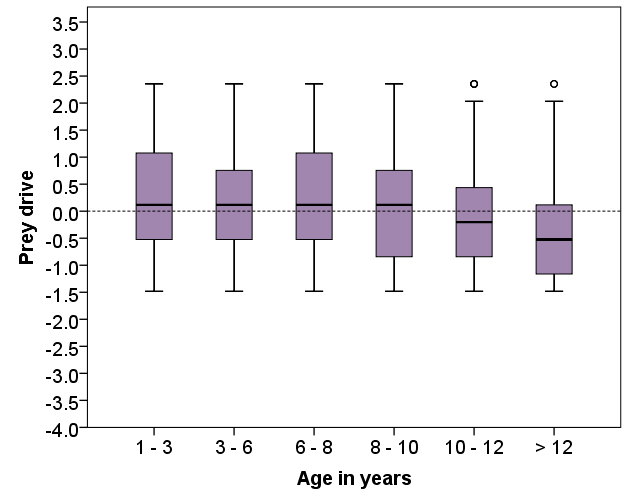


#### j)


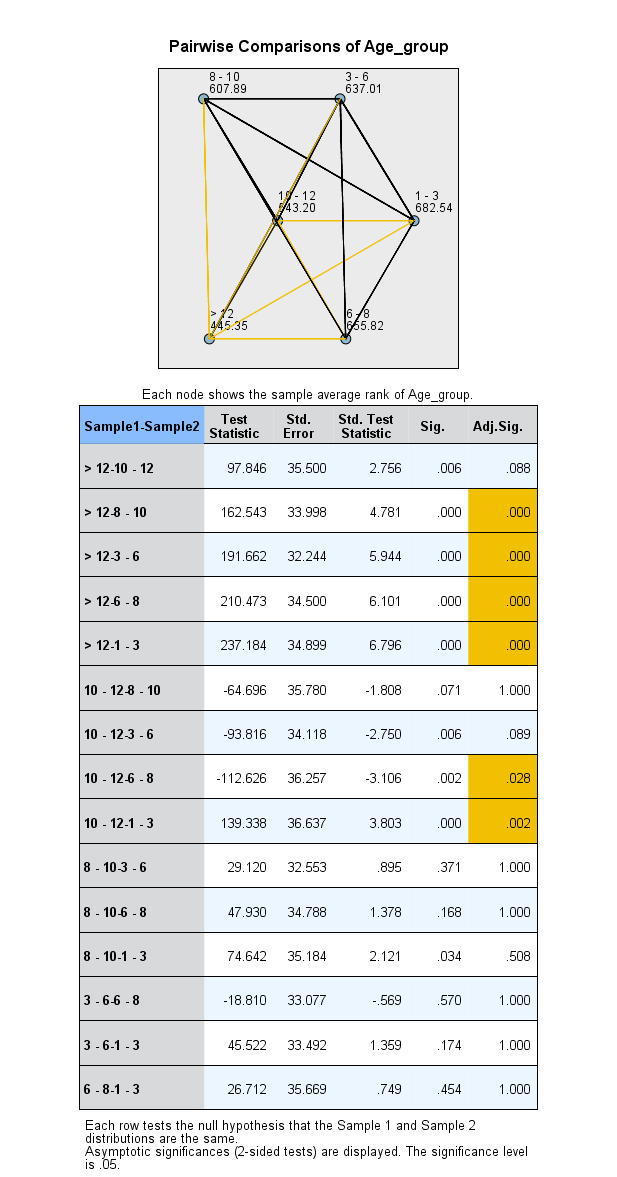


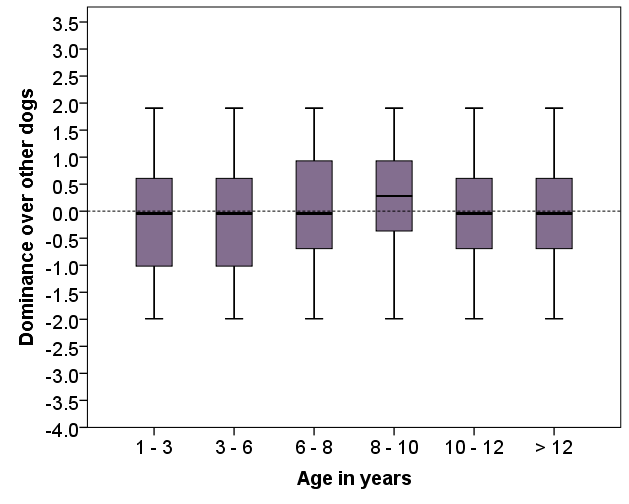


#### k)


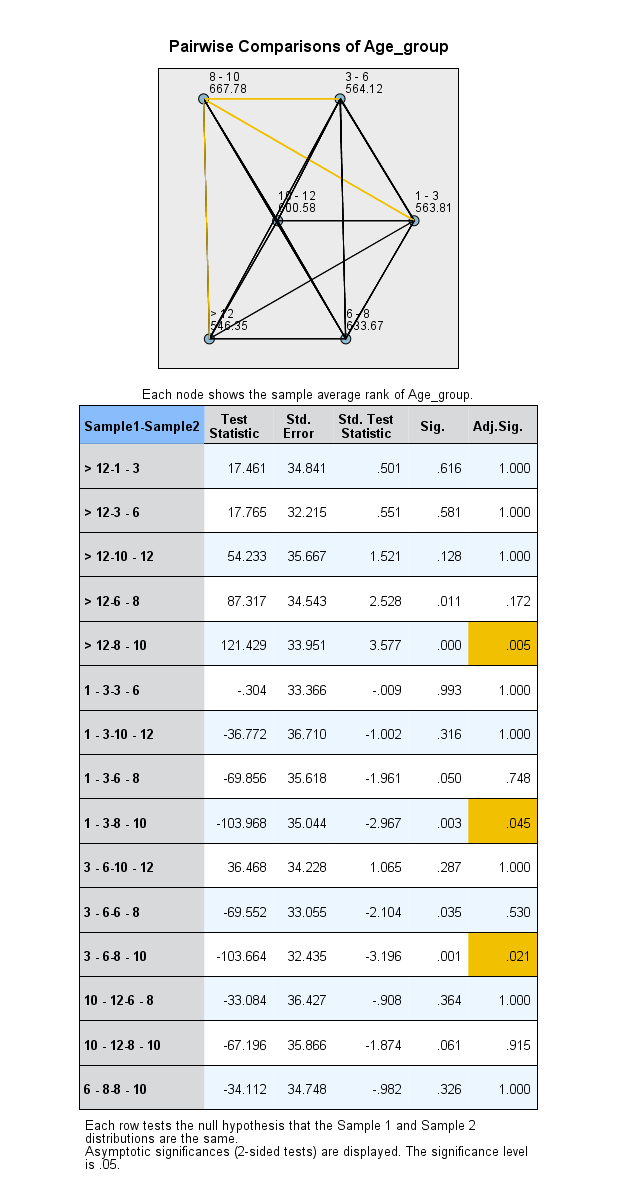


## Linear models: Main effects of the demographic variables

### Table 6: General linear models showing the direction of the effects and the significance level of the terms in the five factors of the DPQ.

Significant group differences are highlighted in bold (p≤0.05).

| **Response variable** | | | | **Category** | **Reference category** | | **Estimate** | | **SE** | | **t** | | **P** | |  |  |
| --- | --- | --- | --- | --- | --- | --- | --- | --- | --- | --- | --- | --- | --- | --- | --- | --- |
| **Fearfulness (lambda = 0.38)** | | | | |  | |  | |  | |  | |  | |  |  |
| Weight | | | | |  | | -0.007 | | 0.001 | | -5.391 | | **<0.001** | |  |  |
| Breed | | | Pure breed | | Mixed breed | | -0.118 | | 0.029 | | -4.070 | | **<0.001** | |  |  |
| Sex | | | Male | | Female | | -0.068 | | 0.022 | | -3.002 | | **0.003** | |  |  |
| Trauma | | | Yes | | No | | 0.155 | | 0.024 | | 6.382 | | **<0.001** | |  |  |
| Dog obedience tasks | | | | 2 kinds of tasks | maximum a task | | -0.043 | | 0.042 | | -1.019 | | 0.308 | |  |  |
|  | | | | 3 kinds of tasks | maximum a task | | -0.097 | | 0.040 | | -2.390 | | **0.017** | |  |  |
|  | | | | ≥ 4 kinds of tasks | maximum a task | | -0.150 | | 0.039 | | -3.862 | | **<0.001** | |  |  |
| Dog training activities | | | | 2 - 3 activities | One activity | | 0.033 | | 0.030 | | 1.112 | | 0.267 | |  |  |
|  | | | | 4 or more activities | One activity | | -0.105 | | 0.034 | | -3.061 | | **0.002** | |  |  |
| **Aggression towards people (lambda = -0.66)** | | | | | | |  | |  | |  | |  | |  |  |
| Breed: Pure breed | | | | Pure breed | Mixed breed | | -0.065 | 0.021 | | -3.180 | | **0.002** | |  |  |  |
| Sex: Male | | | | Male | Female | | 0.061 | 0.016 | | 3.793 | | **<0.001** | |  |  |  |
| Trauma | | | | Yes | No | | 0.081 | 0.017 | | 4.706 | | **<0.001** | |  |  |  |
| Dog obedience tasks | | | | 2 kinds of tasks | maximum a task | | -0.002 | | 0.030 | | -0.082 | | 0.934 | |  |  |
|  |  | | | 3 kinds of tasks | maximum a task | | -0.118 | | 0.029 | | -4.072 | | **<0.001** | |  |  |
|  |  | | | ≥ 4 kinds of tasks | maximum a task | | -0.126 | | 0.028 | | -4.530 | | **<0.001** | |  |  |
| **Activity Excitability (lambda = 1.51)** | | | | |  | |  | |  | |  | |  | |  |  |
| Age group | | | >3 - 6 years | | 1 - 3 years | | -0.394 | | 0.101 | | -3.884 | | **<0.001** | |  |  |
|  | | | >6 - 8 years | | 1 - 3 years | | -0.661 | | 0.109 | | -6.058 | | **<0.001** | |  |  |
|  | | | >8 - 10 years | | 1 - 3 years | | -0.764 | | 0.113 | | -6.752 | | **<0.001** | |  |  |
|  | | | >10 - 12 years | | 1 - 3 years | | -0.918 | | 0.120 | | -7.626 | | **<0.001** | |  |  |
|  | | | >12 years | | 1 - 3 years | | -1.090 | | 0.129 | | -8.473 | | **<0.001** | |  |  |
| Sensory problems | | Yes | | | No | | -0.240 | | 0.094 | | -2.555 | | **0.011** | |  |  |
| Body condition score | | | Normal | | Thin | | -0.003 | | 0.061 | | -0.038 | | 0.999 | |  |  |
|  | | | Overweight | | Thin | | -0.264 | | 0.104 | | -2.535 | | **0.030** | |  |  |
|  | | | Overweight | | Normal | | -0.261 | | 0.081 | | -3.240 | | **0.004** | |  |  |
| Age of dog when arrived | | | | 7-12 weeks | <7 weeks | | -0.208 | | 0.078 | | -2.677 | | **0.008** | |  |  |
|  |  | | | 3-12 months | <7 weeks | | -0.474 | | 0.099 | | -4.781 | | **<0.001** | |  |  |
|  |  | | | >1 year | <7 weeks | | -0.673 | | 0.109 | | -6.172 | | **<0.001** | |  |  |
| Get dog |  | | | It was born at my place/ I bought it | I found it/got it from a shelter | | -0.330 | | 0.105 | | -3.127 | | **0.002** | |  |  |
|  |  | | | I got it as a present | I found it/got it from a shelter | | -0.173 | | 0.092 | | -1.890 | | 0.059 | |  |  |
| Dog obedience tasks | | | | 2 kinds of tasks | maximum a task | | 0.302 | | 0.114 | | 2.649 | | **0.008** | |  |  |
|  |  | | | 3 kinds of tasks | maximum a task | | 0.321 | | 0.110 | | 2.918 | | **0.004** | |  |  |
|  |  | | | ≥ 4 kinds of tasks | maximum a task | | 0.617 | | 0.105 | | 5.888 | | **< 0.001** | |  |  |
| **Responsiveness to training (lambda = 1.31)** | | | | | | |  | |  | |  | |  | |  |  |
| Weight | |  | | | |  | 0.009 | | 0.003 | | 2.653 | | **0.008** | |  |  |
| Sex | | Male | | | | Female | -0.181 | | 0.056 | | -3.215 | | **0.001** | |  |  |
| Off-leash activity | | 30 minutes -1 hour | | | | < 30 minutes | 0.135 | | 0.093 | | 1.440 | | 0.150 | |  |  |
|  | | >1-3 hours | | | | < 30 minutes | 0.316 | | 0.091 | | 3.482 | | **<0.001** | |  |  |
|  | | >3-7 hours | | | | < 30 minutes | 0.315 | | 0.107 | | 2.936 | | **0.003** | |  |  |
|  | | more than 7 hours | | | | < 30 minutes | 0.220 | | 0.106 | | 2.087 | | **0.037** | |  |  |
| Dog obedience tasks | | 2 kinds of tasks | | | | maximum a task | 0.251 | | 0.105 | | 2.396 | | **0.017** | |  |  |
|  | | 3 kinds of tasks | | | | maximum a task | 0.665 | | 0.100 | | 6.626 | | **<0.001** | |  |  |
|  | | ≥ 4 kinds of tasks | | | | maximum a task | 1.326 | | 0.096 | | 13.780 | | **<0.001** | |  |  |
| Play | | 30 minutes -1 hours | | | | < 30 minutes | 0.182 | | 0.099 | | 1.840 | | 0.066 | |  |  |
|  | | >1 -3 hours | | | | < 30 minutes | 0.287 | | 0.100 | | 2.854 | | **0.004** | |  |  |
|  | | more than 3 hours | | | | < 30 minutes | 0.402 | | 0.121 | | 3.316 | | **< 0.001** | |  |  |
| Commands | | 11 - 30 | | | | < 10 | 0.308 | | 0.068 | | 4.564 | | **< 0.001** | |  |  |
|  | | >31 | | | | < 10 | 0.657 | | 0.108 | | 6.058 | | **< 0.001** | |  |  |
| **Aggressiveness towards animals (lambda = 0.46)** | | | | | | |  | |  | |  | |  | |  |  |
| Age group |  | | | >3 - 6 years | 1 - 3 years | | 0.020 | | 0.045 | | 0.449 | | 0.653 | |  |  |
|  |  | | | >6 - 8 years | 1 - 3 years | | 0.126 | | 0.049 | | 2.586 | | **0.010** | |  |  |
|  |  | | | >8 - 10 years | 1 - 3 years | | 0.119 | | 0.050 | | 2.392 | | **0.017** | |  |  |
|  |  | | | >10 - 12 years | 1 - 3 years | | 0.026 | | 0.053 | | 0.484 | | 0.628 | |  |  |
|  |  | | | >12 years | 1 - 3 years | | -0.057 | | 0.057 | | -0.985 | | 0.325 | |  |  |
| Food |  | | | Tinned &/or dry food | Dry food only | | -0.105 | | 0.047 | | -2.221 | | 0.171 | |  |  |
|  |  | | | Cooked food | Dry food only | | 0.025 | | 0.039 | | 0.662 | | 0.964 | |  |  |
|  |  | | | Mixed | Dry food only | | 0.062 | | 0.040 | | 1.523 | | 0.545 | |  |  |
|  |  | | | Raw meat | Dry food only | | 0.038 | | 0.043 | | 0.890 | | 0.900 | |  |  |
|  |  | | | Cooked food | Tinned &/or dry food | | 0.130 | | 0.045 | | 2.867 | | **0.034** | |  |  |
|  |  | | | Mixed | Tinned &/or dry food | | 0.166 | | 0.047 | | 3.540 | | **0.004** | |  |  |
|  |  | | | Raw meat | Tinned &/or dry food | | 0.143 | | 0.050 | | 2.843 | | **0.036** | |  |  |
|  |  | | | Mixed | Cooked food | | 0.036 | | 0.039 | | 0.933 | | 0.883 | |  |  |
|  |  | | | Raw meat | Cooked food | | 0.013 | | 0.042 | | 0.302 | | 0.998 | |  |  |
|  |  | | | Raw meat | Mixed | | -0.023 | | 0.043 | | -0.544 | | 0.982 | |  |  |
| Trauma |  | | | Yes | No | | 0.085 | | 0.029 | | 2.916 | | **0.004** | |  |  |
| Dog obedience tasks | | | | 2 kinds of tasks | maximum a task | | 0.059 | | 0.051 | | 1.165 | | 0.244 | |  |  |
|  | | | | 3 kinds of tasks | maximum a task | | -0.053 | | 0.049 | | -1.096 | | 0.273 | |  |  |
|  |  | | | ≥ 4 kinds of tasks | maximum a task | | -0.135 | | 0.047 | | -2.891 | | **0.004** | |  |  |

## Dog Personality Factor Linear Models R code

### DPQFAC1 – Fearfulness

#### Age only

> lm<-lm(bcPower(DPQFAC1new,0.38)~(age), data = data, na.action = na.omit)

> etasq(lm, anova = TRUE, type=3)

Anova Table (Type III tests)

Response: bcPower(DPQFAC1new, 0.38)

Partial eta^2 Sum Sq Df F value Pr(>F)

(Intercept) 0.45090 154.92 1 955.8315 <2e-16 ***

age 0.00574 1.09 5 1.3447 0.2429

Residuals 188.66 1164

---

Signif. codes: 0 ‘***’ 0.001 ‘**’ 0.01 ‘*’ 0.05 ‘.’ 0.1 ‘ ’ 1

> summary(lm)

Call:

lm(formula = bcPower(DPQFAC1new, 0.38) ~ (age), data = data,

na.action = na.omit)

Residuals:

Min 1Q Median 3Q Max

-0.9387 -0.2897 0.0142 0.2832 1.0270

Coefficients:

Estimate Std. Error t value Pr(>|t|)

(Intercept) 0.935562 0.030261 30.917 <2e-16 ***

ageGroup2 0.057580 0.039715 1.450 0.147

ageGroup3 0.049773 0.042330 1.176 0.240

ageGroup4 0.003163 0.041645 0.076 0.939

ageGroup5 -0.030145 0.043705 -0.690 0.490

ageGroup6 0.035709 0.041450 0.862 0.389

---

Signif. codes: 0 ‘***’ 0.001 ‘**’ 0.01 ‘*’ 0.05 ‘.’ 0.1 ‘ ’ 1

Residual standard error: 0.4026 on 1164 degrees of freedom

(37 observations deleted due to missingness)

Multiple R-squared: 0.005743, Adjusted R-squared: 0.001472

F-statistic: 1.345 on 5 and 1164 DF, p-value: 0.2429

> Residuals <- residuals (lm)

> qqnorm(Residuals)

> shapiro.test (Residuals)

Shapiro-Wilk normality test

data: Residuals

W = 0.99363, p-value = 6.623e-05

#### Full Model

> lm<-lm(bcPower(DPQFAC1new,0.38)~(age+sensory+height+weight+breed+sex+neuter+offleash_activity+BCS+food+Add_vitamins+trauma+Health_prob+medication+Owner_age+Owner_dog_exp+How_many_dogs_in_house+How_many_people+Child+Age_of_dog_when_arrived+get_dog+Where_dog_is_kept+dog_obedience+play+commands+training+Time_dog_spend_alone+dog_beh_changed_3month), data = data, na.action = na.omit)

> etasq(lm, anova = TRUE, type=3)

Anova Table (Type III tests)

Response: bcPower(DPQFAC1new, 0.38)

Partial eta^2 Sum Sq Df F value Pr(>F)

(Intercept) 0.097047 15.543 1 118.9769 < 2.2e-16 ***

Age 0.011822 1.730 5 2.6487 0.0217269 *

sensory 0.001563 0.226 1 1.7332 0.1882778

height 0.000200 0.029 1 0.2211 0.6383243

weight 0.024473 3.628 1 27.7712 1.640e-07 ***

breed 0.013463 1.973 1 15.1066 0.0001076 ***

sex 0.009584 1.399 1 10.7122 0.0010972 **

neuter 0.002912 0.422 1 3.2333 0.0724270 .

offleash_activity 0.004839 0.703 4 1.3457 0.2509659

BCS 0.000839 0.121 2 0.4646 0.6285174

food 0.002624 0.380 4 0.7281 0.5727854

Add_vitamins 0.002431 0.352 3 0.8991 0.4410215

trauma 0.034761 5.208 1 39.8665 3.929e-10 ***

Health_prob 0.009200 1.343 4 2.5696 0.0365694 *

medication 0.000033 0.005 1 0.0361 0.8493583

Owner_age 0.001977 0.287 3 0.7311 0.5335260

Owner_dog_exp 0.001530 0.222 2 0.8481 0.4285127

How_many_dogs_in_house 0.001564 0.227 2 0.8671 0.4204684

How_many_people 0.001308 0.189 3 0.4832 0.6939953

Child 0.000039 0.006 1 0.0432 0.8354138

Age_of_dog_when_arrived 0.002636 0.382 3 0.9752 0.4036565

get_dog 0.001692 0.245 2 0.9381 0.3916660

Where_dog_is_kept 0.002095 0.304 2 1.1619 0.3132760

dog_obedience 0.016579 2.438 3 6.2209 0.0003443 ***

play 0.000416 0.060 3 0.1537 0.9273334

commands 0.000680 0.098 2 0.3768 0.6861150

training 0.011852 1.735 2 6.6390 0.0013610 **

Time_dog_spend_alone 0.004837 0.703 3 1.7934 0.1466462

dog_beh_changed_3month 0.003987 0.579 1 4.4315 0.0355062 *

Residuals 144.616 1107

---

Signif. codes: 0 ‘***’ 0.001 ‘**’ 0.01 ‘*’ 0.05 ‘.’ 0.1 ‘ ’ 1

> summary(lm)

Call:

lm(formula = bcPower(DPQFAC1new, 0.38) ~ (age3 + sensory2 + height +

weight + breed + sex + neuter2 + offleash_activity + shape +

food + Add_vitamins + trauma + Health_prob + medication +

Owner_age + Owner_dog_exp + How_many_dogs_in_house + How_many_people +

Child + Age_of_dog_when_arrived + get_dog + Where_dog_is_kept2 +

dog_jobs + play + commands + training + Time_dog_spend_alone +

dog_beh_changed_3month), data = new_cog_no_missing3, na.action = na.omit)

Residuals:

Min 1Q Median 3Q Max

-1.03946 -0.26056 0.00112 0.25700 1.28626

Coefficients:

Estimate Std. Error t value Pr(>|t|)

(Intercept) 1.195e+00 1.095e-01 10.908 < 2e-16 ***

ageGroup2 2.647e-02 3.774e-02 0.701 0.483271

ageGroup3 1.203e-02 4.055e-02 0.297 0.766829

ageGroup4 -6.371e-02 4.206e-02 -1.515 0.130090

ageGroup5 -9.928e-02 4.477e-02 -2.218 0.026781 *

ageGroup6 -6.170e-02 4.820e-02 -1.280 0.200788

sensory_probPresent -4.617e-02 3.507e-02 -1.317 0.188278

height 5.067e-04 1.078e-03 0.470 0.638324

weight -7.062e-03 1.340e-03 -5.270 1.64e-07 ***

breedPure -1.127e-01 2.900e-02 -3.887 0.000108 ***

sexMale -7.489e-02 2.288e-02 -3.273 0.001097 **

neuterNeutered 4.414e-02 2.454e-02 1.798 0.072427 .

offleash_activity30m-1h 1.329e-03 3.798e-02 0.035 0.972096

offleash_activity1-3h -1.003e-02 3.665e-02 -0.274 0.784419

offleash_activity2-7 -2.557e-02 4.347e-02 -0.588 0.556589

offleash_activity>7 -7.787e-02 4.263e-02 -1.827 0.068042 .

BCSNormal -2.216e-02 3.034e-02 -0.730 0.465449

BCSOverweight 3.593e-05 3.914e-02 0.001 0.999268

foodTinned dry 2.859e-02 4.010e-02 0.713 0.476011

foodCooked -2.089e-03 3.241e-02 -0.064 0.948623

foodMixed 1.677e-02 3.398e-02 0.493 0.621865

foodRaw -3.566e-02 3.596e-02 -0.992 0.321516

Add_vitaminsRarely 1.818e-02 2.869e-02 0.634 0.526426

Add_vitaminsOften -2.398e-02 3.289e-02 -0.729 0.466234

Add_vitaminsDaily 2.602e-02 3.439e-02 0.757 0.449507

traumaYes 1.545e-01 2.447e-02 6.314 3.93e-10 ***

Health_probTooth only 4.517e-02 3.635e-02 1.243 0.214259

Health_probJoint & Tooth 8.110e-02 4.323e-02 1.876 0.060895 .

Health_probJoint only 2.791e-04 3.318e-02 0.008 0.993291

Health_probOther 9.189e-02 3.590e-02 2.560 0.010611 *

medicationYes 6.195e-03 3.261e-02 0.190 0.849358

Owner_age30-39 -1.888e-02 3.093e-02 -0.610 0.541732

Owner_age40-49 2.675e-02 3.437e-02 0.778 0.436496

Owner_age>50 1.378e-02 3.614e-02 0.381 0.702983

Owner_dog_expHad dog 5.720e-03 2.851e-02 0.201 0.841031

Owner_dog_expNever had dog 3.927e-02 3.441e-02 1.141 0.253966

How_many_dogs_in_houseOne -7.734e-03 2.513e-02 -0.308 0.758290

How_many_dogs_in_houseTwo more -3.928e-02 3.074e-02 -1.278 0.201550

How_many_peopleTwo -1.643e-02 3.693e-02 -0.445 0.656570

How_many_peopleThree 1.715e-02 4.468e-02 0.384 0.701112

How_many_peopleFour more -1.290e-02 4.579e-02 -0.282 0.778238

ChildYes 6.777e-03 3.261e-02 0.208 0.835414

Age_of_dog_when_arrived7-12w 2.329e-02 2.871e-02 0.811 0.417254

Age_of_dog_when_arrived3-12m -2.839e-02 3.685e-02 -0.770 0.441207

Age_of_dog_when_arrived>1y -2.719e-02 4.039e-02 -0.673 0.500959

get_dogBorn bought 1.028e-02 3.922e-02 0.262 0.793228

get_dogPresent 3.955e-02 3.407e-02 1.161 0.245918

Where_dog_is_keptGarden 5.770e-02 3.861e-02 1.494 0.135393

Where_dog_is_keptApartment 2.860e-02 3.620e-02 0.790 0.429654

dog_obedience2 tasks -4.592e-02 4.253e-02 -1.080 0.280455

dog_obedience3tasks -1.014e-01 4.067e-02 -2.494 0.012786 *

dog_obedience>3 -1.551e-01 3.899e-02 -3.978 7.39e-05 ***

play30m-1h 1.398e-02 3.966e-02 0.353 0.724483

play1-3h 1.291e-03 4.026e-02 0.032 0.974431

play>3h -9.255e-03 4.901e-02 -0.189 0.850267

commands11-30 -5.344e-03 2.743e-02 -0.195 0.845574

commands>30 -3.665e-02 4.420e-02 -0.829 0.407077

training2-3 3.697e-02 2.992e-02 1.236 0.216874

training4 more -9.897e-02 3.443e-02 -2.874 0.004126 **

Time_dog_spend_alone1-2h 4.250e-02 3.758e-02 1.131 0.258339

Time_dog_spend_alone3-8h -1.832e-02 3.376e-02 -0.543 0.587462

Time_dog_spend_alone>8h 2.499e-02 4.288e-02 0.583 0.560189

dog_beh_changed_3monthYes 5.537e-02 2.630e-02 2.105 0.035506 *

---

Signif. codes: 0 ‘***’ 0.001 ‘**’ 0.01 ‘*’ 0.05 ‘.’ 0.1 ‘ ’ 1

Residual standard error: 0.3614 on 1107 degrees of freedom

(37 observations deleted due to missingness)

Multiple R-squared: 0.2379, Adjusted R-squared: 0.1952

F-statistic: 5.573 on 62 and 1107 DF, p-value: < 2.2e-16

> Residuals <- residuals (lm)

> qqnorm(Residuals)

> shapiro.test (Residuals)

Shapiro-Wilk normality test

data: Residuals

W = 0.99753, p-value = 0.07197

#### Reduced Model

> lm<-lm(bcPower(DPQFAC1new,0.38)~(age+sensory+height+weight+breed+sex+neuter+offleash_activity+BCS+food+Add_vitamins+trauma+Health_prob+medication+Owner_age+Age_of_dog_when_arrived+get_dog+Where_dog_is_kept+dog_obedience+play+commands+training+dog_beh_changed_3month), data = data, na.action = na.omit)

> etasq(lm, anova = TRUE, type=3)

Anova Table (Type III tests)

Response: bcPower(DPQFAC1new, 0.38)

Partial eta^2 Sum Sq Df F value Pr(>F)

(Intercept) 0.124829 20.844 1 159.4640 < 2.2e-16 ***

age 0.011887 1.758 5 2.6900 0.0200043 *

sensory 0.001968 0.288 1 2.2046 0.1378832

height 0.000174 0.025 1 0.1944 0.6593568

weight 0.025333 3.798 1 29.0580 8.565e-08 ***

breed 0.014598 2.165 1 16.5623 5.039e-05 ***

sex 0.007998 1.178 1 9.0134 0.0027397 **

neuter 0.002830 0.415 1 3.1731 0.0751303 .

offleash_activity 0.005374 0.790 4 1.5102 0.1969222

BCS 0.000735 0.108 2 0.4112 0.6629554

food 0.003315 0.486 4 0.9297 0.4458267

Add_vitamins 0.002058 0.301 3 0.7686 0.5116739

trauma 0.035154 5.325 1 40.7336 2.551e-10 ***

Health_prob 0.009634 1.422 4 2.7190 0.0285061 *

medication 0.000267 0.039 1 0.2986 0.5848677

Owner_age 0.002695 0.395 3 1.0072 0.3887268

Age_of_dog_when_arrived 0.003112 0.456 3 1.1633 0.3225946

get_dog 0.002077 0.304 2 1.1633 0.3128384

Where_dog_is_kept 0.002583 0.378 2 1.4476 0.2355703

dog_obedience 0.015573 2.312 3 5.8952 0.0005433 ***

play 0.000243 0.036 3 0.0906 0.9652342

commands 0.000929 0.136 2 0.5200 0.5946902

training 0.012458 1.844 2 7.0517 0.0009050 ***

dog_beh_changed_3month 0.003734 0.548 1 4.1908 0.0408782 *

Residuals 146.140 1118

---

Signif. codes: 0 ‘***’ 0.001 ‘**’ 0.01 ‘*’ 0.05 ‘.’ 0.1 ‘ ’ 1

> summary(lm)

Call:

lm(formula = bcPower(DPQFAC1new, 0.38) ~ (age + sensory + height +

weight + breed + sex + neuter + offleash_activity + BCS +

food + Add_vitamins + trauma + Health_prob + medication +

Owner_age + Age_of_dog_when_arrived + get_dog + Where_dog_is_kept +

dog_obedience + play + commands + training + dog_beh_changed_3month),

data = data, na.action = na.omit)

Residuals:

Min 1Q Median 3Q Max

-1.02791 -0.25785 0.00955 0.25295 1.27588

Coefficients:

Estimate Std. Error t value Pr(>|t|)

(Intercept) 1.1909668 0.0943123 12.628 < 2e-16 ***

ageGroup2 0.0224045 0.0375385 0.597 0.550734

ageGroup3 0.0133799 0.0404446 0.331 0.740842

ageGroup4 -0.0658520 0.0417041 -1.579 0.114612

ageGroup5 -0.0995403 0.0444756 -2.238 0.025412 *

ageGroup6 -0.0681230 0.0478595 -1.423 0.154900

sensoryPresent -0.0518317 0.0349086 -1.485 0.137883

height 0.0004722 0.0010710 0.441 0.659357

weight -0.0071812 0.0013322 -5.391 8.56e-08 ***

breedPure -0.1175308 0.0288796 -4.070 5.04e-05 ***

sexMale -0.0681134 0.0226876 -3.002 0.002740 **

neuterNeutered 0.0435394 0.0244421 1.781 0.075130 .

offleash_activity30mins/1hour 0.0022214 0.0377642 0.059 0.953103

offleash_activity1/3 -0.0088849 0.0365374 -0.243 0.807916

offleash_activity3/7 -0.0267173 0.0430407 -0.621 0.534894

offleash_activity>7 -0.0809671 0.0423742 -1.911 0.056291 .

BCSNormal -0.0181089 0.0300089 -0.603 0.546331

BCSOverweight 0.0048653 0.0387491 0.126 0.900105

foodTinned and/or Dry food 0.0326935 0.0397199 0.823 0.410626

foodCooked food 0.0011362 0.0319948 0.036 0.971678

foodMixed 0.0127531 0.0333805 0.382 0.702496

foodRaw -0.0426014 0.0357700 -1.191 0.233914

Add_vitaminsRarely 0.0096573 0.0284679 0.339 0.734497

Add_vitaminsOften -0.0316406 0.0325334 -0.973 0.330984

Add_vitaminsDaily 0.0135347 0.0339854 0.398 0.690520

traumaYes 0.1553504 0.0243408 6.382 2.55e-10 ***

Health_probTooth problem only 0.0478190 0.0361099 1.324 0.185687

Health_probJoint & Tooth probs 0.0879641 0.0429820 2.047 0.040937 *

Health_probJoint problems only 0.0027748 0.0329954 0.084 0.932995

Health_probOther disorders 0.0929146 0.0356156 2.609 0.009207 **

medicationYes 0.0176668 0.0323302 0.546 0.584868

Owner_age30-39 -0.0204600 0.0285536 -0.717 0.473804

Owner_age40-49 0.0318045 0.0307405 1.035 0.301074

Owner_age>50 0.0141278 0.0326543 0.433 0.665355

Age_of_dog_when_arrived7-12w 0.0244779 0.0284406 0.861 0.389606

Age_of_dog_when_arrived3-12m -0.0317563 0.0367096 -0.865 0.387187

Age_of_dog_when_arrived>1y -0.0304995 0.0403080 -0.757 0.449412

get_dogborn bought 0.0128509 0.0389149 0.330 0.741287

get_dogpresent 0.0443872 0.0337859 1.314 0.189191

Where_dog_is_keptGarden 0.0647794 0.0383700 1.688 0.091635 .

Where_dog_is_keptApartment 0.0354109 0.0357507 0.990 0.322146

dog_obedience2 tasks -0.0430889 0.0422838 -1.019 0.308405

dog_obedience3 tasks -0.0967217 0.0404623 -2.390 0.016994 *

dog_obedience>3 tasks -0.1499746 0.0388316 -3.862 0.000119 ***

play30m-1h 0.0119406 0.0394914 0.302 0.762434

play1-3h 0.0037299 0.0400093 0.093 0.925740

play>3h -0.0059172 0.0486697 -0.122 0.903255

commands11-30 -0.0068856 0.0272195 -0.253 0.800342

commands>30 -0.0428306 0.0436009 -0.982 0.326149

training2-3 0.0330983 0.0297725 1.112 0.266504

training4 more -0.1046861 0.0342022 -3.061 0.002260 **

dog_beh_changed_3monthYes 0.0536570 0.0262108 2.047 0.040878 *

---

Signif. codes: 0 ‘***’ 0.001 ‘**’ 0.01 ‘*’ 0.05 ‘.’ 0.1 ‘ ’ 1

Residual standard error: 0.3615 on 1118 degrees of freedom

(37 observations deleted due to missingness)

Multiple R-squared: 0.2298, Adjusted R-squared: 0.1947

F-statistic: 6.542 on 51 and 1118 DF, p-value: < 2.2e-16

> Residuals <- residuals (lm)

> qqnorm(Residuals)

> shapiro.test (Residuals)

Shapiro-Wilk normality test

data: Residuals

W = 0.99725, p-value = 0.04155

#### No outliers at 3D or above

### DPQFAC2 – Aggression towards people

#### Age only

> lm<-lm(bcPower(DPQFAC2new,-0.66)~(age), data = data, na.action = na.omit)

> etasq(lm, anova = TRUE, type=3)

Anova Table (Type III tests)

Response: bcPower(DPQFAC2new, -0.66)

Partial eta^2 Sum Sq Df F value Pr(>F)

(Intercept) 0.224265 25.020 1 339.9807 <2e-16 ***

age 0.003733 0.324 5 0.8813 0.4929

Residuals 86.543 1176

---

Signif. codes: 0 ‘***’ 0.001 ‘**’ 0.01 ‘*’ 0.05 ‘.’ 0.1 ‘ ’ 1

> summary(lm)

Call:

lm(formula = bcPower(DPQFAC2new, -0.66) ~ (age), data = data,

na.action = na.omit)

Residuals:

Min 1Q Median 3Q Max

-0.41535 -0.24359 -0.01812 0.22600 0.61752

Coefficients:

Estimate Std. Error t value Pr(>|t|)

(Intercept) 0.373863 0.020276 18.439 <2e-16 ***

ageGroup2 0.040585 0.026674 1.522 0.128

ageGroup3 0.041482 0.028330 1.464 0.143

ageGroup4 0.021575 0.028046 0.769 0.442

ageGroup5 0.000446 0.029096 0.015 0.988

ageGroup6 0.027053 0.027751 0.975 0.330

---

Signif. codes: 0 ‘***’ 0.001 ‘**’ 0.01 ‘*’ 0.05 ‘.’ 0.1 ‘ ’ 1

Residual standard error: 0.2713 on 1176 degrees of freedom

(25 observations deleted due to missingness)

Multiple R-squared: 0.003733, Adjusted R-squared: -0.0005029

F-statistic: 0.8813 on 5 and 1176 DF, p-value: 0.4929

#### Full Model

> lm<-lm(bcPower(DPQFAC2new,-0.66)~(age+sensory+height+weight+breed+sex+neuter+offleash_activity+BCS+food+Add_vitamins+trauma+Health_prob+medication+Owner_age+Owner_dog_exp+How_many_dogs_in_house+How_many_people+Child+Age_of_dog_when_arrived+get_dog+Where_dog_is_kept+dog_obedience+play+commands+training+Time_dog_spend_alone+dog_beh_changed_3month), data = data, na.action = na.omit)

> etasq(lm, anova = TRUE, type=3)

Anova Table (Type III tests)

Response: bcPower(DPQFAC2new, -0.66)

Partial eta^2 Sum Sq Df F value Pr(>F)

(Intercept) 0.036626 2.847 1 42.5422 1.045e-10 ***

age 0.004827 0.363 5 1.0855 0.3665890

sensory 0.001365 0.102 1 1.5293 0.2164841

height 0.000728 0.055 1 0.8151 0.3668135

weight 0.002102 0.158 1 2.3569 0.1250142

breed 0.009620 0.727 1 10.8691 0.0010085 **

sex 0.011958 0.906 1 13.5433 0.0002442 ***

neuter 0.000988 0.074 1 1.1068 0.2930105

offleash_activity 0.002839 0.213 4 0.7966 0.5274288

BCS 0.001040 0.078 2 0.5827 0.5585628

food 0.005374 0.405 4 1.5114 0.1965722

Add_vitamins 0.000610 0.046 3 0.2276 0.8771907

trauma 0.019538 1.492 1 22.2992 2.629e-06 ***

Health_prob 0.001030 0.077 4 0.2883 0.8856681

medication 0.000211 0.016 1 0.2357 0.6274546

Owner_age 0.002368 0.178 3 0.8854 0.4480498

Owner_dog_exp 0.001252 0.094 2 0.7016 0.4960020

How_many_dogs_in_house 0.002552 0.192 2 1.4315 0.2393968

How_many_people 0.003518 0.264 3 1.3169 0.2674034

Child 0.000003 0.000 1 0.0029 0.9568355

Age_of_dog_when_arrived 0.007674 0.579 3 2.8846 0.0347238 *

get_dog 0.002574 0.193 2 1.4438 0.2364721

Where_dog_is_kept 0.003992 0.300 2 2.2426 0.1066565

dog_obedience 0.031860 2.464 3 12.2750 6.636e-08 ***

play 0.002844 0.214 3 1.0640 0.3634281

commands 0.001411 0.106 2 0.7904 0.4539216

training 0.001415 0.106 2 0.7930 0.4527402

Time_dog_spend_alone 0.001976 0.148 3 0.7383 0.5292270

dog_beh_changed_3month 0.001338 0.100 1 1.4989 0.2211025

Residuals 74.875 1119

---

Signif. codes: 0 ‘***’ 0.001 ‘**’ 0.01 ‘*’ 0.05 ‘.’ 0.1 ‘ ’ 1

> summary(lm)

Call:

lm(formula = bcPower(DPQFAC2new, -0.66) ~ (age + sensory +

height + weight + breed + sex + neuter + offleash_activity +

BCS + food + Add_vitamins + trauma + Health_prob + medication +

Owner_age + Owner_dog_exp + How_many_dogs_in_house + How_many_people +

Child + Age_of_dog_when_arrived + get_dog + Where_dog_is_kept +

dog_obedience + play + commands + training + Time_dog_spend_alone +

dog_beh_changed_3month), data = data, na.action = na.omit)

Residuals:

Min 1Q Median 3Q Max

-0.58478 -0.20131 -0.00455 0.19231 0.64776

Coefficients:

Estimate Std. Error t value Pr(>|t|)

(Intercept) 5.075e-01 7.781e-02 6.522 1.05e-10 ***

ageGroup2 2.520e-02 2.690e-02 0.937 0.349022

ageGroup3 1.831e-02 2.877e-02 0.637 0.524536

ageGroup4 -1.643e-03 2.995e-02 -0.055 0.956262

ageGroup5 -2.600e-02 3.166e-02 -0.821 0.411695

ageGroup6 -3.357e-02 3.406e-02 -0.986 0.324478

sensoryPresent -3.075e-02 2.487e-02 -1.237 0.216484

height -6.940e-04 7.687e-04 -0.903 0.366813

weight -1.464e-03 9.537e-04 -1.535 0.125014

breedPure -6.816e-02 2.067e-02 -3.297 0.001009 **

sexMale 6.002e-02 1.631e-02 3.680 0.000244 ***

neuterNeutered -1.851e-02 1.760e-02 -1.052 0.293010

offleash_activity30mins/1hour 1.754e-02 2.713e-02 0.646 0.518133

offleash_activity1/3 9.334e-05 2.624e-02 0.004 0.997162

offleash_activity3/7 -1.561e-02 3.106e-02 -0.502 0.615443

offleash_activity>7 -2.751e-02 3.037e-02 -0.906 0.365204

BCSNormal -1.796e-02 2.163e-02 -0.830 0.406547

BCSOverweight -5.773e-04 2.776e-02 -0.021 0.983415

foodTinned and/or Dry food -1.003e-02 2.832e-02 -0.354 0.723376

foodCooked food 3.952e-02 2.309e-02 1.711 0.087339 .

foodMixed 3.902e-02 2.423e-02 1.610 0.107644

foodRaw 1.878e-02 2.545e-02 0.738 0.460624

Add_vitaminsRarely -1.333e-02 2.039e-02 -0.654 0.513252

Add_vitaminsOften 1.723e-03 2.342e-02 0.074 0.941348

Add_vitaminsDaily -9.052e-03 2.449e-02 -0.370 0.711746

traumaYes 8.221e-02 1.741e-02 4.722 2.63e-06 ***

Health_probTooth problem only 1.839e-02 2.573e-02 0.715 0.475028

Health_probJoint & Tooth probs -1.008e-02 3.096e-02 -0.325 0.744889

Health_probJoint problems only -7.726e-03 2.370e-02 -0.326 0.744471

Health_probOther disorders -1.100e-03 2.550e-02 -0.043 0.965596

medicationYes 1.133e-02 2.333e-02 0.485 0.627455

Owner_age30-39 4.995e-03 2.188e-02 0.228 0.819463

Owner_age40-49 3.025e-02 2.436e-02 1.242 0.214562

Owner_age>50 3.207e-02 2.586e-02 1.240 0.215123

Owner_dog_expHad a dog before 9.348e-03 2.027e-02 0.461 0.644694

Owner_dog_expNever had a dog 2.793e-02 2.436e-02 1.146 0.251923

How_many_dogs_in_houseOne 1.553e-02 1.790e-02 0.868 0.385790

How_many_dogs_in_houseTwo more 3.684e-02 2.182e-02 1.689 0.091557 .

How_many_peopleTwo people -1.197e-02 2.617e-02 -0.458 0.647397

How_many_peopleThree people -1.627e-02 3.166e-02 -0.514 0.607506

How_many_peopleFour more 2.494e-02 3.239e-02 0.770 0.441458

ChildYes -1.256e-03 2.319e-02 -0.054 0.956835

Age_of_dog_when_arrived7-12w 1.658e-02 2.049e-02 0.809 0.418602

Age_of_dog_when_arrived3-12m -7.096e-03 2.613e-02 -0.272 0.786025

Age_of_dog_when_arrived>1y -6.364e-02 2.872e-02 -2.216 0.026921 *

get_dogborn bought 1.195e-02 2.789e-02 0.429 0.668302

get_dogpresent 3.626e-02 2.425e-02 1.495 0.135103

Where_dog_is_keptGarden 9.293e-03 2.750e-02 0.338 0.735537

Where_dog_is_keptApartment -3.390e-02 2.563e-02 -1.323 0.186119

dog_obedience2 tasks -1.390e-03 3.043e-02 -0.046 0.963562

dog_obedience3 tasks -1.191e-01 2.913e-02 -4.091 4.61e-05 ***

dog_obedience>3 tasks -1.256e-01 2.789e-02 -4.503 7.40e-06 ***

play30m-1h -3.656e-02 2.838e-02 -1.288 0.197924

play1-3h -3.775e-02 2.894e-02 -1.305 0.192322

play>3h -7.837e-03 3.515e-02 -0.223 0.823597

commands11-30 2.225e-02 1.945e-02 1.144 0.252967

commands>30 3.647e-03 3.127e-02 0.117 0.907173

training2-3 -2.425e-02 2.132e-02 -1.137 0.255661

training4 more 4.053e-03 2.458e-02 0.165 0.869061

Time_dog_spend_alone1-2h 3.823e-02 2.658e-02 1.438 0.150613

Time_dog_spend_alone3-8h 3.028e-02 2.399e-02 1.262 0.207181

Time_dog_spend_alone>8h 2.503e-02 3.054e-02 0.819 0.412691

dog_beh_changed_3monthYes 2.296e-02 1.875e-02 1.224 0.221103

---

Signif. codes: 0 ‘***’ 0.001 ‘**’ 0.01 ‘*’ 0.05 ‘.’ 0.1 ‘ ’ 1

Residual standard error: 0.2587 on 1119 degrees of freedom

(25 observations deleted due to missingness)

Multiple R-squared: 0.1381, Adjusted R-squared: 0.09029

F-statistic: 2.891 on 62 and 1119 DF, p-value: 4.377e-12

> Residuals <- residuals (lm)

> qqnorm(Residuals)

> shapiro.test (Residuals)

Shapiro-Wilk normality test

data: Residuals

W = 0.9839, p-value = 3.652e-10

#### Reduced Model

> lm<-lm(bcPower(DPQFAC2new,-0.66)~(age+sensory+height+weight+breed+sex+neuter+offleash_activity+BCS+food+Add_vitamins+trauma+Health_prob+medication+Owner_age+Age_of_dog_when_arrived+get_dog+Where_dog_is_kept+dog_obedience+play+commands+training+dog_beh_changed_3month), data = data, na.action = na.omit)

> etasq(lm, anova = TRUE, type=3)

Anova Table (Type III tests)

Response: bcPower(DPQFAC2new, -0.66)

Partial eta^2 Sum Sq Df F value Pr(>F)

(Intercept) 0.057546 4.612 1 68.9981 2.793e-16 ***

age 0.003953 0.300 5 0.8970 0.4822836

sensory 0.001528 0.116 1 1.7293 0.1887715

height 0.000511 0.039 1 0.5778 0.4473468

weight 0.002268 0.172 1 2.5689 0.1092600

breed 0.008869 0.676 1 10.1116 0.0015134 **

sex 0.012571 0.962 1 14.3861 0.0001568 ***

neuter 0.000970 0.073 1 1.0973 0.2950816

offleash_activity 0.002534 0.192 4 0.7175 0.5799772

BCS 0.001225 0.093 2 0.6931 0.5002441

food 0.004842 0.367 4 1.3744 0.2406852

Add_vitamins 0.000505 0.038 3 0.1904 0.9029689

trauma 0.019218 1.480 1 22.1420 2.845e-06 ***

Health_prob 0.001258 0.095 4 0.3559 0.8400439

medication 0.000263 0.020 1 0.2969 0.5859619

Owner_age 0.002726 0.206 3 1.0297 0.3785298

Age_of_dog_when_arrived 0.008174 0.622 3 3.1044 0.0258013 *

get_dog 0.002085 0.158 2 1.1803 0.3075532

Where_dog_is_kept 0.004411 0.335 2 2.5034 0.0822618 .

dog_obedience 0.031266 2.438 3 12.1569 7.822e-08 ***

play 0.002490 0.189 3 0.9404 0.4204168

commands 0.001536 0.116 2 0.8692 0.4195511

training 0.001634 0.124 2 0.9246 0.3969717

dog_beh_changed_3month 0.001194 0.090 1 1.3504 0.2454477

Residuals 75.527 1130

---

Signif. codes: 0 ‘***’ 0.001 ‘**’ 0.01 ‘*’ 0.05 ‘.’ 0.1 ‘ ’ 1

> summary(lm)

Call:

lm(formula = bcPower(DPQFAC2new, -0.66) ~ (age + sensory +

height + weight + breed + sex + neuter + offleash_activity +

BCS + food + Add_vitamins + trauma + Health_prob + medication +

Owner_age + Age_of_dog_when_arrived + get_dog + Where_dog_is_kept +

dog_obedience + play + commands + training + dog_beh_changed_3month),

data = data, na.action = na.omit)

Residuals:

Min 1Q Median 3Q Max

-0.60096 -0.20532 -0.00319 0.19301 0.67084

Coefficients:

Estimate Std. Error t value Pr(>|t|)

(Intercept) 5.575e-01 6.712e-02 8.307 2.79e-16 ***

ageGroup2 2.583e-02 2.671e-02 0.967 0.333844

ageGroup3 2.028e-02 2.867e-02 0.707 0.479480

ageGroup4 4.132e-03 2.966e-02 0.139 0.889250

ageGroup5 -1.947e-02 3.140e-02 -0.620 0.535381

ageGroup6 -2.612e-02 3.375e-02 -0.774 0.439172

sensoryPresent -3.255e-02 2.475e-02 -1.315 0.188771

height -5.802e-04 7.633e-04 -0.760 0.447347

weight -1.520e-03 9.481e-04 -1.603 0.109260

breedPure -6.543e-02 2.058e-02 -3.180 0.001513 **

sexMale 6.132e-02 1.617e-02 3.793 0.000157 ***

neuterNeutered -1.834e-02 1.751e-02 -1.048 0.295082

offleash_activity30mins/1hour 1.834e-02 2.697e-02 0.680 0.496595

offleash_activity1/3 -7.391e-04 2.613e-02 -0.028 0.977437

offleash_activity3/7 -1.047e-02 3.070e-02 -0.341 0.733221

offleash_activity>7 -2.519e-02 3.018e-02 -0.834 0.404224

BCSNormal -2.070e-02 2.134e-02 -0.970 0.332227

BCSOverweight -3.320e-03 2.745e-02 -0.121 0.903753

foodTinned and/or Dry food -8.572e-03 2.802e-02 -0.306 0.759702

foodCooked food 3.835e-02 2.277e-02 1.685 0.092319 .

foodMixed 3.656e-02 2.380e-02 1.537 0.124682

foodRaw 2.132e-02 2.529e-02 0.843 0.399471

Add_vitaminsRarely -1.220e-02 2.021e-02 -0.604 0.546170

Add_vitaminsOften 1.051e-03 2.314e-02 0.045 0.963788

Add_vitaminsDaily -9.126e-03 2.421e-02 -0.377 0.706286

traumaYes 8.143e-02 1.730e-02 4.706 2.85e-06 ***

Health_probTooth problem only 2.061e-02 2.555e-02 0.807 0.420009

Health_probJoint & Tooth probs -1.276e-02 3.074e-02 -0.415 0.678142

Health_probJoint problems only -6.202e-03 2.353e-02 -0.264 0.792183

Health_probOther disorders 7.437e-04 2.528e-02 0.029 0.976540

medicationYes 1.260e-02 2.313e-02 0.545 0.585962

Owner_age30-39 -1.978e-03 2.021e-02 -0.098 0.922074

Owner_age40-49 3.072e-02 2.180e-02 1.409 0.159038

Owner_age>50 2.135e-02 2.339e-02 0.913 0.361651

Age_of_dog_when_arrived7-12w 1.599e-02 2.027e-02 0.789 0.430498

Age_of_dog_when_arrived3-12m -5.703e-03 2.600e-02 -0.219 0.826387

Age_of_dog_when_arrived>1y -6.639e-02 2.864e-02 -2.318 0.020614 *

get_dogborn bought 1.131e-02 2.766e-02 0.409 0.682807

get_dogpresent 3.274e-02 2.403e-02 1.363 0.173269

Where_dog_is_keptGarden 8.126e-03 2.731e-02 0.298 0.766110

Where_dog_is_keptApartment -3.644e-02 2.532e-02 -1.439 0.150411

dog_obedience2 tasks -2.487e-03 3.020e-02 -0.082 0.934368

dog_obedience3 tasks -1.179e-01 2.895e-02 -4.072 4.98e-05 ***

dog_obedience>3 tasks -1.256e-01 2.774e-02 -4.530 6.54e-06 ***

play30m-1h -3.467e-02 2.822e-02 -1.229 0.219465

play1-3h -3.714e-02 2.871e-02 -1.293 0.196117

play>3h -9.976e-03 3.483e-02 -0.286 0.774595

commands11-30 2.211e-02 1.930e-02 1.146 0.252223

commands>30 -9.168e-06 3.085e-02 0.000 0.999763

training2-3 -2.680e-02 2.119e-02 -1.265 0.206185

training4 more 2.076e-03 2.438e-02 0.085 0.932141

dog_beh_changed_3monthYes 2.167e-02 1.865e-02 1.162 0.245448

---

Signif. codes: 0 ‘***’ 0.001 ‘**’ 0.01 ‘*’ 0.05 ‘.’ 0.1 ‘ ’ 1

Residual standard error: 0.2585 on 1130 degrees of freedom

(25 observations deleted due to missingness)

Multiple R-squared: 0.1305, Adjusted R-squared: 0.0913

F-statistic: 3.327 on 51 and 1130 DF, p-value: 2.13e-13

> Residuals <- residuals (lm)

> qqnorm(Residuals)

> shapiro.test (Residuals)

Shapiro-Wilk normality test

data: Residuals

W = 0.98401, p-value = 4.081e-10

#### Outliers removed N = 1166 (16 outliers removed)

> lm<-lm(bcPower(outDPQFAC2new,-0.66)~(age+sensory+height+weight+breed+sex+neuter+offleash_activity+BCS+food+Add_vitamins+trauma+Health_prob+medication+Owner_age+Age_of_dog_when_arrived+get_dog+Where_dog_is_kept+dog_obedience+play+commands+training+dog_beh_changed_3month), data = data, na.action = na.omit)

> etasq(lm, anova = TRUE, type=3)

Anova Table (Type III tests)

Response: bcPower(outDPQFAC2new, -0.66)

Partial eta^2 Sum Sq Df F value Pr(>F)

(Intercept) 0.052751 3.977 1 62.0374 7.962e-15 ***

age 0.003949 0.283 5 0.8832 0.4915656

sensory 0.001543 0.110 1 1.7213 0.1897976

height 0.000602 0.043 1 0.6710 0.4128699

weight 0.002915 0.209 1 3.2568 0.0713964 .

breed 0.007616 0.548 1 8.5495 0.0035260 **

sex 0.012703 0.919 1 14.3331 0.0001613 ***

neuter 0.000944 0.067 1 1.0521 0.3052398

offleash_activity 0.002758 0.197 4 0.7702 0.5446747

BCS 0.001678 0.120 2 0.9361 0.3924497

food 0.005960 0.428 4 1.6699 0.1546523

Add_vitamins 0.000391 0.028 3 0.1452 0.9327411

trauma 0.018203 1.324 1 20.6541 6.102e-06 ***

Health_prob 0.001328 0.095 4 0.3703 0.8298985

medication 0.000139 0.010 1 0.1544 0.6944261

Owner_age 0.004538 0.326 3 1.6927 0.1668249

Age_of_dog_when_arrived 0.007614 0.548 3 2.8489 0.0364351 *

get_dog 0.001775 0.127 2 0.9905 0.3717044

Where_dog_is_kept 0.005515 0.396 2 3.0891 0.0459341 *

dog_obedience 0.028452 2.091 3 10.8746 4.820e-07 ***

play 0.002971 0.213 3 1.1067 0.3453359

commands 0.001194 0.085 2 0.6658 0.5140884

training 0.001557 0.111 2 0.8689 0.4197142

dog_beh_changed_3month 0.000592 0.042 1 0.6603 0.4166268

Residuals 71.408 1114

---

Signif. codes: 0 ‘***’ 0.001 ‘**’ 0.01 ‘*’ 0.05 ‘.’ 0.1 ‘ ’ 1

> summary(lm)

Call:

lm(formula = bcPower(outDPQFAC2new, -0.66) ~ (age + sensory +

height + weight + breed + sex + neuter + offleash_activity +

BCS + food + Add_vitamins + trauma + Health_prob + medication +

Owner_age + Age_of_dog_when_arrived + get_dog + Where_dog_is_kept +

dog_obedience + play + commands + training + dog_beh_changed_3month),

data = data, na.action = na.omit)

Residuals:

Min 1Q Median 3Q Max

-0.59885 -0.20329 0.00115 0.19292 0.62547

Coefficients:

Estimate Std. Error t value Pr(>|t|)

(Intercept) 5.232e-01 6.642e-02 7.876 7.96e-15 ***

ageGroup2 3.304e-02 2.654e-02 1.245 0.213466

ageGroup3 2.406e-02 2.842e-02 0.847 0.397422

ageGroup4 1.888e-02 2.942e-02 0.642 0.521300

ageGroup5 -1.016e-02 3.115e-02 -0.326 0.744307

ageGroup6 -1.386e-02 3.363e-02 -0.412 0.680264

sensoryPresent -3.206e-02 2.444e-02 -1.312 0.189798

height -6.230e-04 7.605e-04 -0.819 0.412870

weight -1.698e-03 9.410e-04 -1.805 0.071396 .

breedPure -5.949e-02 2.035e-02 -2.924 0.003526 **

sexMale 6.040e-02 1.596e-02 3.786 0.000161 ***

neuterNeutered -1.770e-02 1.726e-02 -1.026 0.305240

offleash_activity30mins/1hour 2.188e-02 2.674e-02 0.818 0.413335

offleash_activity1/3 3.336e-03 2.582e-02 0.129 0.897209

offleash_activity3/7 -9.958e-03 3.039e-02 -0.328 0.743238

offleash_activity>7 -2.147e-02 2.987e-02 -0.719 0.472507

BCSNormal -1.821e-02 2.115e-02 -0.861 0.389287

BCSOverweight 6.676e-03 2.712e-02 0.246 0.805628

foodTinned and/or Dry food -1.430e-02 2.764e-02 -0.517 0.604990

foodCooked food 3.764e-02 2.243e-02 1.678 0.093564 .

foodMixed 3.932e-02 2.347e-02 1.676 0.094073 .

foodRaw 2.449e-02 2.492e-02 0.983 0.325947

Add_vitaminsRarely 6.465e-06 2.001e-02 0.000 0.999742

Add_vitaminsOften 1.284e-02 2.288e-02 0.561 0.574963

Add_vitaminsDaily 1.252e-03 2.396e-02 0.052 0.958332

traumaYes 7.756e-02 1.707e-02 4.545 6.10e-06 ***

Health_probTooth problem only 1.714e-02 2.517e-02 0.681 0.496105

Health_probJoint & Tooth probs -1.478e-02 3.020e-02 -0.489 0.624762

Health_probJoint problems only -1.113e-02 2.327e-02 -0.478 0.632508

Health_probOther disorders -4.846e-03 2.498e-02 -0.194 0.846221

medicationYes 8.968e-03 2.282e-02 0.393 0.694426

Owner_age30-39 6.311e-03 2.000e-02 0.316 0.752408

Owner_age40-49 4.405e-02 2.147e-02 2.052 0.040422 *

Owner_age>50 2.730e-02 2.314e-02 1.180 0.238339

Age_of_dog_when_arrived7-12w 2.090e-02 2.005e-02 1.043 0.297334

Age_of_dog_when_arrived3-12m 1.365e-03 2.569e-02 0.053 0.957639

Age_of_dog_when_arrived>1y -5.624e-02 2.828e-02 -1.989 0.046953 *

get_dogborn bought 1.128e-02 2.727e-02 0.414 0.679275

get_dogpresent 3.004e-02 2.366e-02 1.269 0.204570

Where_dog_is_keptGarden 1.173e-02 2.694e-02 0.435 0.663365

Where_dog_is_keptApartment -3.783e-02 2.501e-02 -1.513 0.130659

dog_obedience2 tasks -1.664e-02 3.003e-02 -0.554 0.579684

dog_obedience3 tasks -1.189e-01 2.862e-02 -4.154 3.52e-05 ***

dog_obedience>3 tasks -1.251e-01 2.744e-02 -4.558 5.74e-06 ***

play30m-1h -3.656e-02 2.786e-02 -1.312 0.189632

play1-3h -3.254e-02 2.835e-02 -1.148 0.251393

play>3h -2.597e-03 3.439e-02 -0.076 0.939807

commands11-30 1.880e-02 1.907e-02 0.986 0.324406

commands>30 -8.784e-04 3.029e-02 -0.029 0.976874

training2-3 -2.214e-02 2.093e-02 -1.058 0.290370

training4 more 1.003e-02 2.393e-02 0.419 0.675233

dog_beh_changed_3monthYes 1.501e-02 1.848e-02 0.813 0.416627

---

Signif. codes: 0 ‘***’ 0.001 ‘**’ 0.01 ‘*’ 0.05 ‘.’ 0.1 ‘ ’ 1

Residual standard error: 0.2532 on 1114 degrees of freedom

(41 observations deleted due to missingness)

Multiple R-squared: 0.128, Adjusted R-squared: 0.08807

F-statistic: 3.206 on 51 and 1114 DF, p-value: 1.482e-12

> Residuals <- residuals (lm)

> qqnorm(Residuals)

> shapiro.test (Residuals)

Shapiro-Wilk normality test

data: Residuals

W = 0.98324, p-value = 2.404e-10

### DPQFAC3 – Activity/Excitability

#### Age only

> lm<-lm(bcPower(DPQFAC3new,1.59)~(age), data = data, na.action = na.omit)

> etasq(lm, anova = TRUE, type=3)

Anova Table (Type III tests)

Response: bcPower(DPQFAC3new, 1.59)

Partial eta^2 Sum Sq Df F value Pr(>F)

(Intercept) 0.73934 4430.2 1 3261.861 < 2.2e-16 ***

age 0.18247 348.6 5 51.337 < 2.2e-16 ***

Residuals 1561.9 1150

---

Signif. codes: 0 ‘***’ 0.001 ‘**’ 0.01 ‘*’ 0.05 ‘.’ 0.1 ‘ ’ 1

> summary(lm)

Call:

lm(formula = bcPower(DPQFAC3new, 1.59) ~ (age), data = data,

na.action = na.omit)

Residuals:

Min 1Q Median 3Q Max

-3.9690 -0.8261 0.0601 0.8153 4.0475

Coefficients:

Estimate Std. Error t value Pr(>|t|)

(Intercept) 4.98884 0.08735 57.113 < 2e-16 ***

ageGroup2 -0.55584 0.11518 -4.826 1.58e-06 ***

ageGroup3 -0.86016 0.12285 -7.002 4.29e-12 ***

ageGroup4 -1.06816 0.12126 -8.809 < 2e-16 ***

ageGroup5 -1.28892 0.12655 -10.185 < 2e-16 ***

ageGroup6 -1.75197 0.11995 -14.606 < 2e-16 ***

---

Signif. codes: 0 ‘***’ 0.001 ‘**’ 0.01 ‘*’ 0.05 ‘.’ 0.1 ‘ ’ 1

Residual standard error: 1.165 on 1150 degrees of freedom

(51 observations deleted due to missingness)

Multiple R-squared: 0.1825, Adjusted R-squared: 0.1789

F-statistic: 51.34 on 5 and 1150 DF, p-value: < 2.2e-16

> Residuals <- residuals (lm)

> qqnorm(Residuals)

> shapiro.test (Residuals)

Shapiro-Wilk normality test

data: Residuals

W = 0.99788, p-value = 0.146

| > com=glht(lm,linfct=mcp(age="Tukey"))  > summary(com)  Simultaneous Tests for General Linear Hypotheses  Multiple Comparisons of Means: Tukey Contrasts  Fit: lm(formula = bcPower(DPQFAC3new, 1.59) ~ (age), data = data,  na.action = na.omit)  Linear Hypotheses:  Estimate Std. Error t value Pr(>\|t\|)  Group2 - Group1 == 0 -0.5558 0.1152 -4.826 < 0.001 ***  Group3 - Group1 == 0 -0.8602 0.1229 -7.002 < 0.001 ***  Group4 - Group1 == 0 -1.0682 0.1213 -8.809 < 0.001 ***  Group5 - Group1 == 0 -1.2889 0.1265 -10.185 < 0.001 ***  Group6 - Group1 == 0 -1.7520 0.1199 -14.606 < 0.001 ***  Group3 - Group2 == 0 -0.3043 0.1144 -2.659 0.08409 .  Group4 - Group2 == 0 -0.5123 0.1127 -4.544 < 0.001 ***  Group5 - Group2 == 0 -0.7331 0.1184 -6.191 < 0.001 ***  Group6 - Group2 == 0 -1.1961 0.1113 -10.745 < 0.001 ***  Group4 - Group3 == 0 -0.2080 0.1206 -1.725 0.51461  Group5 - Group3 == 0 -0.4288 0.1259 -3.406 0.00884 **  Group6 - Group3 == 0 -0.8918 0.1192 -7.479 < 0.001 ***  Group5 - Group4 == 0 -0.2208 0.1243 -1.776 0.48124  Group6 - Group4 == 0 -0.6838 0.1176 -5.815 < 0.001 ***  Group6 - Group5 == 0 -0.4631 0.1230 -3.763 0.00250 **  ---  Signif. codes: 0 ‘***’ 0.001 ‘**’ 0.01 ‘*’ 0.05 ‘.’ 0.1 ‘ ’ 1  (Adjusted p values reported -- single-step method) |
| --- |
|  |
| \|  \| \| --- \| |

#### Full Model

> lm<-lm(bcPower(DPQFAC3new,1.51)~(age+sensory+height+weight+breed+sex+neuter+offleash_activity+BCS+food+Add_vitamins+trauma+Health_prob+medication+Owner_age+Owner_dog_exp+How_many_dogs_in_house+How_many_people+Child+Age_of_dog_when_arrived+get_dog+Where_dog_is_kept+dog_obedience+play+commands+training+Time_dog_spend_alone+dog_beh_changed_3month), data = data, na.action = na.omit)

> etasq(lm, anova = TRUE, type=3)

Anova Table (Type III tests)

Response: bcPower(DPQFAC3new, 1.51)

Partial eta^2 Sum Sq Df F value Pr(>F)

(Intercept) 0.169072 211.80 1 222.3973 < 2.2e-16 ***

age 0.074390 83.66 5 17.5685 < 2.2e-16 ***

sensory 0.005915 6.19 1 6.5032 0.010904 *

height 0.000030 0.03 1 0.0332 0.855383

weight 0.001106 1.15 1 1.2100 0.271579

breed 0.003239 3.38 1 3.5517 0.059750 .

sex 0.003278 3.42 1 3.5948 0.058224 .

neuter 0.000031 0.03 1 0.0334 0.855018

offleash_activity 0.007675 8.05 4 2.1133 0.077082 .

BCS 0.009753 10.25 2 5.3824 0.004720 **

food 0.004945 5.17 4 1.3579 0.246575

Add_vitamins 0.005371 5.62 3 1.9672 0.117164

trauma 0.001493 1.56 1 1.6343 0.201380

Health_prob 0.003746 3.91 4 1.0273 0.391934

medication 0.001553 1.62 1 1.7005 0.192497

Owner_age 0.002554 2.67 3 0.9330 0.424041

Owner_dog_exp 0.001990 2.08 2 1.0900 0.336589

How_many_dogs_in_house 0.001175 1.23 2 0.6431 0.525842

How_many_people 0.000871 0.91 3 0.3176 0.812653

Child 0.000393 0.41 1 0.4293 0.512493

Age_of_dog_when_arrived 0.037269 40.30 3 14.1039 5.042e-09 ***

get_dog 0.008831 9.27 2 4.8693 0.007846 **

Where_dog_is_kept 0.001951 2.04 2 1.0686 0.343858

dog_obedience 0.033851 36.47 3 12.7651 3.341e-08 ***

play 0.005261 5.51 3 1.9269 0.123462

commands 0.005860 6.14 2 3.2212 0.040287 *

training 0.002250 2.35 2 1.2326 0.291934

Time_dog_spend_alone 0.000763 0.79 3 0.2781 0.841197

dog_beh_changed_3month 0.000044 0.05 1 0.0479 0.826799

Residuals 1040.94 1093

---

Signif. codes: 0 ‘***’ 0.001 ‘**’ 0.01 ‘*’ 0.05 ‘.’ 0.1 ‘ ’ 1

> summary(lm)

Call:

lm(formula = bcPower(DPQFAC3new, 1.51) ~ (age + sensory + height +

weight + breed + sex + neuter + offleash_activity + BCS +

food + Add_vitamins + trauma + Health_prob + medication +

Owner_age + Owner_dog_exp + How_many_dogs_in_house + How_many_people +

Child + Age_of_dog_when_arrived + get_dog + Where_dog_is_kept +

dog_obedience + play + commands + training + Time_dog_spend_alone +

dog_beh_changed_3month), data = data, na.action = na.omit)

Residuals:

Min 1Q Median 3Q Max

-3.6323 -0.6209 0.0255 0.6255 3.4468

Coefficients:

Estimate Std. Error t value Pr(>|t|)

(Intercept) 4.4563832 0.2988256 14.913 < 2e-16 ***

ageGroup2 -0.3919530 0.1021731 -3.836 0.000132 ***

ageGroup3 -0.6601073 0.1096132 -6.022 2.35e-09 ***

ageGroup4 -0.7544570 0.1144998 -6.589 6.86e-11 ***

ageGroup5 -0.9085286 0.1213981 -7.484 1.48e-13 ***

ageGroup6 -1.0835702 0.1296519 -8.358 < 2e-16 ***

sensoryPresent -0.2416198 0.0947478 -2.550 0.010904 *

height -0.0005333 0.0029254 -0.182 0.855383

weight -0.0039644 0.0036040 -1.100 0.271579

breedPure 0.1485378 0.0788169 1.885 0.059750 .

sexMale 0.1179781 0.0622250 1.896 0.058224 .

neuterNeutered 0.0123141 0.0673777 0.183 0.855018

offleash_activity30mins/1hour -0.0169287 0.1038273 -0.163 0.870512

offleash_activity1/3 0.0824816 0.0999533 0.825 0.409437

offleash_activity3/7 0.1989220 0.1180553 1.685 0.092276 .

offleash_activity>7 0.2356478 0.1154445 2.041 0.041469 *

BCSNormal -0.0005432 0.0823522 -0.007 0.994738

BCSOverweight -0.2630541 0.1055342 -2.493 0.012829 *

foodTinned and/or Dry food -0.1765210 0.1094041 -1.613 0.106930

foodCooked food -0.0678110 0.0888180 -0.763 0.445340

foodMixed -0.0788366 0.0928779 -0.849 0.396168

foodRaw -0.2001722 0.0977710 -2.047 0.040862 *

Add_vitaminsRarely -0.0632651 0.0780424 -0.811 0.417743

Add_vitaminsOften 0.1385390 0.0897279 1.544 0.122880

Add_vitaminsDaily 0.0487249 0.0937762 0.520 0.603456

traumaYes -0.0859092 0.0672006 -1.278 0.201380

Health_probTooth problem only -0.0280801 0.0989661 -0.284 0.776667

Health_probJoint & Tooth probs -0.2237856 0.1181688 -1.894 0.058518 .

Health_probJoint problems only -0.0334968 0.0906248 -0.370 0.711737

Health_probOther disorders -0.0793772 0.0986042 -0.805 0.420990

medicationYes -0.1162779 0.0891680 -1.304 0.192497

Owner_age30-39 -0.1082484 0.0838418 -1.291 0.196941

Owner_age40-49 0.0071642 0.0932533 0.077 0.938777

Owner_age>50 -0.0885826 0.0988123 -0.896 0.370197

Owner_dog_expHad a dog before 0.0360795 0.0773715 0.466 0.641083

Owner_dog_expNever had a dog -0.0771198 0.0933142 -0.826 0.408728

How_many_dogs_in_houseOne -0.0483356 0.0682830 -0.708 0.479176

How_many_dogs_in_houseTwo more -0.0924617 0.0831515 -1.112 0.266397

How_many_peopleTwo people 0.0897112 0.0997384 0.899 0.368603

How_many_peopleThree people 0.0744624 0.1207552 0.617 0.537601

How_many_peopleFour more 0.0469381 0.1237845 0.379 0.704619

ChildYes 0.0581254 0.0887175 0.655 0.512493

Age_of_dog_when_arrived7-12w -0.2135075 0.0786436 -2.715 0.006735 **

Age_of_dog_when_arrived3-12m -0.4796543 0.0998467 -4.804 1.77e-06 ***

Age_of_dog_when_arrived>1y -0.6718165 0.1095842 -6.131 1.22e-09 ***

get_dogborn bought -0.3316150 0.1065334 -3.113 0.001901 **

get_dogpresent -0.1823462 0.0927892 -1.965 0.049648 *

Where_dog_is_keptGarden -0.0687641 0.1052142 -0.654 0.513531

Where_dog_is_keptApartment -0.1386509 0.0985523 -1.407 0.159749

dog_obedience2 tasks 0.2923462 0.1151357 2.539 0.011250 *

dog_obedience3 tasks 0.3192787 0.1111397 2.873 0.004148 **

dog_obedience>3 tasks 0.6188376 0.1056294 5.859 6.17e-09 ***

play30m-1h 0.2433437 0.1076485 2.261 0.023984 *

play1-3h 0.1493527 0.1096182 1.362 0.173327

play>3h 0.1252774 0.1343945 0.932 0.351459

commands11-30 0.0827449 0.0742818 1.114 0.265553

commands>30 0.3039311 0.1199926 2.533 0.011451 *

training2-3 -0.0771670 0.0810154 -0.952 0.341056

training4 more 0.0843168 0.0937302 0.900 0.368548

Time_dog_spend_alone1-2h 0.0704978 0.1021305 0.690 0.490169

Time_dog_spend_alone3-8h 0.0173918 0.0917693 0.190 0.849723

Time_dog_spend_alone>8h -0.0101965 0.1174581 -0.087 0.930839

dog_beh_changed_3monthYes -0.0157364 0.0719011 -0.219 0.826799

---

Signif. codes: 0 ‘***’ 0.001 ‘**’ 0.01 ‘*’ 0.05 ‘.’ 0.1 ‘ ’ 1

Residual standard error: 0.9759 on 1093 degrees of freedom

(51 observations deleted due to missingness)

Multiple R-squared: 0.3363, Adjusted R-squared: 0.2987

F-statistic: 8.935 on 62 and 1093 DF, p-value: < 2.2e-16

> Residuals <- residuals (lm)

> qqnorm(Residuals)

> shapiro.test (Residuals)

Shapiro-Wilk normality test

data: Residuals

W = 0.99755, p-value = 0.07853

#### Reduced Model

> lm<-lm(bcPower(DPQFAC3new,1.51)~(age+sensory+height+weight+breed+sex+neuter+offleash_activity+BCS+food+Add_vitamins+trauma+Health_prob+medication+Owner_age+Age_of_dog_when_arrived+get_dog+Where_dog_is_kept+dog_obedience+play+commands+training+dog_beh_changed_3month), data = data, na.action = na.omit)

> etasq(lm, anova = TRUE, type=3)

Anova Table (Type III tests)

Response: bcPower(DPQFAC3new, 1.51)

Partial eta^2 Sum Sq Df F value Pr(>F)

(Intercept) 0.221513 297.75 1 314.1355 < 2.2e-16 ***

age 0.075970 86.03 5 18.1532 < 2.2e-16 ***

sensory 0.005877 6.19 1 6.5261 0.010764 *

height 0.000124 0.13 1 0.1372 0.711144

weight 0.000849 0.89 1 0.9380 0.332994

breed 0.003058 3.21 1 3.3866 0.065995 .

sex 0.003435 3.61 1 3.8057 0.051332 .

neuter 0.000000 0.00 1 0.0003 0.985089

offleash_activity 0.007332 7.73 4 2.0386 0.086862 .

BCS 0.009736 10.29 2 5.4270 0.004514 **

food 0.004888 5.14 4 1.3556 0.247383

Add_vitamins 0.005239 5.51 3 1.9379 0.121693

trauma 0.001464 1.53 1 1.6190 0.203503

Health_prob 0.004418 4.64 4 1.2248 0.298492

medication 0.001559 1.63 1 1.7237 0.189494

Owner_age 0.002546 2.67 3 0.9394 0.420880

Age_of_dog_when_arrived 0.037211 40.44 3 14.2231 4.246e-09 ***

get_dog 0.008879 9.37 2 4.9451 0.007277 **

Where_dog_is_kept 0.001926 2.02 2 1.0654 0.344936

dog_obedience 0.033457 36.22 3 12.7383 3.460e-08 ***

play 0.005220 5.49 3 1.9309 0.122808

commands 0.006101 6.42 2 3.3884 0.034114 *

training 0.002219 2.33 2 1.2278 0.293346

dog_beh_changed_3month 0.000062 0.06 1 0.0684 0.793801

Residuals 1046.41 1104

---

Signif. codes: 0 ‘***’ 0.001 ‘**’ 0.01 ‘*’ 0.05 ‘.’ 0.1 ‘ ’ 1

> summary(lm)

Call:

lm(formula = bcPower(DPQFAC3new, 1.51) ~ (age + sensory + height +

weight + breed + sex + neuter + offleash_activity + BCS +

food + Add_vitamins + trauma + Health_prob + medication +

Owner_age + Age_of_dog_when_arrived + get_dog + Where_dog_is_kept +

dog_obedience + play + commands + training + dog_beh_changed_3month),

data = data, na.action = na.omit)

Residuals:

Min 1Q Median 3Q Max

-3.6446 -0.6414 0.0243 0.6305 3.4528

Coefficients:

Estimate Std. Error t value Pr(>|t|)

(Intercept) 4.542784 0.256309 17.724 < 2e-16 ***

ageGroup2 -0.393857 0.101396 -3.884 0.000109 ***

ageGroup3 -0.660767 0.109066 -6.058 1.88e-09 ***

ageGroup4 -0.764156 0.113181 -6.752 2.36e-11 ***

ageGroup5 -0.918138 0.120399 -7.626 5.22e-14 ***

ageGroup6 -1.089676 0.128602 -8.473 < 2e-16 ***

sensoryPresent -0.240414 0.094110 -2.555 0.010764 *

height -0.001074 0.002900 -0.370 0.711144

weight -0.003462 0.003574 -0.969 0.332994

breedPure 0.143972 0.078233 1.840 0.065995 .

sexMale 0.120257 0.061644 1.951 0.051332 .

neuterNeutered 0.001250 0.066842 0.019 0.985089

offleash_activity30mins/1hour -0.025357 0.102956 -0.246 0.805507

offleash_activity1/3 0.083642 0.099321 0.842 0.399890

offleash_activity3/7 0.187261 0.116509 1.607 0.108281

offleash_activity>7 0.223047 0.114449 1.949 0.051564 .

BCSNormal -0.003058 0.081290 -0.038 0.969998

BCSOverweight -0.264366 0.104303 -2.535 0.011395 *

foodTinned and/or Dry food -0.161651 0.107939 -1.498 0.134519

foodCooked food -0.057153 0.087438 -0.654 0.513482

foodMixed -0.063948 0.090933 -0.703 0.482055

foodRaw -0.199051 0.097003 -2.052 0.040404 *

Add_vitaminsRarely -0.073105 0.077153 -0.948 0.343574

Add_vitaminsOften 0.127625 0.088375 1.444 0.148985

Add_vitaminsDaily 0.032663 0.092375 0.354 0.723711

traumaYes -0.084840 0.066678 -1.272 0.203503

Health_probTooth problem only -0.027681 0.097928 -0.283 0.777486

Health_probJoint & Tooth probs -0.238089 0.117014 -2.035 0.042120 *

Health_probJoint problems only -0.039292 0.089827 -0.437 0.661891

Health_probOther disorders -0.098853 0.097549 -1.013 0.311106

medicationYes -0.115718 0.088140 -1.313 0.189494

Owner_age30-39 -0.095092 0.077125 -1.233 0.217854

Owner_age40-49 0.027330 0.083316 0.328 0.742951

Owner_age>50 -0.068633 0.089133 -0.770 0.441457

Age_of_dog_when_arrived7-12w -0.208057 0.077713 -2.677 0.007533 **

Age_of_dog_when_arrived3-12m -0.474382 0.099213 -4.781 1.98e-06 ***

Age_of_dog_when_arrived>1y -0.672949 0.109039 -6.172 9.48e-10 ***

get_dogborn bought -0.329687 0.105449 -3.127 0.001815 **

get_dogpresent -0.173388 0.091738 -1.890 0.059016 .

Where_dog_is_keptGarden -0.066171 0.104258 -0.635 0.525761

Where_dog_is_keptApartment -0.135907 0.097188 -1.398 0.162276

dog_obedience2 tasks 0.302246 0.114082 2.649 0.008179 **

dog_obedience3 tasks 0.321454 0.110164 2.918 0.003595 **

dog_obedience>3 tasks 0.617234 0.104827 5.888 5.18e-09 ***

play30m-1h 0.238903 0.106737 2.238 0.025405 *

play1-3h 0.140138 0.108460 1.292 0.196604

play>3h 0.118006 0.132789 0.889 0.374373

commands11-30 0.088798 0.073600 1.206 0.227885

commands>30 0.307511 0.118197 2.602 0.009401 **

training2-3 -0.080240 0.080328 -0.999 0.318062

training4 more 0.078302 0.092814 0.844 0.399051

dog_beh_changed_3monthYes -0.018673 0.071421 -0.261 0.793801

---

Signif. codes: 0 ‘***’ 0.001 ‘**’ 0.01 ‘*’ 0.05 ‘.’ 0.1 ‘ ’ 1

Residual standard error: 0.9736 on 1104 degrees of freedom

(51 observations deleted due to missingness)

Multiple R-squared: 0.3329, Adjusted R-squared: 0.302

F-statistic: 10.8 on 51 and 1104 DF, p-value: < 2.2e-16

> Residuals <- residuals (lm)

> qqnorm(Residuals)

> shapiro.test (Residuals)

Shapiro-Wilk normality test

data: Residuals

W = 0.99718, p-value = 0.03878

> new_cog_no_missing3$shape <- as.factor(new_cog_no_missing3$shape)

> com=glht(lm,linfct=mcp(shape="Tukey")

+ )

> summary(com)

Simultaneous Tests for General Linear Hypotheses

Multiple Comparisons of Means: Tukey Contrasts

Fit: lm(formula = bcPower(DPQFAC3new, 1.51) ~ (age3 + sensory2 + height +

weight + breed + sex + neuter2 + offleash_activity + shape +

food + Add_vitamins + trauma + Health_prob + medication +

Owner_age + Age_of_dog_when_arrived + get_dog + Where_dog_is_kept +

dog_jobs + play + commands + training + dog_beh_changed_3month),

data = new_cog_no_missing3, na.action = na.omit)

Linear Hypotheses:

Estimate Std. Error t value Pr(>|t|)

Normal - Thin == 0 -0.003058 0.081290 -0.038 0.99920

Overweight – Thin == 0 -0.264366 0.104303 -2.535 0.02967 *

Overweight - Normal == 0 -0.261308 0.080657 -3.240 0.00359 **

---

Signif. codes: 0 ‘***’ 0.001 ‘**’ 0.01 ‘*’ 0.05 ‘.’ 0.1 ‘ ’ 1

(Adjusted p values reported -- single-step method)

#### Outliers removed (3 removed)

> lm<-lm(bcPower(DPQFAC3new,1.51)~(age+sensory+height+weight+breed+sex+neuter+offleash_activity+BCS+food+Add_vitamins+trauma+Health_prob+medication+Owner_age+Age_of_dog_when_arrived+get_dog+Where_dog_is_kept+dog_obedience+play+commands+training+dog_beh_changed_3month), data = data, na.action = na.omit)

> etasq(lm, anova = TRUE, type=3)

Anova Table (Type III tests)

Response: bcPower(DPQFAC3new, 1.51)

Partial eta^2 Sum Sq Df F value Pr(>F)

(Intercept) 0.221513 297.75 1 314.1355 < 2.2e-16 ***

age 0.075970 86.03 5 18.1532 < 2.2e-16 ***

sensory 0.005877 6.19 1 6.5261 0.010764 *

height 0.000124 0.13 1 0.1372 0.711144

weight 0.000849 0.89 1 0.9380 0.332994

breed 0.003058 3.21 1 3.3866 0.065995 .

sex 0.003435 3.61 1 3.8057 0.051332 .

neuter 0.000000 0.00 1 0.0003 0.985089

offleash_activity 0.007332 7.73 4 2.0386 0.086862 .

BCS 0.009736 10.29 2 5.4270 0.004514 **

food 0.004888 5.14 4 1.3556 0.247383

Add_vitamins 0.005239 5.51 3 1.9379 0.121693

trauma 0.001464 1.53 1 1.6190 0.203503

Health_prob 0.004418 4.64 4 1.2248 0.298492

medication 0.001559 1.63 1 1.7237 0.189494

Owner_age 0.002546 2.67 3 0.9394 0.420880

Age_of_dog_when_arrived 0.037211 40.44 3 14.2231 4.246e-09 ***

get_dog 0.008879 9.37 2 4.9451 0.007277 **

Where_dog_is_kept 0.001926 2.02 2 1.0654 0.344936

dog_obedience 0.033457 36.22 3 12.7383 3.460e-08 ***

play 0.005220 5.49 3 1.9309 0.122808

commands 0.006101 6.42 2 3.3884 0.034114 *

training 0.002219 2.33 2 1.2278 0.293346

dog_beh_changed_3month 0.000062 0.06 1 0.0684 0.793801

Residuals 1046.41 1104

---

Signif. codes: 0 ‘***’ 0.001 ‘**’ 0.01 ‘*’ 0.05 ‘.’ 0.1 ‘ ’ 1

> summary(lm)

Call:

lm(formula = bcPower(DPQFAC3new, 1.51) ~ (age + sensory + height +

weight + breed + sex + neuter + offleash_activity + BCS +

food + Add_vitamins + trauma + Health_prob + medication +

Owner_age + Age_of_dog_when_arrived + get_dog + Where_dog_is_kept +

dog_obedience + play + commands + training + dog_beh_changed_3month),

data = data, na.action = na.omit)

Residuals:

Min 1Q Median 3Q Max

-3.6446 -0.6414 0.0243 0.6305 3.4528

Coefficients:

Estimate Std. Error t value Pr(>|t|)

(Intercept) 4.542784 0.256309 17.724 < 2e-16 ***

ageGroup2 -0.393857 0.101396 -3.884 0.000109 ***

ageGroup3 -0.660767 0.109066 -6.058 1.88e-09 ***

ageGroup4 -0.764156 0.113181 -6.752 2.36e-11 ***

ageGroup5 -0.918138 0.120399 -7.626 5.22e-14 ***

ageGroup6 -1.089676 0.128602 -8.473 < 2e-16 ***

sensoryPresent -0.240414 0.094110 -2.555 0.010764 *

height -0.001074 0.002900 -0.370 0.711144

weight -0.003462 0.003574 -0.969 0.332994

breedPure 0.143972 0.078233 1.840 0.065995 .

sexMale 0.120257 0.061644 1.951 0.051332 .

neuterNeutered 0.001250 0.066842 0.019 0.985089

offleash_activity30mins/1hour 0.025357 0.102956 -0.246 0.805507

offleash_activity1/3 0.083642 0.099321 0.842 0.399890

offleash_activity3/7 0.187261 0.116509 1.607 0.108281

offleash_activity>7 0.223047 0.114449 1.949 0.051564 .

BCSNormal -0.003058 0.081290 -0.038 0.969998

BCSOverweight -0.264366 0.104303 -2.535 0.011395 *

foodTinned and/or Dry food -0.161651 0.107939 -1.498 0.134519

foodCooked food -0.057153 0.087438 -0.654 0.513482

foodMixed -0.063948 0.090933 -0.703 0.482055

foodRaw -0.199051 0.097003 -2.052 0.040404 *

Add_vitaminsRarely -0.073105 0.077153 -0.948 0.343574

Add_vitaminsOften 0.127625 0.088375 1.444 0.148985

Add_vitaminsDaily 0.032663 0.092375 0.354 0.723711

traumaYes -0.084840 0.066678 -1.272 0.203503

Health_probTooth problem only -0.027681 0.097928 -0.283 0.777486

Health_probJoint & Tooth probs -0.238089 0.117014 -2.035 0.042120 *

Health_probJoint problems only -0.039292 0.089827 -0.437 0.661891

Health_probOther disorders -0.098853 0.097549 -1.013 0.311106

medicationYes -0.115718 0.088140 -1.313 0.189494

Owner_age30-39 -0.095092 0.077125 -1.233 0.217854

Owner_age40-49 0.027330 0.083316 0.328 0.742951

Owner_age>50 -0.068633 0.089133 -0.770 0.441457

Age_of_dog_when_arrived7-12w -0.208057 0.077713 -2.677 0.007533 **

Age_of_dog_when_arrived3-12m -0.474382 0.099213 -4.781 1.98e-06 ***

Age_of_dog_when_arrived>1y -0.672949 0.109039 -6.172 9.48e-10 ***

get_dogborn bought -0.329687 0.105449 -3.127 0.001815 **

get_dogpresent -0.173388 0.091738 -1.890 0.059016 .

Where_dog_is_keptGarden -0.066171 0.104258 -0.635 0.525761

Where_dog_is_keptApartment -0.135907 0.097188 -1.398 0.162276

dog_obedience2 tasks 0.302246 0.114082 2.649 0.008179 **

dog_obedience3 tasks 0.321454 0.110164 2.918 0.003595 **

dog_obedience>3 tasks 0.617234 0.104827 5.888 5.18e-09 ***

play30m-1h 0.238903 0.106737 2.238 0.025405 *

play1-3h 0.140138 0.108460 1.292 0.196604

play>3h 0.118006 0.132789 0.889 0.374373

commands11-30 0.088798 0.073600 1.206 0.227885

commands>30 0.307511 0.118197 2.602 0.009401 **

training2-3 -0.080240 0.080328 -0.999 0.318062

training4 more 0.078302 0.092814 0.844 0.399051

dog_beh_changed_3monthYes -0.018673 0.071421 -0.261 0.793801

---

Signif. codes: 0 ‘***’ 0.001 ‘**’ 0.01 ‘*’ 0.05 ‘.’ 0.1 ‘ ’ 1

Residual standard error: 0.9736 on 1104 degrees of freedom

(51 observations deleted due to missingness)

Multiple R-squared: 0.3329, Adjusted R-squared: 0.302

F-statistic: 10.8 on 51 and 1104 DF, p-value: < 2.2e-16

> Residuals <- residuals (lm)

> qqnorm(Residuals)

> shapiro.test (Residuals)

Shapiro-Wilk normality test

data: Residuals

W = 0.99718, p-value = 0.03878

### DPQFAC4 – Responsiveness to training

#### Age only

> lm<-lm(bcPower(DPQFAC4new,1.31)~(age), data = data, na.action = na.omit)

> etasq(lm, anova = TRUE, type=3)

Anova Table (Type III tests)

Response: bcPower(DPQFAC4new, 1.31)

Partial eta^2 Sum Sq Df F value Pr(>F)

(Intercept) 0.57052 2168.7 1 1563.555 < 2.2e-16 ***

age 0.04162 70.9 5 10.223 1.321e-09 ***

Residuals 1632.5 1177

---

Signif. codes: 0 ‘***’ 0.001 ‘**’ 0.01 ‘*’ 0.05 ‘.’ 0.1 ‘ ’ 1

> summary(lm)

Call:

lm(formula = bcPower(DPQFAC4new, 1.31) ~ (age), data = data,

na.action = na.omit)

Residuals:

Min 1Q Median 3Q Max

-3.5183 -0.8711 -0.0467 0.8838 2.6250

Coefficients:

Estimate Std. Error t value Pr(>|t|)

(Intercept) 3.47106 0.08778 39.542 < 2e-16 ***

ageGroup2 0.11379 0.11572 0.983 0.3256

ageGroup3 0.07170 0.12281 0.584 0.5594

ageGroup4 0.04723 0.12100 0.390 0.6964

ageGroup5 -0.21869 0.12673 -1.726 0.0847 .

ageGroup6 -0.57332 0.12030 -4.766 2.12e-06 ***

---

Signif. codes: 0 ‘***’ 0.001 ‘**’ 0.01 ‘*’ 0.05 ‘.’ 0.1 ‘ ’ 1

Residual standard error: 1.178 on 1177 degrees of freedom

(24 observations deleted due to missingness)

Multiple R-squared: 0.04162, Adjusted R-squared: 0.03755

F-statistic: 10.22 on 5 and 1177 DF, p-value: 1.321e-09

> Residuals <- residuals (lm)

> qqnorm(Residuals)

> shapiro.test (Residuals)

Shapiro-Wilk normality test

data: Residuals

W = 0.98913, p-value = 1.104e-07

> data$age <- as.factor(data$age)

> com=glht(lm,linfct=mcp(age="Tukey"))

> summary(com)

Simultaneous Tests for General Linear Hypotheses

Multiple Comparisons of Means: Tukey Contrasts

Fit: lm(formula = bcPower(DPQFAC4new, 1.31) ~ (age), data = data,

na.action = na.omit)

Linear Hypotheses:

Estimate Std. Error t value Pr(>|t|)

Group2 - Group1 == 0 0.11379 0.11572 0.983 0.923

Group3 - Group1 == 0 0.07170 0.12281 0.584 0.992

Group4 - Group1 == 0 0.04723 0.12100 0.390 0.999

Group5 - Group1 == 0 -0.21869 0.12673 -1.726 0.514

Group6 - Group1 == 0 -0.57332 0.12030 -4.766 <0.001 ***

Group3 - Group2 == 0 -0.04209 0.11429 -0.368 0.999

Group4 - Group2 == 0 -0.06656 0.11234 -0.593 0.992

Group5 - Group2 == 0 -0.33248 0.11849 -2.806 0.057 .

Group6 - Group2 == 0 -0.68711 0.11158 -6.158 <0.001 ***

Group4 - Group3 == 0 -0.02447 0.11964 -0.205 1.000

Group5 - Group3 == 0 -0.29039 0.12543 -2.315 0.188

Group6 - Group3 == 0 -0.64503 0.11893 -5.424 <0.001 ***

Group5 - Group4 == 0 -0.26592 0.12366 -2.150 0.262

Group6 - Group4 == 0 -0.62055 0.11705 -5.302 <0.001 ***

Group6 - Group5 == 0 -0.35464 0.12297 -2.884 0.046 *

---

Signif. codes: 0 ‘***’ 0.001 ‘**’ 0.01 ‘*’ 0.05 ‘.’ 0.1 ‘ ’ 1

(Adjusted p values reported -- single-step method)

#### Full model

> lm<-lm(bcPower(DPQFAC4new,1.31)~(age+sensory+height+weight+breed+sex+neuter+offleash_activity+BCS+food+Add_vitamins+trauma+Health_prob+medication+Owner_age+Owner_dog_exp+How_many_dogs_in_house+How_many_people+Child+Age_of_dog_when_arrived+get_dog+Where_dog_is_kept+dog_obedience+play+commands+training+Time_dog_spend_alone+dog_beh_changed_3month), data = data, na.action = na.omit)

> etasq(lm, anova = TRUE, type=3)

Anova Table (Type III tests)

Response: bcPower(DPQFAC4new, 1.31)

Partial eta^2 Sum Sq Df F value Pr(>F)

(Intercept) 0.042604 40.34 1 49.8402 2.920e-12 ***

age 0.004785 4.36 5 1.0770 0.371361

sensory 0.000791 0.72 1 0.8865 0.346622

height 0.001939 1.76 1 2.1760 0.140459

weight 0.006116 5.58 1 6.8926 0.008773 **

breed 0.000288 0.26 1 0.3226 0.570187

sex 0.007822 7.15 1 8.8303 0.003026 **

neuter 0.001126 1.02 1 1.2623 0.261455

offleash_activity 0.013556 12.46 4 3.8478 0.004118 **

BCS 0.000864 0.78 2 0.4845 0.616136

food 0.010953 10.04 4 3.1008 0.014956 *

Add_vitamins 0.003393 3.09 3 1.2709 0.282949

trauma 0.000915 0.83 1 1.0254 0.311470

Health_prob 0.005212 4.75 4 1.4670 0.210002

medication 0.000001 0.00 1 0.0009 0.976203

Owner_age 0.004400 4.01 3 1.6498 0.176184

Owner_dog_exp 0.005007 4.56 2 2.8182 0.060134 .

How_many_dogs_in_house 0.000145 0.13 2 0.0814 0.921816

How_many_people 0.003572 3.25 3 1.3383 0.260445

Child 0.001095 0.99 1 1.2272 0.268191

Age_of_dog_when_arrived 0.001758 1.60 3 0.6575 0.578302

get_dog 0.000028 0.03 2 0.0156 0.984474

Where_dog_is_kept 0.003041 2.76 2 1.7079 0.181719

dog_obedience 0.184699 205.38 3 84.5755 < 2.2e-16 ***

play 0.012640 11.61 3 4.7793 0.002580 **

commands 0.031105 29.10 2 17.9781 2.065e-08 ***

training 0.006030 5.50 2 3.3973 0.033810 *

Time_dog_spend_alone 0.006202 5.66 3 2.3299 0.072830 .

dog_beh_changed_3month 0.004408 4.01 1 4.9587 0.026158 *

Residuals 906.58 1120

---

Signif. codes: 0 ‘***’ 0.001 ‘**’ 0.01 ‘*’ 0.05 ‘.’ 0.1 ‘ ’ 1

> summary(lm)

Call:

lm(formula = bcPower(DPQFAC4new, 1.31) ~ (age + sensory + height +

weight + breed + sex + neuter + offleash_activity + BCS +

food + Add_vitamins + trauma + Health_prob + medication +

Owner_age + Owner_dog_exp + How_many_dogs_in_house + How_many_people +

Child + Age_of_dog_when_arrived + get_dog + Where_dog_is_kept +

dog_obedience + play + commands + training + Time_dog_spend_alone +

dog_beh_changed_3month), data = data, na.action = na.omit)

Residuals:

Min 1Q Median 3Q Max

-2.65335 -0.57800 0.00286 0.61839 3.04808

Coefficients:

Estimate Std. Error t value Pr(>|t|)

(Intercept) 1.908e+00 2.703e-01 7.060 2.92e-12 ***

ageGroup2 1.255e-01 9.343e-02 1.343 0.179417

ageGroup3 7.574e-02 9.990e-02 0.758 0.448503

ageGroup4 1.678e-01 1.033e-01 1.623 0.104801

ageGroup5 4.242e-02 1.104e-01 0.384 0.700715

ageGroup6 -1.957e-02 1.181e-01 -0.166 0.868484

sensoryPresent 8.129e-02 8.634e-02 0.942 0.346622

height -3.939e-03 2.670e-03 -1.475 0.140459

weight 8.697e-03 3.313e-03 2.625 0.008773 **

breedPure -4.085e-02 7.192e-02 -0.568 0.570187

sexMale -1.681e-01 5.656e-02 -2.972 0.003026 **

neuterNeutered -6.841e-02 6.089e-02 -1.124 0.261455

offleash_activity30mins/1hour 1.282e-01 9.379e-02 1.367 0.172028

offleash_activity1/3 3.118e-01 9.082e-02 3.434 0.000617 ***

offleash_activity3/7 3.161e-01 1.081e-01 2.924 0.003530 **

offleash_activity>7 2.161e-01 1.058e-01 2.042 0.041378 *

BCSNormal 3.073e-02 7.507e-02 0.409 0.682362

BCSOverweight 9.116e-02 9.639e-02 0.946 0.344486

foodTinned and/or Dry food 3.841e-02 9.826e-02 0.391 0.695932

foodCooked food 9.357e-02 7.995e-02 1.170 0.242089

foodMixed -1.269e-01 8.459e-02 -1.500 0.133844

foodRaw 1.616e-01 8.894e-02 1.817 0.069485 .

Add_vitaminsRarely 1.131e-02 7.117e-02 0.159 0.873766

Add_vitaminsOften -6.071e-02 8.146e-02 -0.745 0.456223

Add_vitaminsDaily 1.058e-01 8.505e-02 1.244 0.213698

traumaYes -6.134e-02 6.058e-02 -1.013 0.311470

Health_probTooth problem only -1.924e-01 9.028e-02 -2.131 0.033290 *

Health_probJoint & Tooth probs -1.968e-01 1.072e-01 -1.836 0.066606 .

Health_probJoint problems only -1.097e-01 8.201e-02 -1.338 0.181117

Health_probOther disorders -1.042e-01 8.874e-02 -1.174 0.240798

medicationYes -2.419e-03 8.107e-02 -0.030 0.976203

Owner_age30-39 -1.749e-02 7.627e-02 -0.229 0.818635

Owner_age40-49 4.678e-02 8.508e-02 0.550 0.582587

Owner_age>50 -1.444e-01 9.029e-02 -1.599 0.110057

Owner_dog_expHad a dog before -1.119e-01 7.026e-02 -1.593 0.111499

Owner_dog_expNever had a dog -2.008e-01 8.485e-02 -2.366 0.018145 *

How_many_dogs_in_houseOne -2.430e-02 6.234e-02 -0.390 0.696755

How_many_dogs_in_houseTwo more -2.067e-02 7.570e-02 -0.273 0.784891

How_many_peopleTwo people 1.318e-01 9.124e-02 1.444 0.149008

How_many_peopleThree people 1.923e-02 1.102e-01 0.175 0.861493

How_many_peopleFour more 1.074e-02 1.133e-01 0.095 0.924520

ChildYes 8.970e-02 8.097e-02 1.108 0.268191

Age_of_dog_when_arrived7-12w 1.879e-02 7.131e-02 0.264 0.792189

Age_of_dog_when_arrived3-12m -5.933e-02 9.079e-02 -0.653 0.513590

Age_of_dog_when_arrived>1y 7.012e-02 1.003e-01 0.699 0.484460

get_dogborn bought 5.567e-05 9.716e-02 0.001 0.999543

get_dogpresent -1.089e-02 8.467e-02 -0.129 0.897725

Where_dog_is_keptGarden 1.008e-01 9.499e-02 1.061 0.288988

Where_dog_is_keptApartment 1.627e-01 8.865e-02 1.835 0.066736 .

dog_obedience2 tasks 2.488e-01 1.049e-01 2.371 0.017896 *

dog_obedience3 tasks 6.752e-01 1.007e-01 6.708 3.13e-11 ***

dog_obedience>3 tasks 1.332e+00 9.643e-02 13.812 < 2e-16 ***

play30m-1h 1.912e-01 9.889e-02 1.934 0.053415 .

play1-3h 3.097e-01 1.007e-01 3.074 0.002160 **

play>3h 4.257e-01 1.218e-01 3.494 0.000495 ***

commands11-30 2.983e-01 6.780e-02 4.399 1.19e-05 ***

commands>30 6.269e-01 1.095e-01 5.726 1.32e-08 ***

training2-3 1.744e-01 7.404e-02 2.355 0.018671 *

training4 more 1.521e-01 8.563e-02 1.777 0.075900 .

Time_dog_spend_alone1-2h -2.139e-01 9.249e-02 -2.313 0.020897 *

Time_dog_spend_alone3-8h -5.986e-02 8.332e-02 -0.718 0.472609

Time_dog_spend_alone>8h -6.669e-02 1.063e-01 -0.627 0.530718

dog_beh_changed_3monthYes -1.453e-01 6.525e-02 -2.227 0.026158 *

---

Signif. codes: 0 ‘***’ 0.001 ‘**’ 0.01 ‘*’ 0.05 ‘.’ 0.1 ‘ ’ 1

Residual standard error: 0.8997 on 1120 degrees of freedom

(27 observations deleted due to missingness)

Multiple R-squared: 0.4678, Adjusted R-squared: 0.4383

F-statistic: 15.88 on 62 and 1120 DF, p-value: < 2.2e-16

> Residuals <- residuals (lm)

> qqnorm(Residuals)

> shapiro.test (Residuals)

Shapiro-Wilk normality test

data: Residuals

W = 0.99863, p-value = 0.4932

#### Reduced Model

> lm<-lm(bcPower(DPQFAC4new,1.31)~(age+sensory+height+weight+breed+sex+neuter+offleash_activity+BCS+food+Add_vitamins+trauma+Health_prob+medication+Owner_age+Age_of_dog_when_arrived+get_dog+Where_dog_is_kept+dog_obedience+play+commands+training+dog_beh_changed_3month), data = data, na.action = na.omit)

> etasq(lm, anova = TRUE, type=3)

Anova Table (Type III tests)

Response: bcPower(DPQFAC4new, 1.31)

Partial eta^2 Sum Sq Df F value Pr(>F)

(Intercept) 0.048879 47.34 1 58.1229 5.197e-14 ***

age 0.004736 4.38 5 1.0765 0.371646

sensory 0.001339 1.23 1 1.5162 0.218448

height 0.001848 1.71 1 2.0943 0.148125

weight 0.006185 5.73 1 7.0392 0.008086 **

breed 0.000210 0.19 1 0.2377 0.625940

sex 0.009054 8.42 1 10.3338 0.001343 **

neuter 0.000877 0.81 1 0.9925 0.319340

offleash_activity 0.013510 12.62 4 3.8723 0.003944 **

BCS 0.000680 0.63 2 0.3850 0.680555

food 0.010357 9.64 4 2.9591 0.019023 *

Add_vitamins 0.003773 3.49 3 1.4277 0.233082

trauma 0.000711 0.66 1 0.8051 0.369750

Health_prob 0.005749 5.33 4 1.6349 0.163137

medication 0.000142 0.13 1 0.1611 0.688265

Owner_age 0.003962 3.66 3 1.4995 0.213041

Age_of_dog_when_arrived 0.001902 1.76 3 0.7185 0.541000

get_dog 0.000051 0.05 2 0.0286 0.971833

Where_dog_is_kept 0.002459 2.27 2 1.3940 0.248497

dog_obedience 0.181872 204.78 3 83.8079 < 2.2e-16 ***

play 0.011035 10.28 3 4.2065 0.005704 **

commands 0.034095 32.52 2 19.9613 3.023e-09 ***

training 0.007136 6.62 2 4.0644 0.017424 *

dog_beh_changed_3month 0.004424 4.09 1 5.0253 0.025173 *

Residuals 921.19 1131

---

Signif. codes: 0 ‘***’ 0.001 ‘**’ 0.01 ‘*’ 0.05 ‘.’ 0.1 ‘ ’ 1

> summary(lm)

Call:

lm(formula = bcPower(DPQFAC4new, 1.31) ~ (age + sensory + height +

weight + breed + sex + neuter + offleash_activity + BCS +

food + Add_vitamins + trauma + Health_prob + medication +

Owner_age + Age_of_dog_when_arrived + get_dog + Where_dog_is_kept +

dog_obedience + play + commands + training + dog_beh_changed_3month),

data = data, na.action = na.omit)

Residuals:

Min 1Q Median 3Q Max

-2.71396 -0.59663 0.02132 0.63222 2.99536

Coefficients:

Estimate Std. Error t value Pr(>|t|)

(Intercept) 1.778480 0.233279 7.624 5.20e-14 ***

ageGroup2 0.128956 0.093182 1.384 0.166658

ageGroup3 0.063353 0.099979 0.634 0.526433

ageGroup4 0.156507 0.102800 1.522 0.128176

ageGroup5 0.033894 0.109976 0.308 0.757991

ageGroup6 -0.027964 0.117731 -0.238 0.812290

sensoryPresent 0.106183 0.086233 1.231 0.218448

height -0.003849 0.002660 -1.447 0.148125

weight 0.008758 0.003301 2.653 0.008086 **

breedPure -0.035025 0.071834 -0.488 0.625940

sexMale -0.181032 0.056315 -3.215 0.001343 **

neuterNeutered -0.060569 0.060797 -0.996 0.319340

offleash_activity30mins/1hour 0.134600 0.093490 1.440 0.150224

offleash_activity1/3 0.315998 0.090758 3.482 0.000517 ***

offleash_activity3/7 0.314703 0.107189 2.936 0.003392 **

offleash_activity>7 0.220245 0.105528 2.087 0.037105 *

BCSNormal 0.018015 0.074396 0.242 0.808703

BCSOverweight 0.076517 0.095691 0.800 0.424095

foodTinned and/or Dry food 0.020718 0.097590 0.212 0.831911

foodCooked food 0.078226 0.079163 0.988 0.323281

foodMixed -0.115570 0.083332 -1.387 0.165758

foodRaw 0.174438 0.088703 1.967 0.049481 *

Add_vitaminsRarely 0.027505 0.070819 0.388 0.697808

Add_vitaminsOften -0.043975 0.080777 -0.544 0.586271

Add_vitaminsDaily 0.127648 0.084307 1.514 0.130286

traumaYes -0.054225 0.060431 -0.897 0.369750

Health_probTooth problem only -0.202190 0.089949 -2.248 0.024779 *

Health_probJoint & Tooth probs -0.203276 0.106903 -1.902 0.057490 .

Health_probJoint problems only -0.125535 0.081755 -1.535 0.124942

Health_probOther disorders -0.119774 0.088311 -1.356 0.175284

medicationYes -0.032388 0.080704 -0.401 0.688265

Owner_age30-39 0.034133 0.070548 0.484 0.628607

Owner_age40-49 0.066558 0.076195 0.874 0.382559

Owner_age>50 -0.106449 0.081733 -1.302 0.193045

Age_of_dog_when_arrived7-12w 0.007316 0.070830 0.103 0.917748

Age_of_dog_when_arrived3-12m -0.064252 0.090694 -0.708 0.478813

Age_of_dog_when_arrived>1y 0.075584 0.100331 0.753 0.451398

get_dogborn bought 0.008089 0.096717 0.084 0.933363

get_dogpresent -0.008932 0.084295 -0.106 0.915633

Where_dog_is_keptGarden 0.095543 0.094660 1.009 0.313033

Where_dog_is_keptApartment 0.146348 0.087947 1.664 0.096381 .

dog_obedience2 tasks 0.250568 0.104591 2.396 0.016750 *

dog_obedience3 tasks 0.665136 0.100381 6.626 5.32e-11 ***

dog_obedience>3 tasks 1.326076 0.096233 13.780 < 2e-16 ***

play30m-1h 0.181654 0.098722 1.840 0.066021 .

play1-3h 0.286563 0.100398 2.854 0.004393 **

play>3h 0.402052 0.121260 3.316 0.000943 ***

commands11-30 0.308254 0.067541 4.564 5.57e-06 ***

commands>30 0.656564 0.108383 6.058 1.88e-09 ***

training2-3 0.187175 0.073875 2.534 0.011422 *

training4 more 0.172144 0.085302 2.018 0.043821 *

dog_beh_changed_3monthYes -0.146095 0.065171 -2.242 0.025173 *

---

Signif. codes: 0 ‘***’ 0.001 ‘**’ 0.01 ‘*’ 0.05 ‘.’ 0.1 ‘ ’ 1

Residual standard error: 0.9025 on 1131 degrees of freedom

(27 observations deleted due to missingness)

Multiple R-squared: 0.4592, Adjusted R-squared: 0.4348

F-statistic: 18.83 on 51 and 1131 DF, p-value: < 2.2e-16

> Residuals <- residuals (lm)

> qqnorm(Residuals)

> shapiro.test (Residuals)

Shapiro-Wilk normality test

data: Residuals

W = 0.99917, p-value = 0.8927

#### Outliers removed (1 removed)

#### Mediation Analysis

> A <- polr(play ~ age+sensory+height+weight+breed+sex+neuter+offleash_activity+BCS+food+Add_vitamins+trauma+Health_prob+medication+Owner_age+Age_of_dog_when_arrived+get_dog+Where_dog_is_kept+commands+dog_obedience+training+dog_beh_changed_3month , data=new_cog_no_missing_new, na.action = na.omit,method = "probit", Hess = TRUE)

> B <- lm(bcPower(DPQFAC4new,1.31)~(play+age+sensory+height+weight+breed+sex+neuter+offleash_activity+BCS+food+Add_vitamins+trauma+Health_prob+medication+Owner_age+Age_of_dog_when_arrived+get_dog+Where_dog_is_kept+dog_obedience+commands+training+dog_beh_changed_3month), data=new_cog_no_missing_new, na.action = na.omit)

> Mediation <- mediate(A,B, treat="age", mediator="play", ,control.value = "Group2", treat.value = "Group6", robustSE = TRUE, sims = 100, data=data)

> summary(Mediation)

Causal Mediation Analysis

Quasi-Bayesian Confidence Intervals

Estimate 95% CI Lower 95% CI Upper p-value

ACME -0.0365 -0.0678 -0.01 <2e-16 ***

ADE -0.1365 -0.3147 0.10 0.16

Total Effect -0.1729 -0.3639 0.07 0.12

Prop. Mediated 0.1766 -0.5282 0.84 0.12

---

Signif. codes: 0 ‘***’ 0.001 ‘**’ 0.01 ‘*’ 0.05 ‘.’ 0.1 ‘ ’ 1

Sample Size Used: 1183

Simulations: 100

> plot(Mediation)


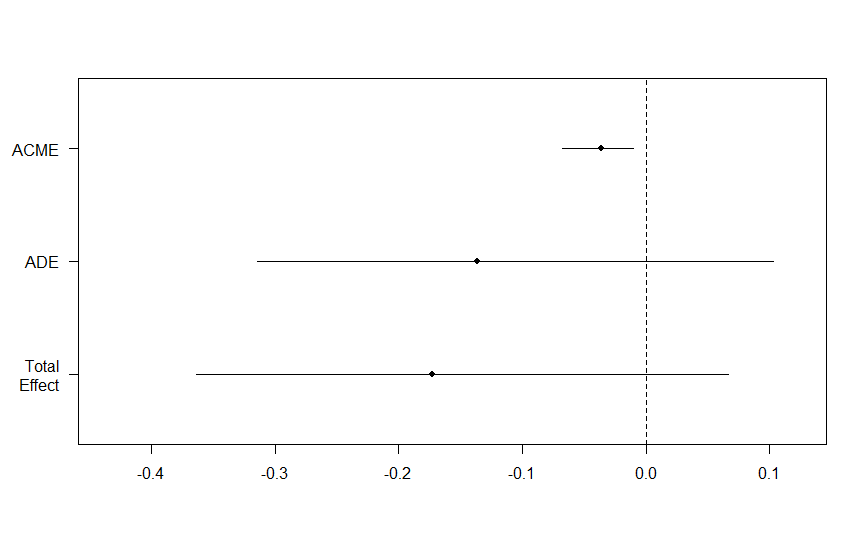


**Figure 2. Estimates (points) and 95% confidence intervals for the average causal mediation effect (ACME), average direct effect (ADE), and total effect.**

When comparing age group two and group six (adult vs old dogs), result indicate that there was a significant average causal mediation effect (ACME), but the estimated average direct effect and the total effect was not significant. The results suggest that the change in the play variable (mediator) in older dogs in part drove the lower Responsiveness to training personality trait score, as it explained the relationship better than the age of the dog (direct effect) in itself. The average proportion mediated was 18%.

### DPQFAC5 – Aggressiveness to animals

#### Age only

> lm<-lm(bcPower(DPQFAC5new,0.46)~(age), data = data, na.action = na.omit)

> etasq(lm, anova = TRUE, type=3)

Anova Table (Type III tests)

Response: bcPower(DPQFAC5new, 0.46)

Partial eta^2 Sum Sq Df F value Pr(>F)

(Intercept) 0.50765 242.195 1 1201.1953 < 2.2e-16 ***

age 0.01948 4.666 5 4.6284 0.0003459 ***

Residuals 234.897 1165

---

Signif. codes: 0 ‘***’ 0.001 ‘**’ 0.01 ‘*’ 0.05 ‘.’ 0.1 ‘ ’ 1

> summary(lm)

Call:

lm(formula = bcPower(DPQFAC5new, 0.46) ~ (age), data = data,

na.action = na.omit)

Residuals:

Min 1Q Median 3Q Max

-1.30880 -0.31826 -0.00171 0.32050 1.18772

Coefficients:

Estimate Std. Error t value Pr(>|t|)

(Intercept) 1.16976 0.03375 34.658 < 2e-16 ***

ageGroup2 0.01667 0.04433 0.376 0.70696

ageGroup3 0.13627 0.04728 2.883 0.00402 **

ageGroup4 0.13904 0.04645 2.993 0.00282 **

ageGroup5 0.04012 0.04852 0.827 0.40841

ageGroup6 -0.02028 0.04623 -0.439 0.66104

---

Signif. codes: 0 ‘***’ 0.001 ‘**’ 0.01 ‘*’ 0.05 ‘.’ 0.1 ‘ ’ 1

Residual standard error: 0.449 on 1165 degrees of freedom

(36 observations deleted due to missingness)

Multiple R-squared: 0.01948, Adjusted R-squared: 0.01527

F-statistic: 4.628 on 5 and 1165 DF, p-value: 0.0003459

> Residuals <- residuals (lm)

> qqnorm(Residuals)

> shapiro.test (Residuals)

Shapiro-Wilk normality test

data: Residuals

W = 0.9968, p-value = 0.01739

> com=glht(lm,linfct=mcp(age="Tukey"))

> summary(com)

Simultaneous Tests for General Linear Hypotheses

Multiple Comparisons of Means: Tukey Contrasts

Fit: lm(formula = bcPower(DPQFAC5new, 0.46) ~ (age), data = data,

na.action = na.omit)

Linear Hypotheses:

Estimate Std. Error t value Pr(>|t|)

Group2 - Group1 == 0 0.016671 0.044334 0.376 0.99902

Group3 - Group1 == 0 0.136271 0.047275 2.883 0.04599 *

Group4 - Group1 == 0 0.139037 0.046449 2.993 0.03338 *

Group5 - Group1 == 0 0.040122 0.048516 0.827 0.96244

Group6 - Group1 == 0 -0.020277 0.046231 -0.439 0.99794

Group3 - Group2 == 0 0.119600 0.043842 2.728 0.07037 .

Group4 - Group2 == 0 0.122366 0.042950 2.849 0.05056 .

Group5 - Group2 == 0 0.023451 0.045177 0.519 0.99542

Group6 - Group2 == 0 -0.036948 0.042714 -0.865 0.95455

Group4 - Group3 == 0 0.002766 0.045980 0.060 1.00000

Group5 - Group3 == 0 -0.096149 0.048067 -2.000 0.34201

Group6 - Group3 == 0 -0.156548 0.045760 -3.421 0.00838 **

Group5 - Group4 == 0 -0.098915 0.047254 -2.093 0.29083

Group6 - Group4 == 0 -0.159314 0.044905 -3.548 0.00541 **

Group6 - Group5 == 0 -0.060399 0.047040 -1.284 0.79340

---

Signif. codes: 0 ‘***’ 0.001 ‘**’ 0.01 ‘*’ 0.05 ‘.’ 0.1 ‘ ’ 1

(Adjusted p values reported -- single-step method)

#### Full Model

> lm<-lm(bcPower(DPQFAC5new,0.46)~(age+sensory+height+weight+breed+sex+neuter+offleash_activity+BCS+food+Add_vitamins+trauma+Health_prob+medication+Owner_age+Owner_dog_exp+How_many_dogs_in_house+How_many_people+Child+Age_of_dog_when_arrived+get_dog+Where_dog_is_kept+dog_obedience+play+commands+training+Time_dog_spend_alone+dog_beh_changed_3month), data = data, na.action = na.omit)

> etasq(lm, anova = TRUE, type=3)

Anova Table (Type III tests)

Response: bcPower(DPQFAC5new, 0.46)

Partial eta^2 Sum Sq Df F value Pr(>F)

(Intercept) 0.090987 21.048 1 110.9038 < 2.2e-16 ***

age 0.018420 3.946 5 4.1584 0.0009525 ***

sensory 0.005018 1.060 1 5.5875 0.0182604 *

height 0.001418 0.299 1 1.5735 0.2099614

weight 0.001579 0.333 1 1.7525 0.1858356

breed 0.005218 1.103 1 5.8118 0.0160813 *

sex 0.002190 0.462 1 2.4323 0.1191418

neuter 0.002981 0.629 1 3.3129 0.0690098 .

offleash_activity 0.006275 1.328 4 1.7492 0.1368848

BCS 0.003027 0.638 2 1.6822 0.1864470

food 0.012712 2.707 4 3.5665 0.0067197 **

Add_vitamins 0.001808 0.381 3 0.6690 0.5711499

trauma 0.007208 1.527 1 8.0447 0.0046468 **

Health_prob 0.007521 1.594 4 2.0991 0.0788526 .

medication 0.000037 0.008 1 0.0414 0.8388949

Owner_age 0.000442 0.093 3 0.1634 0.9210305

Owner_dog_exp 0.003252 0.686 2 1.8076 0.1645372

How_many_dogs_in_house 0.000262 0.055 2 0.1453 0.8647575

How_many_people 0.002964 0.625 3 1.0979 0.3489864

Child 0.000093 0.019 1 0.1026 0.7487404

Age_of_dog_when_arrived 0.009424 2.000 3 3.5135 0.0147911 *

get_dog 0.000108 0.023 2 0.0598 0.9420009

Where_dog_is_kept 0.004675 0.988 2 2.6021 0.0745714 .

dog_obedience 0.019678 4.221 3 7.4135 6.429e-05 ***

play 0.000715 0.150 3 0.2643 0.8511409

commands 0.001349 0.284 2 0.7482 0.4734542

training 0.000701 0.147 2 0.3884 0.6782509

Time_dog_spend_alone 0.000315 0.066 3 0.1162 0.9506192

dog_beh_changed_3month 0.002784 0.587 1 3.0932 0.0788946 .

Residuals 210.282 1108

---

Signif. codes: 0 ‘***’ 0.001 ‘**’ 0.01 ‘*’ 0.05 ‘.’ 0.1 ‘ ’ 1

> summary(lm)

Call:

lm(formula = bcPower(DPQFAC5new, 0.46) ~ (age + sensory + height +

weight + breed + sex + neuter + offleash_activity + BCS +

food + Add_vitamins + trauma + Health_prob + medication +

Owner_age + Owner_dog_exp + How_many_dogs_in_house + How_many_people +

Child + Age_of_dog_when_arrived + get_dog + Where_dog_is_kept +

dog_obedience + play + commands + training + Time_dog_spend_alone +

dog_beh_changed_3month), data = data, na.action = na.omit)

Residuals:

Min 1Q Median 3Q Max

-1.20798 -0.29223 0.00088 0.30067 1.16770

Coefficients:

Estimate Std. Error t value Pr(>|t|)

(Intercept) 1.3837036 0.1313923 10.531 < 2e-16 ***

ageGroup2 0.0233984 0.0453945 0.515 0.60634

ageGroup3 0.1297475 0.0488956 2.654 0.00808 **

ageGroup4 0.1190091 0.0503388 2.364 0.01824 *

ageGroup5 0.0261860 0.0537732 0.487 0.62638

ageGroup6 -0.0573886 0.0578182 -0.993 0.32114

sensoryPresent -0.0990558 0.0419053 -2.364 0.01826 *

height -0.0016337 0.0013024 -1.254 0.20996

weight 0.0021307 0.0016095 1.324 0.18584

breedPure -0.0847358 0.0351490 -2.411 0.01608 *

sexMale 0.0429217 0.0275211 1.560 0.11914

neuterNeutered -0.0540700 0.0297068 -1.820 0.06901 .

offleash_activity30mins/1hour -0.0379077 0.0457899 -0.828 0.40793

offleash_activity1/3 -0.1079415 0.0444519 -2.428 0.01533 *

offleash_activity3/7 -0.0576964 0.0526828 -1.095 0.27368

offleash_activity>7 -0.0638412 0.0514923 -1.240 0.21530

BCSNormal -0.0272441 0.0366874 -0.743 0.45788

BCSOverweight -0.0823372 0.0469561 -1.753 0.07979 .

foodTinned and/or Dry food -0.1051971 0.0476484 -2.208 0.02746 *

foodCooked food 0.0281214 0.0390522 0.720 0.47162

foodMixed 0.0651751 0.0412314 1.581 0.11423

foodRaw 0.0391445 0.0431708 0.907 0.36474

Add_vitaminsRarely -0.0433078 0.0347075 -1.248 0.21237

Add_vitaminsOften -0.0215622 0.0396395 -0.544 0.58658

Add_vitaminsDaily -0.0007903 0.0413517 -0.019 0.98476

traumaYes 0.0835928 0.0294723 2.836 0.00465 **

Health_probTooth problem only 0.1221577 0.0441015 2.770 0.00570 **

Health_probJoint & Tooth probs 0.0567985 0.0522646 1.087 0.27738

Health_probJoint problems only 0.0631386 0.0399910 1.579 0.11466

Health_probOther disorders 0.0724829 0.0431997 1.678 0.09366 .

medicationYes -0.0080155 0.0394162 -0.203 0.83889

Owner_age30-39 0.0126925 0.0371572 0.342 0.73272

Owner_age40-49 0.0145717 0.0414156 0.352 0.72503

Owner_age>50 0.0305313 0.0436255 0.700 0.48417

Owner_dog_expHad a dog before -0.0253641 0.0345066 -0.735 0.46246

Owner_dog_expNever had a dog 0.0384646 0.0414810 0.927 0.35398

How_many_dogs_in_houseOne 0.0151077 0.0302980 0.499 0.61814

How_many_dogs_in_houseTwo more 0.0017166 0.0368805 0.047 0.96289

How_many_peopleTwo people 0.0008694 0.0439407 0.020 0.98422

How_many_peopleThree people -0.0377085 0.0535342 -0.704 0.48134

How_many_peopleFour more 0.0318051 0.0546221 0.582 0.56050

ChildYes 0.0126040 0.0393406 0.320 0.74874

Age_of_dog_when_arrived7-12w 0.0708828 0.0347013 2.043 0.04132 *

Age_of_dog_when_arrived3-12m 0.0369878 0.0442849 0.835 0.40377

Age_of_dog_when_arrived>1y -0.0652826 0.0485622 -1.344 0.17912

get_dogborn bought 0.0003251 0.0472103 0.007 0.99451

get_dogpresent 0.0105984 0.0410075 0.258 0.79611

Where_dog_is_keptGarden -0.0277291 0.0464701 -0.597 0.55082

Where_dog_is_keptApartment -0.0881388 0.0433433 -2.034 0.04224 *

dog_obedience2 tasks 0.0566546 0.0512542 1.105 0.26924

dog_obedience3 tasks -0.0582131 0.0490294 -1.187 0.23536

dog_obedience>3 tasks -0.1391010 0.0468529 -2.969 0.00305 **

play30m-1h 0.0029871 0.0480561 0.062 0.95045

play1-3h -0.0151375 0.0489364 -0.309 0.75713

play>3h 0.0183506 0.0592464 0.310 0.75682

commands11-30 0.0380506 0.0329372 1.155 0.24824

commands>30 0.0494048 0.0534102 0.925 0.35516

training2-3 -0.0131831 0.0360419 -0.366 0.71461

training4 more 0.0269726 0.0416556 0.648 0.51743

Time_dog_spend_alone1-2h -0.0112622 0.0449551 -0.251 0.80223

Time_dog_spend_alone3-8h -0.0177099 0.0404807 -0.437 0.66184

Time_dog_spend_alone>8h -0.0290259 0.0518037 -0.560 0.57538

dog_beh_changed_3monthYes 0.0559552 0.0318152 1.759 0.07889 .

---

Signif. codes: 0 ‘***’ 0.001 ‘**’ 0.01 ‘*’ 0.05 ‘.’ 0.1 ‘ ’ 1

Residual standard error: 0.4356 on 1108 degrees of freedom

(36 observations deleted due to missingness)

Multiple R-squared: 0.1222, Adjusted R-squared: 0.07311

F-statistic: 2.488 on 62 and 1108 DF, p-value: 4.968e-09

> Residuals <- residuals (lm)

> qqnorm(Residuals)

> shapiro.test (Residuals)

Shapiro-Wilk normality test

data: Residuals

W = 0.99801, p-value = 0.1773

#### Reduced Model

> lm<-lm(bcPower(DPQFAC5new,0.46)~(age+sensory+height+weight+breed+sex+neuter+offleash_activity+BCS+food+Add_vitamins+trauma+Health_prob+medication+Owner_age+Age_of_dog_when_arrived+get_dog+Where_dog_is_kept+dog_obedience+play+commands+training+dog_beh_changed_3month), data = data, na.action = na.omit)

> etasq(lm, anova = TRUE, type=3)

Anova Table (Type III tests)

Response: bcPower(DPQFAC5new, 0.46)

Partial eta^2 Sum Sq Df F value Pr(>F)

(Intercept) 0.115071 27.533 1 145.5086 < 2.2e-16 ***

age 0.018045 3.891 5 4.1128 0.001049 **

sensory 0.005304 1.129 1 5.9671 0.014729 *

height 0.001130 0.240 1 1.2661 0.260745

weight 0.001349 0.286 1 1.5121 0.219078

breed 0.005192 1.105 1 5.8406 0.015819 *

sex 0.002442 0.518 1 2.7392 0.098195 .

neuter 0.002731 0.580 1 3.0646 0.080287 .

offleash_activity 0.006625 1.412 4 1.8656 0.114198

BCS 0.003297 0.700 2 1.8511 0.157553

food 0.012159 2.606 4 3.4434 0.008310 **

Add_vitamins 0.001976 0.419 3 0.7385 0.529144

trauma 0.007541 1.609 1 8.5021 0.003618 **

Health_prob 0.007277 1.552 4 2.0506 0.085210 .

medication 0.000056 0.012 1 0.0626 0.802451

Owner_age 0.000284 0.060 3 0.1061 0.956535

Age_of_dog_when_arrived 0.008570 1.830 3 3.2241 0.021937 *

get_dog 0.000124 0.026 2 0.0692 0.933166

Where_dog_is_kept 0.004534 0.964 2 2.5484 0.078660 .

dog_obedience 0.019132 4.130 3 7.2753 7.804e-05 ***

play 0.000665 0.141 3 0.2483 0.862590

commands 0.001429 0.303 2 0.8009 0.449188

training 0.000728 0.154 2 0.4076 0.665351

dog_beh_changed_3month 0.002647 0.562 1 2.9701 0.085091 .

Residuals 211.733 1119

---

Signif. codes: 0 ‘***’ 0.001 ‘**’ 0.01 ‘*’ 0.05 ‘.’ 0.1 ‘ ’ 1

> summary(lm)

Call:

lm(formula = bcPower(DPQFAC5new, 0.46) ~ (age + sensory + height +

weight + breed + sex + neuter + offleash_activity + BCS +

food + Add_vitamins + trauma + Health_prob + medication +

Owner_age + Age_of_dog_when_arrived + get_dog + Where_dog_is_kept +

dog_obedience + play + commands + training + dog_beh_changed_3month),

data = data, na.action = na.omit)

Residuals:

Min 1Q Median 3Q Max

-1.24056 -0.29913 0.00567 0.30524 1.20418

Coefficients:

Estimate Std. Error t value Pr(>|t|)

(Intercept) 1.3649496 0.1131546 12.063 < 2e-16 ***

ageGroup2 0.0202406 0.0450659 0.449 0.65342

ageGroup3 0.1258523 0.0486749 2.586 0.00985 **

ageGroup4 0.1193266 0.0498895 2.392 0.01693 *

ageGroup5 0.0257987 0.0532958 0.484 0.62843

ageGroup6 -0.0565072 0.0573645 -0.985 0.32481

sensoryPresent -0.1018116 0.0416789 -2.443 0.01473 *

height -0.0014544 0.0012926 -1.125 0.26074

weight 0.0019658 0.0015987 1.230 0.21908

breedPure -0.0844225 0.0349323 -2.417 0.01582 *

sexMale 0.0451027 0.0272516 1.655 0.09820 .

neuterNeutered -0.0516750 0.0295183 -1.751 0.08029 .

offleash_activity30mins/1hour -0.0333523 0.0454669 -0.734 0.46338

offleash_activity1/3 -0.1075649 0.0441986 -2.434 0.01510 *

offleash_activity3/7 -0.0474561 0.0520684 -0.911 0.36227

offleash_activity>7 -0.0643359 0.0511399 -1.258 0.20864

BCSNormal -0.0331552 0.0361798 -0.916 0.35966

BCSOverweight -0.0872588 0.0463783 -1.881 0.06017 .

foodTinned and/or Dry food -0.1046577 0.0471236 -2.221 0.02656 *

foodCooked food 0.0254849 0.0384979 0.662 0.50812

foodMixed 0.0615511 0.0404096 1.523 0.12800

foodRaw 0.0381196 0.0428538 0.890 0.37391

Add_vitaminsRarely -0.0446769 0.0343339 -1.301 0.19344

Add_vitaminsOften -0.0245025 0.0390993 -0.627 0.53100

Add_vitaminsDaily -0.0001864 0.0408067 -0.005 0.99636

traumaYes 0.0852561 0.0292389 2.916 0.00362 **

Health_probTooth problem only 0.1181565 0.0437415 2.701 0.00701 **

Health_probJoint & Tooth probs 0.0587334 0.0518558 1.133 0.25761

Health_probJoint problems only 0.0648310 0.0397103 1.633 0.10283

Health_probOther disorders 0.0747003 0.0427926 1.746 0.08115 .

medicationYes -0.0097669 0.0390307 -0.250 0.80245

Owner_age30-39 0.0063459 0.0342349 0.185 0.85298

Owner_age40-49 0.0119820 0.0371720 0.322 0.74726

Owner_age>50 0.0214319 0.0393183 0.545 0.58580

Age_of_dog_when_arrived7-12w 0.0610645 0.0343211 1.779 0.07548 .

Age_of_dog_when_arrived3-12m 0.0328991 0.0440508 0.747 0.45531

Age_of_dog_when_arrived>1y -0.0705237 0.0483640 -1.458 0.14507

get_dogborn bought 0.0109483 0.0467436 0.234 0.81486

get_dogpresent 0.0150605 0.0405747 0.371 0.71057

Where_dog_is_keptGarden -0.0236345 0.0460941 -0.513 0.60823

Where_dog_is_keptApartment -0.0843747 0.0427818 -1.972 0.04883 *

dog_obedience2 tasks 0.0592757 0.0508704 1.165 0.24417

dog_obedience3 tasks -0.0533578 0.0486774 -1.096 0.27325

dog_obedience>3 tasks -0.1345657 0.0465515 -2.891 0.00392 **

play30m-1h 0.0075999 0.0477593 0.159 0.87360

play1-3h -0.0109623 0.0485097 -0.226 0.82126

play>3h 0.0205161 0.0586810 0.350 0.72669

commands11-30 0.0394039 0.0326373 1.207 0.22756

commands>30 0.0488699 0.0526226 0.929 0.35325

training2-3 -0.0159693 0.0357867 -0.446 0.65551

training4 more 0.0249990 0.0412919 0.605 0.54502

dog_beh_changed_3monthYes 0.0545225 0.0316365 1.723 0.08509 .

---

Signif. codes: 0 ‘***’ 0.001 ‘**’ 0.01 ‘*’ 0.05 ‘.’ 0.1 ‘ ’ 1

Residual standard error: 0.435 on 1119 degrees of freedom

(36 observations deleted due to missingness)

Multiple R-squared: 0.1162, Adjusted R-squared: 0.07589

F-statistic: 2.884 on 51 and 1119 DF, p-value: 2.138e-10

> Residuals <- residuals (lm)

> qqnorm(Residuals)

> shapiro.test (Residuals)

Shapiro-Wilk normality test

data: Residuals

W = 0.99784, p-value = 0.1285

> data$food <- as.factor(data$food)

> com=glht(lm,linfct=mcp(food="Tukey"))

> summary(com)

Simultaneous Tests for General Linear Hypotheses

Multiple Comparisons of Means: Tukey Contrasts

Fit: lm(formula = bcPower(DPQFAC5new, 0.46) ~ (age + sensory + height +

weight + breed + sex + neuter + offleash_activity + BCS +

food + Add_vitamins + trauma + Health_prob + medication +

Owner_age + Age_of_dog_when_arrived + get_dog + Where_dog_is_kept +

dog_obedience + play + commands + training + dog_beh_changed_3month),

data = data, na.action = na.omit)

Linear Hypotheses:

Estimate Std. Error t value Pr(>|t|)

Tinned &/or Dry food-Dry food only== 0 -0.10466 0.04712 -2.221 0.17093

Cooked food - Dry food only == 0 0.02548 0.03850 0.662 0.96399

Mixed - Dry food only == 0 0.06155 0.04041 1.523 0.54492

Raw - Dry food only == 0 0.03812 0.04285 0.890 0.89986

Cooked food - Tinned &/or Dry food== 0 0.13014 0.04540 2.867 0.03388 *

Mixed - Tinned and/or Dry food == 0 0.16621 0.04696 3.540 0.00377 **

Raw - Tinned and/or Dry food == 0 0.14278 0.05023 2.843 0.03625 *

Mixed - Cooked food == 0 0.03607 0.03867 0.933 0.88312

Raw - Cooked food == 0 0.01263 0.04179 0.302 0.99816

Raw - Mixed == 0 -0.02343 0.04307 -0.544 0.98246

---

Signif. codes: 0 ‘***’ 0.001 ‘**’ 0.01 ‘*’ 0.05 ‘.’ 0.1 ‘ ’ 1

(Adjusted p values reported -- single-step method)

#### Outliers removed (1 removed)

> lm<-lm(bcPower(outDPQFAC5new,0.46)~(age+sensory+height+weight+breed+sex+neuter+offleash_activity+BCS+food+Add_vitamins+trauma+Health_prob+medication+Owner_age+Age_of_dog_when_arrived+get_dog+Where_dog_is_kept+dog_obedience+play+commands+training+dog_beh_changed_3month), data = data, na.action = na.omit)

> etasq(lm, anova = TRUE, type=3)

Anova Table (Type III tests)

Response: bcPower(outDPQFAC5new, 0.46)

Partial eta^2 Sum Sq Df F value Pr(>F)

(Intercept) 0.115140 27.351 1 145.4767 < 2.2e-16 ***

age 0.017785 3.806 5 4.0488 0.001202 **

sensory 0.006050 1.279 1 6.8055 0.009209 **

height 0.000982 0.207 1 1.0985 0.294832

weight 0.001180 0.248 1 1.3206 0.250734

breed 0.005195 1.098 1 5.8380 0.015843 *

sex 0.002856 0.602 1 3.2020 0.073820 .

neuter 0.002350 0.495 1 2.6330 0.104948

offleash_activity 0.007285 1.542 4 2.0510 0.085159 .

BCS 0.003134 0.661 2 1.7574 0.172961

food 0.012071 2.568 4 3.4150 0.008728 **

Add_vitamins 0.002075 0.437 3 0.7751 0.507969

trauma 0.006858 1.451 1 7.7198 0.005553 **

Health_prob 0.007841 1.661 4 2.2089 0.066063 .

medication 0.000183 0.038 1 0.2047 0.651058

Owner_age 0.000310 0.065 3 0.1155 0.951016

Age_of_dog_when_arrived 0.008250 1.749 3 3.1002 0.025954 *

get_dog 0.000127 0.027 2 0.0713 0.931218

Where_dog_is_kept 0.004217 0.890 2 2.3675 0.094182 .

dog_obedience 0.020620 4.425 3 7.8463 3.489e-05 ***

play 0.000683 0.144 3 0.2548 0.857973

commands 0.001760 0.371 2 0.9858 0.373456

training 0.000744 0.156 2 0.4161 0.659741

dog_beh_changed_3month 0.002271 0.478 1 2.5443 0.110975

Residuals 210.194 1118

---

Signif. codes: 0 ‘***’ 0.001 ‘**’ 0.01 ‘*’ 0.05 ‘.’ 0.1 ‘ ’ 1

> summary(lm)

Call:

lm(formula = bcPower(outDPQFAC5new, 0.46) ~ (age + sensory +

height + weight + breed + sex + neuter + offleash_activity +

BCS + food + Add_vitamins + trauma + Health_prob + medication +

Owner_age + Age_of_dog_when_arrived + get_dog + Where_dog_is_kept +

dog_obedience + play + commands + training + dog_beh_changed_3month),

data = data, na.action = na.omit)

Residuals:

Min 1Q Median 3Q Max

-1.2470 -0.2964 0.0037 0.3031 1.1243

Coefficients:

Estimate Std. Error t value Pr(>|t|)

(Intercept) 1.360562 0.112803 12.061 < 2e-16 ***

ageGroup2 0.019623 0.044922 0.437 0.66233

ageGroup3 0.127106 0.048521 2.620 0.00892 **

ageGroup4 0.120837 0.049733 2.430 0.01527 *

ageGroup5 0.020514 0.053157 0.386 0.69963

ageGroup6 -0.048145 0.057256 -0.841 0.40060

sensoryPresent -0.108555 0.041612 -2.609 0.00921 **

height -0.001351 0.001289 -1.048 0.29483

weight 0.001832 0.001594 1.149 0.25073

breedPure -0.084133 0.034821 -2.416 0.01584 *

sexMale 0.048659 0.027193 1.789 0.07382 .

neuterNeutered -0.047795 0.029455 -1.623 0.10495

offleash_activity30mins/1hour -0.033162 0.045322 -0.732 0.46450

offleash_activity1/3 -0.111645 0.044080 -2.533 0.01145 *

offleash_activity3/7 -0.046839 0.051902 -0.902 0.36701

offleash_activity>7 -0.062323 0.050981 -1.222 0.22179

BCSNormal -0.033834 0.036065 -0.938 0.34838

BCSOverweight -0.085235 0.046235 -1.843 0.06552 .

foodTinned and/or Dry food -0.105174 0.046973 -2.239 0.02535 *

foodCooked food 0.025453 0.038375 0.663 0.50729

foodMixed 0.061434 0.040280 1.525 0.12750

foodRaw 0.030642 0.042797 0.716 0.47415

Add_vitaminsRarely -0.044570 0.034224 -1.302 0.19308

Add_vitaminsOften -0.027740 0.038991 -0.711 0.47695

Add_vitaminsDaily 0.001044 0.040679 0.026 0.97953

traumaYes 0.081080 0.029182 2.778 0.00555 **

Health_probTooth problem only 0.120558 0.043610 2.764 0.00580 **

Health_probJoint & Tooth probs 0.051417 0.051753 0.994 0.32068

Health_probJoint problems only 0.067981 0.039599 1.717 0.08630 .

Health_probOther disorders 0.078293 0.042674 1.835 0.06682 .

medicationYes -0.017646 0.039003 -0.452 0.65106

Owner_age30-39 0.001533 0.034167 0.045 0.96423

Owner_age40-49 0.011271 0.037054 0.304 0.76104

Owner_age>50 0.020574 0.039194 0.525 0.59974

Age_of_dog_when_arrived7-12w 0.058252 0.034226 1.702 0.08903 .

Age_of_dog_when_arrived3-12m 0.033134 0.043910 0.755 0.45066

Age_of_dog_when_arrived>1y -0.070330 0.048209 -1.459 0.14489

get_dogborn bought 0.008325 0.046603 0.179 0.85825

get_dogpresent 0.014784 0.040445 0.366 0.71478

Where_dog_is_keptGarden -0.026773 0.045960 -0.583 0.56032

Where_dog_is_keptApartment -0.082921 0.042648 -1.944 0.05211 .

dog_obedience2 tasks 0.059101 0.050708 1.166 0.24406

dog_obedience3 tasks -0.055127 0.048526 -1.136 0.25619

dog_obedience>3 tasks -0.141124 0.046459 -3.038 0.00244 **

play30m-1h 0.017042 0.047721 0.357 0.72106

play1-3h -0.001272 0.048473 -0.026 0.97906

play>3h 0.029153 0.058571 0.498 0.61877

commands11-30 0.043670 0.032567 1.341 0.18022

commands>30 0.053949 0.052484 1.028 0.30422

training2-3 -0.013761 0.035681 -0.386 0.69982

training4 more 0.027331 0.041168 0.664 0.50689

dog_beh_changed_3monthYes 0.050355 0.031569 1.595 0.11097

---

Signif. codes: 0 ‘***’ 0.001 ‘**’ 0.01 ‘*’ 0.05 ‘.’ 0.1 ‘ ’ 1

Residual standard error: 0.4336 on 1118 degrees of freedom

(37 observations deleted due to missingness)

Multiple R-squared: 0.1176, Adjusted R-squared: 0.07735

F-statistic: 2.922 on 51 and 1118 DF, p-value: 1.205e-10
